# A Low-Power MEMS IDE Capacitor with Integrated Microhotplate: Application as Methanol Sensor using a Metal-Organic Framework Coating as Affinity Layer

**DOI:** 10.3390/s19040888

**Published:** 2019-02-20

**Authors:** Manjunath R. Venkatesh, Sumit Sachdeva, Brahim El Mansouri, Jia Wei, Andre Bossche, Duco Bosma, Louis C. P. M. de Smet, Ernst J. R. Sudhölter, Guo Qi Zhang

**Affiliations:** 1 Beijing Research Centre, Delft University of Technology, Mekelweg 4, 2628 CD Delft, The Netherlands; m.ramachandrappavenkatesh@tudelft.nl; 2 Department of Chemical Engineering, Delft University of Technology, Van der Maasweg 9, 2629 HZ Delft, The Netherlands; s.sachdeva.sumit@gmail.com (S.S.); d.bosma@tudelft.nl (D.B.); e.j.r.sudholter@tudelft.nl (E.J.R.S.); 3 Department of Microelectronics, Delft University of Technology, Mekelweg 4, 2628 CD Delft, The Netherlands; B.ElMansouri@tudelft.nl (B.E.M.); A.Bossche@tudelft.nl (A.B.); 4 Laboratory of Organic Chemistry, Wageningen University & Research, Stippeneng 4, 6708 WE Wageningen, The Netherlands; louis.desmet@wur.nl

**Keywords:** MEMS microhotplate, Capacitor interdigitated electrodes, ZIF-8

## Abstract

Capacitors made of interdigitated electrodes (IDEs) as a transducer platform for the sensing of volatile organic compounds (VOCs) have advantages due to their lower power operation and fabrication using standard micro-fabrication techniques. Integrating a micro-electromechanical system (MEMS), such as a microhotplate with IDE capacitor, further allows study of the temperature-dependent sensing response of VOCs. In this paper, the design, fabrication, and characterization of a low-power MEMS microhotplate with IDE capacitor to study the temperature-dependent sensing response to methanol using Zeolitic imidazolate framework (ZIF-8), a class of metal-organic framework (MOF), is presented. A Titanium nitride (TiN) microhotplate with aluminum IDEs suspended on a silicon nitride membrane is fabricated and characterized. The power consumption of the ZIF-8 MOF-coated device at an operating temperature of 50 ${}^{\circ }$C is 4.5 mW and at 200 ${}^{\circ }$C it is 26 mW. A calibration methodology for the effects of temperature of the isolation layer between the microhotplate electrodes and the capacitor IDEs is developed. The device coated with ZIF-8 MOF shows a response to methanol in the concentration range of 500 ppm to 7000 ppm. The detection limit of the sensor for methanol vapor at 20 ${}^{\circ }$C is 100 ppm. In situ study of sensing properties of ZIF-8 MOF to methanol in the temperature range from 20 ${}^{\circ }$C to 50 ${}^{\circ }$C using the integrated microhotplate and IDE capacitor is presented. The kinetics of temperature-dependent adsorption and desorption of methanol by ZIF-8 MOF are fitted with double-exponential models. With the increase in temperature from 20 ${}^{\circ }$C to 50 ${}^{\circ }$C, the response time for sensing of methanol vapor concentration of 5000 ppm decreases by 28%, whereas the recovery time decreases by 70%.

## 1. Introduction

Methanol is an organic solvent found in dyes, paints, perfumes and automotive fuel. Methanol is a colorless liquid, with a mild odor and flammable volatile organic compound (VOC). The threshold limit value (TLV) of exposure to methanol without causing adverse health effect is 200 ppm [1]. Monitoring of exposure to VOCs is important for health and well-being in an indoor air environment. Prolonged exposure to methanol for concentrations higher than TLV can cause headaches, drowsiness, and eye irritation. Methanol sensors developed using various metal-oxide semiconductors, such as SnO${}_{2}$, In${}_{2}$O${}_{3}$, α-Fe${}_{2}$O${}_{3}$ and ZnO operating at temperatures of 250 ${}^{\circ }$C–350 ${}^{\circ }$C require high-power transducers [1]. The development of a low-power sensor for the detection of methanol is not only interesting from a research point of view, but also useful for portable and wearable air quality systems.

In recent years, there has been much interest in the synthesis and development of porous materials such as zeolites and metal-organic frameworks (MOFs) for gas adsorption and sensing applications [2]. Metal-organic frameworks are crystalline materials formed of metal ions connected by organic linkers. The availability of various metal ions, organic linkers, and structure motif enables infinite topology combinations with unique chemical and physical properties [2]. MOFs as affinity layers is particularly interesting for various chemical vapors and gases due their high surface area, porous structure, tunability in material processing and development of thin film on electronic sensor devices [3]. Most MOFs inherently have low conductivity and the detection of gases has been mainly investigated using electronic devices such as chemi-resistive, chemi-capacitive cantilevers, quartz microbalance (QCM), and Field-effect transistors (FETs) [3,4]. Recent progress has been done in the development of conductive MOFs as affinity layers for chemi-resistive sensors [5,6,7].

Among the several MOF structures, Zeolitic imidazolate frameworks (ZIFs) are a class of MOF with metal ions (Zn, Co) with imidazolate linkers. They possess high porosity, ultra-high surface areas, and good thermal stability [8]. The adsorption and desorption of methanol have been investigated in the microporous structure of ZIF-8 MOF films deposited on silicon substrate [9]. ZIF-8 MOF consists of cages with an effective diameter of 12.5 Å and a hexagonal aperture of 3.3 Å. Methanol, with a kinetic diameter of 3.8 Å, can enter the microporous cage of ZIF-8 MOF and interact with the Zn atoms. On average, 2.7 molecules can adsorb per Zn atom [10]. ZIF-8 MOF have large surface area (1030 m${}^{2}$g${}^{-1}$) and high adsorption capacity of methanol, making it a suitable affinity layer for its investigation as a methanol sensor [10]. The detection of adsorbed alcohols using ZIF-8 MOF has been studied using several optical-based techniques [11,12]. ZIF-8 films grown on Fabry-Perot device enables detection of ethanol vapor by measuring the shifts in the interference of transmission spectrum [13]. Thin film ZIF-8 MOF-coated on optical fiber long period grating (LPG) has been shown to detect ethanol, methanol and acetone vapors in ranges up to 10,000 ppm [12]. However, ZIF-8 films as affinity layer for sensing of alcohols using techniques apart from optical-based techniques, such as capacitive sensing studies, have not been investigated. Detection of alcohols with thin film MOFs has been studied by measuring the changes in permittivity of the material using capacitive interdigitated electrode (IDE) transducers [14]. Cu paddlewheel MOFs using benzene-1,3,5-tricarboxylic acid as organic linkers (CuBTC) grown on IDE capacitor and NH${}_{2}$-MIL-53(Al) MOFs in Matrimid polymer matrix coated on IDEs show capacitive responses towards the detection of methanol at room temperature [14,15]. Owing to its simplicity of fabrication, compatibility with the complementary metal-oxide semiconductor (CMOS) process and availability of sensitive capacitance measurement systems, IDE capacitor transducers provide a reliable platform for sensing studies. Most studies with IDEs are done by measuring changes in capacitance at ambient temperature. A transducer with an IDE capacitor integrated with a micro-electromechanical system (MEMS) such as a microhotplate could enable the study of sensing performance as a function of temperature. The modeling of adsorption and desorption kinetics of gas analytes with temperature also enables development of temperature-modulated sensing operation. Using such a platform, the temperature-dependent capacitive sensing towards detection of methanol with ZIF-8 MOF as the sensing layer is studied in this paper.

A MEMS microhotplate integrated with parallel plate electrode has been used for on-chip curing of polyimide films at $350{\;}^{\circ }$C [16]. In this device, polyimide films were placed in between chromium electrodes forming a parallel plate and integrated with a microhotplate underneath the electrodes. The device was used for temperature-dependent capacitance and dielectric measurements. However, materials placed within parallel plate electrodes do not allow reliable measurements of changes in capacitance with exposure to air and various gaseous analytes. Interdigitated electrode capacitor with polysilicon heaters were used for the analysis of recovery and thermal reset of polyimide sensing film during humidity measurements [17,18]. A chemi-capacitive sensor with integrated molybdenum heater has been used for the study as CO${}_{2}$ sensor [19]. The power consumption of this heater was in the range of 237 mW at $75{\;}^{\circ }$C, which is high for continuous operation of portable battery powered electronic devices [19,20]. Thus, the development of IDE capacitor transducers with a low-power, integrated microhotplate would be a suitable device for in situ, temperature-dependent gas sensing measurements. In comparison with conductometric MEMS microhotplate platforms, the development of IDE capacitor with integrated microhotplate have challenges since capacitance measurements are sensitive to temperature, frequency, and parasitic capacitances variations. The changes in the isolation layer capacitance due to temperature also influences the overall measured capacitance. Hence, it is essential to consider the calibration factors due to the isolation layer capacitance during temperature-dependent capacitance measurements.

In the present work, a low-power MEMS microhotplate with IDE capacitor coated with thin film ZIF-8 MOF for the detection of methanol is studied. The device design, fabrication and experimental set up is discussed in Section 2.1, Section 2.2, Section 2.3, Section 2.4 and Section 2.5. In Section 3.2, thermal characterization of bare devices and devices coated with ZIF-8 MOF is presented. The temperature-dependent sensing response of ZIF-8 MOF-coated devices towards methanol, and water vapor is presented in Section 3.3. The modeling of temperature-dependent adsorption and desorption kinetic coefficients of methanol in ZIF-8 MOF is discussed in Section 3.4.

## 2. Experimental

### 2.1. Device Design

**Figure 1:**
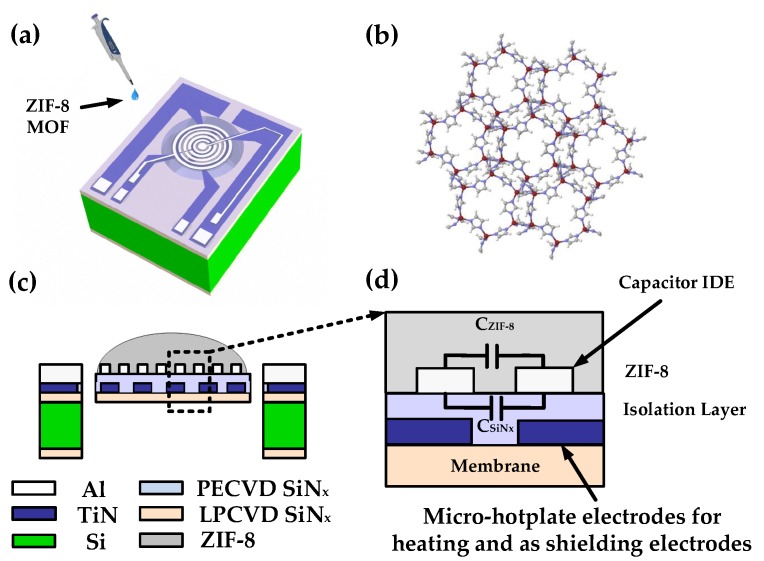
Device concept of ZIF-8 MOF-coated micro-electromechanical system (MEMS) interdigitated electrodes (IDE) capacitor with integrated microhotplate: (**a**) Illustration of ZIF-8 MOF drop-casted on the final device. (**b**) 3D view of the crystal structure of ZIF-8 MOF with Zn, N, C, and H described by red, purple, grey and white spheres respectively [27]. (**c**) Cross section of the device with deposited ZIF-8 MOF. (**d**) Description of equivalent electrical circuit of the IDE capacitor with microhotplate electrodes. PECVD: Plasma enhanced chemical vapor deposition; LPCVD: Low pressure chemical vapor deposition.

In this paper, a multilayered vertical stacked device structure is developed. The device concept and the cross-sectional view of the device is illustrated in Figure 1a,c, respectively. A circular microhotplate is fabricated on a thin dielectric membrane and forms the first metal layer. The IDE capacitor is fabricated above the circular microhotplate with an isolation layer between the two metal layers. The synthesized ZIF-8 MOF is deposited on the device by drop-casting as illustrated in Figure 1a. The capacitances obtained due to the thin film coating of ZIF-8 MOF on the device are, the isolation layer capacitance (C${}_{SiNx}$) and the capacitance of ZIF-8 MOF layer (C${}_{ZIF-8}$) (Figure 1d). The isolation layer is used to electrically insulate the two metal layers. However, the dielectric property of the isolation layer depends on both temperature and frequency of capacitance measurement. Thus, it is important to investigate the effects of the isolation layer capacitance (C${}_{SiNx}$) at different temperatures on the total measured capacitance of the bare device. This would serve as a capacitance calibration factor during temperature-dependent capacitance measurements of ZIF-8 MOF-coated devices. In addition to the calibration factor, a good shielding mechanism needs to be designed close to the IDEs to negate the parasitic effects of the substrate capacitance, interconnects, and bond pads capacitance. One possible approach followed in this study is by using the underlying microhotplate electrodes as a shielding electrode during capacitance measurement [21].

A microhotplate fabricated on a thin dielectric membrane with a temperature range up to $250{\;}^{\circ }$C is desirable since the organic linker imidazole present in ZIF-8 MOF has a thermal stability up to $257{\;}^{\circ }$C [22]. The key parameters considered in the design and fabrication of microhotplate are mainly temperature uniformity, power consumption and mechanical stability of the device [23]. Low-pressure chemical vapor deposited (LPCVD) silicon nitride (SiN${}_{x}$) exhibits tensile stress preventing buckling of the membrane at higher temperatures, thus making it a suitable choice as a membrane material in comparison to compressive stress exhibited by silicon dioxide [24]. The temperature uniformity of the device is greatly improved by efficient design of the microhotplate geometry. Many studies have investigated temperature uniformity of various microhotplate geometry that includes meander, spiral, double spiral, drive wheel, and honeycomb [23]. However, design guidelines for improving the temperature uniformity has been mainly investigated by variation of the ratio of the widths of the metal lines of a circular microhotplate [25]. By modifying this design guideline to enable four-probe IV measurements, a circular microhotplate is designed. In comparison with metals such as platinum and molybdenum as heating metal, titanium nitride (TiN) is a CMOS compatible material and capability of dry etching of TiN electrodes allows a simpler fabrication processing steps. The resistivity of TiN shows a linear dependence on temperature in the range up to 600 ${}^{\circ }$C [26]. Plasma enhanced chemical vapor deposited (PECVD) silicon dioxide and silicon nitride are considered for the isolation layer material. Silicon dioxide has compressive stress whereas silicon nitride used as membrane material exhibits tensile stress. To reduce the stress mismatch between the membrane and isolation layer, PECVD silicon nitride is used as the isolation layer material.

The design parameters used for the fabrication of the device are described in Table 1. Membranes with smaller diameter allow a reduction in power consumption but also decrease the area and the number of IDEs for capacitance measurement. To increase the measured base capacitance of the device with less influence from parasitic capacitances, a larger area for the circular IDE is required. Along with these considerations, the geometry parameters that determine the capacitance of circular IDEs are the width of the electrodes (*W*), the gap between the electrodes (*G*) and the number of electrode pairs (*N*) [28]. The device dimensions were thus optimized to enable lower power consumption of the device and larger area for the capacitor electrodes. A circular LPCVD (SiN${}_{x}$) membrane with a diameter of 1mm and thickness of 500nm supported by four beams is designed. A circular TiN microhotplate with a thickness of 400nm is deposited and patterned on the (SiN${}_{x}$) membrane. Within the membrane dimensions, the number of circular IDE capacitor pairs (*N*) designed is 164. The optimized dimensions for the width of the capacitor IDEs within the process limitations are *W* = 2 μm and gap between the electrodes *G* = 1 μm.

### 2.2. Device Fabrication

**Figure 2:**
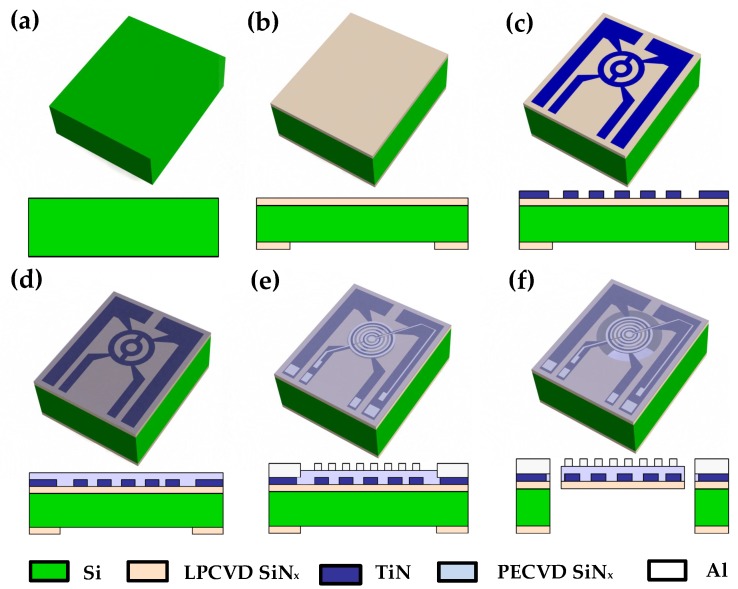
Fabrication process flowchart of the device (**a**) Silicon substrate. (**b**) 500 nm low-stress LPCVD silicon nitride. (**c**) Patterned 400 nm Ti/TiN microhotplate on the front side of the wafer. (**d**) 1 μm PECVD silicon nitride isolation layer on top of the microhotplate. (**e**) Patterned 1 μm Al/1% Si IDEs. (**f**) deep reactive ion etching (DRIE) etching of Si to obtain a suspended membrane.

A double-sided polished 300 μm thick 4-inch p-type <100> silicon wafer is used as the starting material (Figure 2a). A layer of low-stress LPCVD SiN${}_{x}$ of thickness 500 nm is deposited both sides of the wafer using a Tempress LPCVD furnace. A 4 μm thick PECVD silicon dioxide (SiO${}_{2}$) is deposited and patterned on the back side of the wafer. This is used as a hard mask during back side deep reactive ion etching (DRIE). A 350 nm thick TiN metal layer with Titanium (Ti) of thickness 50 nm as the adhesion layer is deposited by sputtering on the front side of the wafer. The TiN heater design is etched by a plasma etching process using a Trikon Omega 201 plasma etcher (Figure 2c). In the next step, an isolation layer of 1 μm of PECVD SiN${}_{x}$ is deposited on top of the TiN electrodes (Figure 2d). Next, a layer of aluminum (Al 1% Si) with a thickness of 1 μm is sputtered on PECVD SiN${}_{x}$. The electrodes are etched by plasma etching to form the IDEs (Figure 2e). This isolation layer electrically isolates the TiN heater electrodes and the aluminum (Al) IDE capacitor. Finally, DRIE of silicon is performed on the backside of the wafer to release the suspended membrane (Figure 2f).

### 2.3. Synthesis of ZIF-8 MOF

All chemicals were purchased from Sigma Aldrich and used as received. Typically, 734.4 mg (2.469 mmol) of Zn(NO${}_{3}$)${}_{2}$·6H${}_{2}$O and 810.6 mg (9.874 mmol) of 2-methylimidazole (Hmim) are each dissolved in 50 mL of methanol (MeOH). The latter clear solution is poured into the former clear solution under stirring with a magnetic bar. Stirring is stopped after combining the component solutions. After 24 h, the solid is separated from the milky colloidal dispersion by centrifugation. Washing with fresh MeOH and centrifugation is repeated three times [29]. The product is dried at room temperature under reduced pressure. The synthesized ZIF-8 MOF is dispersed in ethanol and ultrasonicated to obtain a uniform suspension of 4.5 wt% ZIF-8 MOF in ethanol solution. The prepared solution is drop-casted on the devices using a 1 μL pipette.

### 2.4. Structural Characterization

Scanning Electron Microscopy (SEM) images were acquired at different magnification using JEOL JSM 6010LA (JEOL, Tokyo, Japan) and Philips XL50, (FEI/Thermo Fisher Scientific, Waltham, MA, USA). SEM images were acquired after sputtering with gold layer for a device coated with ZIF-8 MOF using JEOL JFC-1300 (JEOL, Tokyo, Japan) auto fine coater with current setting at 20 mA for 120 s. X-Ray Diffraction (XRD) of as-synthesized ZIF-8 MOF were recorded using Bruker D8 Advance diffractometer with Co-K radiation (λ = 1.788897 Å). A step size of 0.02${}^{\circ }$ with a scan speed of 0.2 s per step was used to acquire the diffraction pattern. The measurement of the thickness of the deposited ZIF-8 MOF on the devices was done using Keyance laser profilometer. Optical images were acquired with SENTECH STC-625CC-CM (Carrollton, TX, USA) camera connected to a Cascade microtech probe station.

### 2.5. Experimental Setup and Measurement Procedure

**Figure 3:**
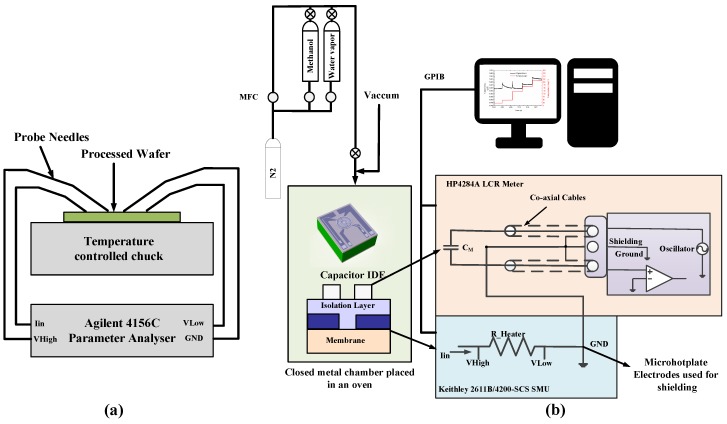
(**a**) Electrical characterization of the microhotplate. (**b**) Schematic representation of the gas sensing measurement setup. MFC: Mass Flow controller; GPIB: General Purpose Interface Bus; GND: Ground.

A thermochuck temperature controller, connected to a Cascade Microtech wafer probe station and Agilent 4156C (Keysight Technologies, Santa Rosa, CA, USA) parameter analyzer, was used for the electrical characterization of the TiN microhotplates at different temperatures (Figure 3a). In this experiment, the temperature of the chuck increased in eight temperatures steps in the range from 20 ${}^{\circ }$C to 200 ${}^{\circ }$C. A small current of 50 μA was supplied through the TiN microhotplate (to avoid self-heating of the microhotplate), and the voltage drop across the microhotplate was measured using an Agilent 4156C parameter analyzer at each temperature steps. The temperature coefficient of resistance (TCR) of the devices were extracted by this measurement. This calibration data allows monitoring of the average temperature of the devices during device operation.

The steady-state power consumption of the devices before and after deposition of ZIF-8 MOF was measured in vacuum and nitrogen. This was done to understand the thermal performance of the devices under different conditions. The device was wire-bonded and packaged in a 40-pin dual-inline package. The packaged device was sealed in a closed metal chamber with an electrical feed-through and placed in an oven. A schematic of the experimental setup for the measurement of the devices is conceptually illustrated in Figure 3b. A Keithley 2611B/4200-SCS (Tektronix, Berkshire, UK) source measurement unit (SMU) was used for controlling the current flow through the microhotplate. The voltage across the microhotplate was measured, and thereby the resistance and corresponding operating temperature were determined. The chamber was connected to a vacuum pump, enabling a low-pressure control down to 1 mbar. A current sweep from 0.2 mA up to 5 mA was used to characterize the heater performance in vacuum (1 mbar), while a current sweep from 0.2 mA up to 8mA was used for nitrogen (1 bar) environment.

The capacitance measurement of the device is very sensitive to parasitic capacitances due to the connections. Thus, a good shielding between the devices, HP4284A LCR meter (Keysight Technologies, Santa Rosa, CA, USA), and Keithley SMU must be made. The connections to the LCR meter and Keithley use SMA connectors which were shielded to the system ground. The outer conductors of the coaxial cables are shielded to the system ground to avoid measurement errors due to interference. The TiN microhotplate device below the IDE capacitor provides a shielding mechanism during measurement of the capacitance. The DC ground of the microhotplate was coupled to the ground shielding of the LCR meter for shielding the IDE capacitor (Figure 3b). The capacitance characterization of the bare device was done in vacuum at different operating temperatures using the microhotplate. The current flow through the microhotplate was controlled using the Keithley 2611B/4200-SCS for operating the microhotplate at the desired temperature. The capacitance was simultaneously measured using an HP4284A LCR meter at a constant frequency of 10 kHz and oscillation voltage of 1 V.

Sensing experiments of the packaged ZIF-8 MOF-coated device with vapors of methanol and water was done in a chamber as described in Figure 3b. Before the measurements, dry nitrogen was introduced until a stable baseline was obtained. The vapors of water and methanol were generated through a series of two bubblers to attain a saturated stream of the analyte as described in our previous works [14]. The vapors were then diluted with a parallel stream of dry nitrogen and passed over the packaged device in the chamber at a constant flow rate of 200 mL/min. The corresponding changes in the capacitance for exposure to different concentration was measured with the HP4284A LCR meter at a frequency of 10 kHz and oscillation voltage of 1 V. Using the integrated microhotplate enabling in situ heating, the temperature-dependent sensing response of ZIF-8 MOF was obtained for methanol and water vapor.

## 3. Results and Discussion

### 3.1. Device and Material Characterization

**Figure 4:**
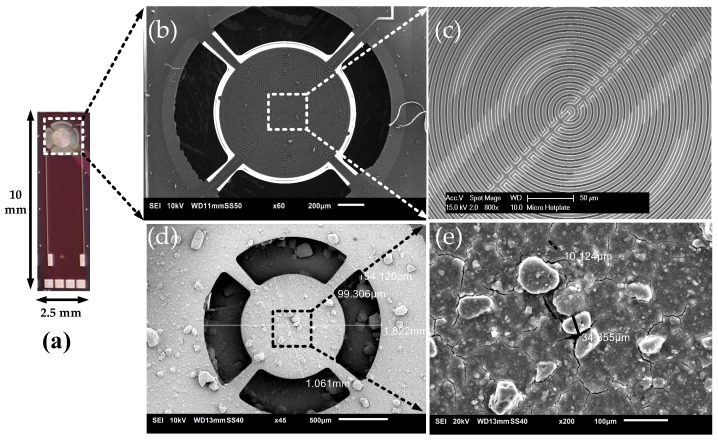
(**a**) Fabricated device. (**b**) SEM image of the suspended microhotplate with capacitor IDE. (**c**) A close-up image showing the active area of the device. (**d**) ZIF-8 MOF-coated device (**e**) A close-up image of ZIF-8 MOF on top of the electrodes.

The synthesized MOF was examined for morphology and crystallinity by SEM and XRD. The rhombic dodecahedral crystals and the diffraction pattern of the obtained MOF showed similarity with ZIF-8 as indicated by the theoretically simulated pattern of ZIF-8 (Supplementary Information, Figure S1a,b), and as reported by literature [30,31]. It clearly demonstrated that the synthesis of ZIF-8 was successful, and was then further used for deposition over the active area of the devices. The device with a dimension of 10 mm × 2.5 mm with the active area at the top and contact pads at the bottom is shown in Figure 4a. The bare device with patterned circular aluminum IDEs with a width (*W*) of 2 μm and gap (*G*) of 1 μm above the TiN microhotplate (darker electrodes) is shown in Figure 4c. The as-synthesized ZIF-8 MOF is deposited on the device area. Figure 4d,e shows that the IDEs is completely covered with ZIF-8 MOF. The thickness of ZIF-8 drop-casted on the devices was measured along the diagonal profile of the device as shown in Supplementary Information, Figure S2. The thickness obtained was larger than 3 μm and the average thickness is 20 μm. It is seen that due to drying effects, there is slightly less coating at the center of the device. In the current design of the IDE capacitor, the width (*W*) = 2 μm and gap (*G*) = 1 μm, so the spatial wavelength (λ) = 6 μm. The thickness of the affinity layer should be greater than half the spatial wavelength (>0.5 * λ) [32]. The obtained thickness of the coating was about 6–10 times the half spatial wavelength (0.5 * λ), verifying that the thickness of the affinity layer is large enough to completely enclose the electrical field lines. In such case, no performance change in the equilibrium/static response will be observed [14,32]. The response will only be affected with respect to diffusion of molecules to sensitive region of the device. However, the density of the film might change with thickness, because drop-casting does not control the density/void spaces of the deposited layer. The stability of density/void spaces of the deposited film on the device was further verified by characterizing the sensor to the analyte for several days.

### 3.2. Thermal Characterization

In this section, the TCR of the fabricated TiN microhotplate device is extracted. The steady-state power consumption of the bare device and the device coated with ZIF-8 MOF in nitrogen and vacuum is measured and compared. Next, thermal modeling using Finite Element Method (FEM) is done to analyze the temperature distribution across the ZIF-8 coated device.

**Figure 5:**
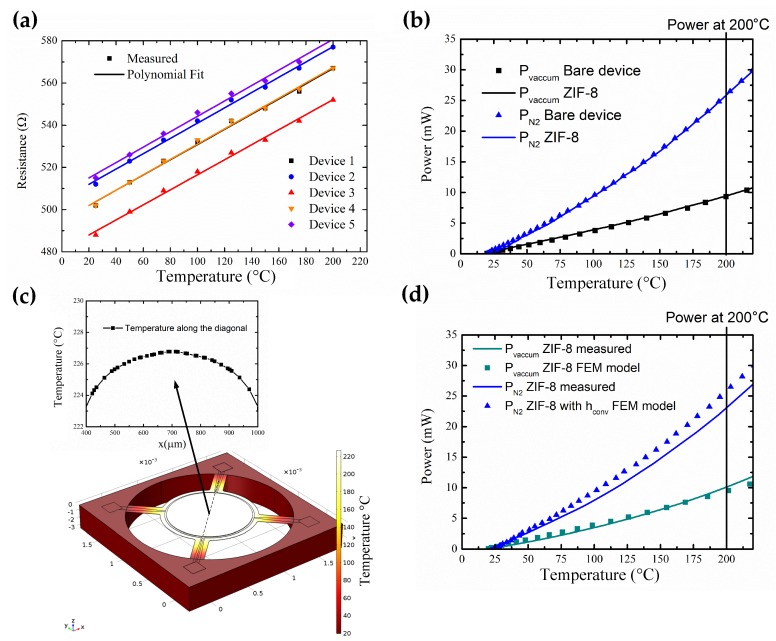
(**a**) Power consumption in bare device. (**b**) Comparison between power consumption in bare device and ZIF-8 coated device in vacuum and nitrogen. (**c**) Finite Element Method (FEM) thermal analysis of ZIF-8 coated device. (**d**) Comparison between power consumption of ZIF-8 coated device and FEM results.

The measured resistance of the TiN microhotplate as a function of temperature for five devices is shown in Figure 5a. The average resistance at 20 ${}^{\circ }$C is 500 Ω. The TCR (α) of the heater is extracted by the below equation,

(1) $$R\left(T\right)=R_{0}\cdot \left(1+\alpha \cdot \left(T-T_{0}\right)\right),$$

where *R${}_{0}$* is the resistance at room temperature and *R(T)* is the resistance at temperature *T*. From the Figure 5a, it is seen that due to process variation, there is a slight difference in the values of *R${}_{0}$* of the samples. However, the slope, and thus the TCR, of the microhotplate is nearly constant. The average TCR of five devices is 0.000717/${}^{\circ }$C. The thermal characterization of bare device in vacuum and nitrogen, and heat losses in the device is analyzed in Section 3.1, Supporting Information.

The steady-state power consumption of the device coated with ZIF-8 MOF and the bare device is shown in Figure 5b. The input power required to attain an operating temperature of 200 ${}^{\circ }$C with ZIF-8 coated device is 26 mW in comparison to the bare device which is 25.8 mW (Supporting Information, Figure S3). The small increase in input power for ZIF-8 MOF-coated device could be due to the extra MOF coated on the suspension, which decreases the thermal resistance slightly for heat loss due to conduction. The achieved power consumption for the device coated with ZIF-8 MOF at 200 ${}^{\circ }$C is much lower than the reported IDE capacitor transducer with integrated microhotplate as shown in Table 2.

To further analyze the temperature uniformity across the device, a FEM model of the device with joule heating module is developed in Section 3.2, Supporting Information, using COMSOL Multiphysics^®^ version 5.3 (COMSOL Inc., Stockholm, Sweden). The extracted convection coefficient for the device in nitrogen (*h*${}_{conv}$), TCR of the TiN microhotplate (α) obtained from the above measurements are used in the joule heating module of the multiphysics model. The temperature uniformity obtained from FEM analysis across the active region of the membrane coated with ZIF-8 MOF is +/−4 ${}^{\circ }$C (Figure 5c). The comparison between the steady-state power consumption of the FEM model and measured results with ZIF-8 MOF is shown in Figure 5d. The observed difference between power consumption versus temperature of simulated and measured results at a temperature of 200 ${}^{\circ }$C is found to be 7% in vacuum, and in nitrogen it is 11%. This shows that the FEM thermal modeling follows closely to expected measurement results. The observed difference can be attributed to the deviations in the reference thermal conductivity values of the materials and non-ideal extraction of the convection coefficient [33].

With the capability of in situ heating, thermal stability tests of ZIF-8 MOF were done at various temperatures to observe the physical changes in ZIF-8 MOF. The surface morphology of ZIF-8 MOF at 50 ${}^{\circ }$C and 100 ${}^{\circ }$C is shown in Supplementary Information, Figure S5b,c respectively. The degradation of ZIF-8 MOF film is not observed optically at these low temperatures. The changes in the morphology and distribution of the ZIF-8 thin film is clearly visible on heating from 20 ${}^{\circ }$C to 300 ${}^{\circ }$C as shown in Supplementary Information, Figure S5d. This shows the thermal limitation of ZIF-8 MOF which coincides with the reported TGA experiments [36].

### 3.3. Sensing Measurements

**Figure 6:**
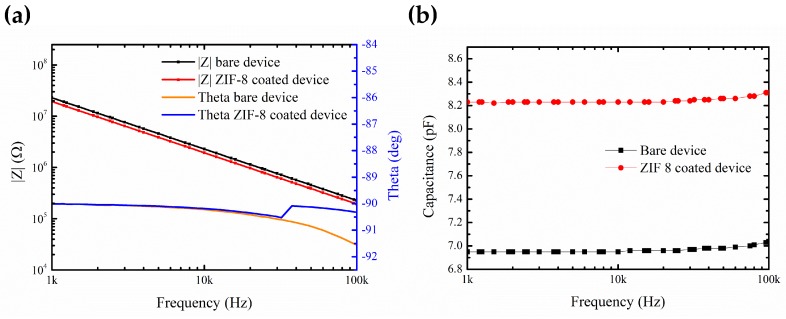
(**a**) Impedance and Theta versus frequency. (**b**) Comparison between capacitance of bare and ZIF-8 coated device versus frequency.

The impedance characterization of both the bare device and ZIF-8 coated device was done in the frequency range from 1 kHz to 100 khz. It is seen that the deposition of ZIF-8 MOF on the devices causes a decrease in the overall impedance measured and increase in the capacitance as shown in Figure 6a,b. The values of theta of the devices lie within a small range around −90${}^{\circ }$ for frequencies of 1 kz–100 Khz (Figure 6a), indicating the capacitive nature of the devices. In more detail, this indicates that the addition of ZIF-8 thin film MOF on top of the IDE still shows capacitive behavior similar to previous studies of ZIF-8 MOF grown on top of silicon substrate [9]. A stable capacitance response is seen in the frequency range from 1 kHz to 100 kHz (Figure 6b).

**Figure 7:**
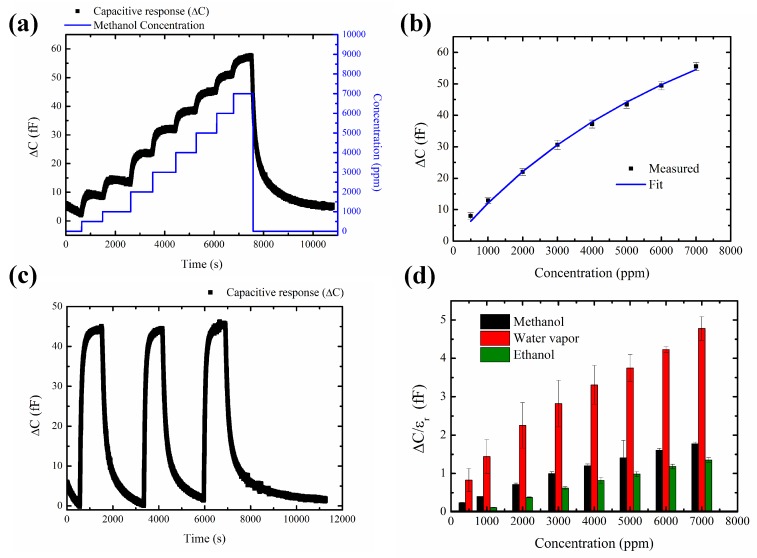
(**a**) Sensing response for methanol concentration from 500 ppm to 7000 ppm. (**b**) Langmuir isotherm fit. (**c**) Response to 5000 ppm of methanol. (**d**) Comparison between methanol, ethanol, and water vapor normalized with the dielectric constant.

The device coated with ZIF-8 MOF was exposed to increasing concentration of methanol from 500 ppm to 7000 ppm in dry nitrogen. The resulting changes in capacitance was measured at an oscillation voltage of 1V and a frequency of 10 kHz. Methanol, with a kinetic diameter of 3.4 Å, and a dielectric constant of ($\epsilon_{r}$ = 32) can enter and condense within the flexible porous structure of ZIF-8 MOF with a pore diameter of 3.8 Å [9]. The adsorption of methanol in ZIF-8 MOF increases the effective dielectric constant resulting in the increase in the measured capacitance. The observed changes in the measured capacitance are in low fF ranges (60 fF). A baseline drift was observed during the sensing study (Section 5, Supporting Information, Figure S6a). The baseline drift was calibrated, and the resulting change in capacitance (Δ*C*) for increasing concentration of methanol is shown in Figure 7a. The capacitance of the device (Δ*C*) changes from 9–60 fF for methanol concentration in the range from 500–7000 ppm (Figure 7b). The changes in the measured capacitance (Δ*C*) in ZIF-8 MOF for increasing methanol concentration is fitted with Langmuir adsorption isotherm model given by [14],

(2) $$\frac{\Delta C}{C_{s}}=\frac{K_{e}C_{m}}{1+K_{e}C_{m}},$$

where *C*${}_{s}$ is the saturation value of the capacitance, *K*${}_{e}$ is the affinity constant and *C*${}_{m}$ is the concentration of methanol. The obtained values for *K*${}_{e}$ and *C*${}_{s}$ are 101 bar${}^{-1}$ and 131.7 fF, respectively. The saturation value of the capacitance (*C*${}_{s}$) is obtained at much higher methanol concentration indicating a higher capability of adsorption of methanol in the porous framework of ZIF-8 MOF [12]. The calculation of the minimum detection of the capacitance using the current system is done by considering the baseline variation of capacitance in nitrogen as shown in Figure S7, Supporting Information. The standard deviation σ is 0.3 fF. Considering 3σ value, the minimum detectable capacitance change on exposure to methanol for this device is about 1 fF. The detection limit of the sensor for methanol with a capacitance change of 1 fF is 100 ppm. The obtained sensing performance can be further improved by 1000 fold using sensitive capacitive read-out system with lock-in principle for aF-level capacitance detection [37]. However, development of sensitive capacitance read-out system was not the focus of our study. A comparison of various methanol sensors in literature over a wide concentration range with sensing materials such as metal-oxide/metal-oxide composites, MOFs and Epoxy acrylate polymer film is shown in Table 3. In metal-oxide/metal-oxide composite sensing materials, methanol vapor mainly reacts with the chemisorbed oxygen on the surface of the film at high temperature (150 ${}^{\circ }$C–350 ${}^{\circ }$C), thus requiring higher power during sensor operation. However, sensing materials such as CuBTC-MOF, NH${}_{2}$-MIL-53(Al) MOF in Matrimid polymer and Epoxy acrylate film show methanol sensing responses at near room temperature. In terms of operating temperature, concentration range and detection limit the performance our devices coated with ZIF-8 MOF is comparable with the best result presented in Table 3 (first entry, reference [38]). Interestingly, also this study makes use of a MOF, but the sensor design is different: while we make use of an IDE platform onto which a MOF coating is prepared, Homayoonnia and Zeinali prepare the second electrode on top of the MOF coating to obtain a sandwich structure. Moreover, IDE capacitor transducers also allow simpler integration of MOFs on the devices and package development of the sensors. In conclusion, Table 3 shows the potential of MOFs in the further development of miniaturized low-power sensors.

The reversibility behavior of methanol at a concentration of 5000 ppm after baseline drift correction (Supporting Information, Figure S8a,b) is shown in Figure 7c. It can be seen that methanol adsorbed by ZIF-8 is completely reversible in dry nitrogen. Next, a comparative study for increasing concentration of methanol, ethanol and water vapor from 500 ppm to 7000 ppm is performed. The obtained capacitance response (Δ*C*) is corrected with the dielectric constant (Δ*C*/$\epsilon_{r}$) for water ($\epsilon_{r}$ = 78), methanol ($\epsilon_{r}$ = 32) and ethanol ($\epsilon_{r}$ = 24.2) as shown in Figure 7d. The response to ethanol is found to be less than methanol. This is due both to concentration being taken up in the ZIF-8 film, and to the contribution to the change of dielectric constant. Ethanol, with a lower dielectric constant than methanol, thus contributes to lower signal strength. The measured response (Δ*C*/$\epsilon_{r}$) to water vapor is found to be higher than the response towards methanol in the concentration range from 500 ppm to 7000 ppm. Water molecules with a higher dielectric constant ($\epsilon_{r}$ = 78) and kinetic diameter of 2.8 Å, can enter and condense in the porous framework of ZIF-8 MOF [9], resulting in higher changes in the measured capacitance.

### 3.4. Temperature-Dependent Adsorption and Desorption Kinetics

The device coated with ZIF-8 MOF was operated at a constant temperature by the TiN microhotplate at 20 ${}^{\circ }$C, 30 ${}^{\circ }$C, 40 ${}^{\circ }$C and 50 ${}^{\circ }$C. The resulting change in capacitance (ΔC) for a constant concentration of 5000 ppm of methanol and water vapor was measured.

**Figure 8:**
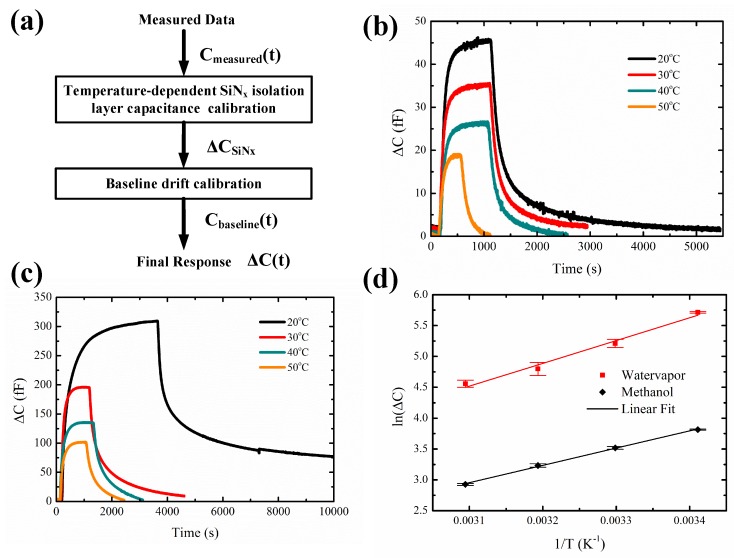
(**a**) Data analysis procedure. Capacitance response at different temperatures for 5000 ppm of (**b**) Methanol, (**c**) Water vapor, (**d**) Arrhenius relation between capacitance and temperature.

The data analysis procedure for the measured response is described in Figure 8a and the below equation,

(3) $$\Delta C\left(t\right)=C_{measured}\left(t\right)-\Delta C_{SiN_{x}}-C_{baseline}\left(t\right).$$

The measured capacitance response ($C_{measured}$(*t*)) is first calibrated with temperature-dependent isolation layer. The calibration curve obtained for $\Delta C_{SiN_{x}}$ versus temperature is explained in detail in Section 6, Supplementary Information. Next, a baseline drift calibration $C_{baseline}\left(t\right)$ is done for both methanol (Section 7, Supplementary Information, Figure S18a–i) and water vapor (Section 7, Supplementary Information, Figure S19a–i). The final response ΔC(*t*) for different operating temperatures from 20 ${}^{\circ }$C to 50 ${}^{\circ }$C for methanol and water vapor is shown in Figure 8b,c, respectively.

The change in capacitance (ΔC) for both methanol (Figure 8b) and water vapor (Figure 8c) decreases with the increase in temperature. At a temperature of 20 ${}^{\circ }$C the saturation value of the change in capacitance ΔC is 45fF, whereas at 50 ${}^{\circ }$C the saturation value of ΔC decreases to 18 fF. In the case of water vapor, a similar decrease in the saturation value of capacitance is observed at increased temperatures. The saturation value of ΔC obtained at 20 ${}^{\circ }$C is 300 fF and it reduces to 100 fF at 50 ${}^{\circ }$C. These results indicate that the amount of analyte adsorbed by ZIF-8 MOF decreases with increasing temperatures. The adsorption process is further studied by determining the enthalpy of adsorption. An Arrhenius plot of the change in capacitance versus temperature is shown in Figure 8d. This behavior is described by the following equation [14],

(4) $$\Delta C=C_{max}e^{\frac{-\Delta H}{RT}},$$

where $C_{max}$ represents the equilibrium capacitance, (Δ*H*) is the difference in the activation energy of the adsorption and desorption process. The calculated values of enthalpy of adsorption (Δ*H*) is −23 kJ/mol for methanol and for water vapor it is −30 kJ/mol. The negative value obtained for Δ*H* indicates the exothermic nature of the adsorption process. The slightly higher enthalpy of adsorption for water vapor observed at low water uptake is due to the structural defects present in the ZIF-8 MOF that increases the interaction energy with water vapor molecules [44]. The sensing measurements were performed for several weeks for testing the long-term stability of ZIF-8 MOF. The response to 5000 ppm of methanol at 20 ${}^{\circ }$C in a three-week period is shown in Figure S20, Supporting Information. The observed changes in Δ*C* is less than 3 fF. The measured response to methanol concentration of 5000 ppm at different temperature done once every week for three weeks, (Supporting Information, Figure S21) show good stability of the ZIF-8 MOF with temperature up to 50 ${}^{\circ }$C. These measurements also indicate that the density of the deposited film by drop-casting process on the device does not change over a period of time.

**Figure 9:**
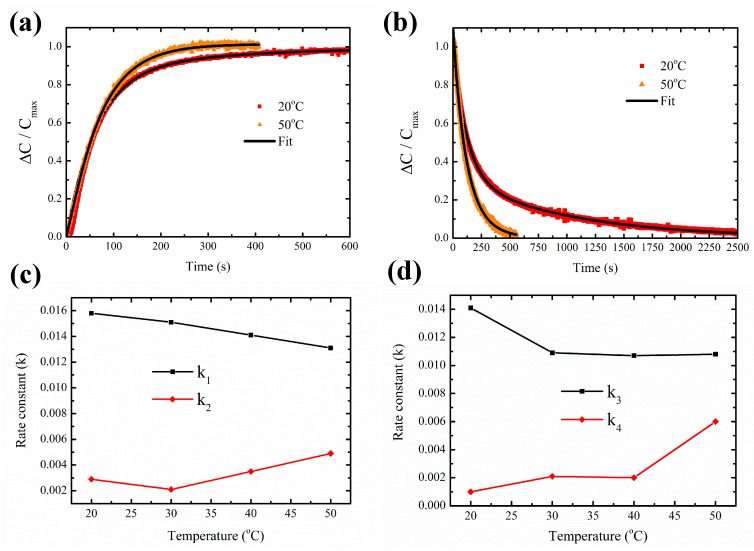
Time-dependent capacitance response at 20 ${}^{\circ }$C (red) and 50 ${}^{\circ }$C (orange) with Langmuir-based double-exponential fitting (black) for (**a**) adsorption of methanol and (**b**) desorption of methanol. Kinetic parameters versus temperature; (**c**) adsorption of methanol; (**d**) desorption of methanol.

Based on the capacitance response and recovery curves at different temperatures, the time-dependent adsorption and desorption kinetics can be derived. The measured capacitance at 20 ${}^{\circ }$C and 50 ${}^{\circ }$C normalized to the equilibrium capacitance (($\Delta C)/C_{max}$) for adsorption and desorption of methanol is given in Figure 9a,b, respectively. The Langmuir adsorption and desorption kinetics of gases in MOFs and porous materials are described using Double-Exponential Models (DEP) in various studies [45,46,47]. In this model, adsorption is based on a two-stage process due to the energetic barriers during diffusion of vapor molecules in MOFs. The first energetic barrier is due to the diffusion through the windows of the porous framework and the second barrier is diffusion along the pore cavities. With increasing temperature, the rate of adsorption of methanol to attain equilibrium capacitance increases (Figure 9a). The DEP curve fitting model is given by the following equation

(5) $$\frac{M_{t}}{M_{s}}=A_{1a}\left(1-e^{-k_{1}}\right)+A_{2a}\left(1-e^{-k_{2}}\right),$$

where $M_{t}$ represents the mass uptake at time *t*, $M_{s}$ is the mass uptake at equilibrium, $k_{1}$ and $k_{2}$ represents the kinetic rate constants and $A_{1a}$ and $A_{2a}$ are the relative contribution of the two energetic barriers during the adsorption process.

In Figure 9b, a higher rate of desorption of methanol is observed at higher temperatures. The desorption process can also be modeled as a two-stage process with a fast exponential decay in the first stage followed by slow decay in the second stage. The modeling equation is given by

(6) $$\frac{M_{d}}{M_{o}}=A_{1d}\left(e^{-k_{3}}\right)+A_{2d}\left(e^{-k_{4}}\right),$$

where $M_{d}$ represents the mass desorbed at time *t*, $M_{o}$ is the initial mass adsorbed at equilibrium, $k_{3}$ and $k_{4}$ are the kinetic desorption rate constants and $A_{1d}$ and $A_{2d}$ are the relative contribution of the rate constants during the desorption process.

In ZIF-8 MOF, the capacitive sensing response towards methanol follows Langmuir adsorption isotherm behavior as described in Section 3.4. Thus, the time-dependent adsorption and desorption process described in Equations (5) and (6) can be further related to the time-dependent capacitance changes during sensing and recovery measurement. The capacitance response kinetics for the adsorption (Equation (7)) and desorption process (Equation (8)) are described below:

(7) $$\frac{\Delta C}{C_{max}}=A_{1a}\left(1-e^{-k_{1}}\right)+A_{2a}\left(1-e^{-k_{2}}\right),$$

(8) $$\frac{\Delta C}{C_{max}}=A_{1d}\left(e^{-k_{3}}\right)+A_{2d}\left(e^{-k_{4}}\right).$$

The regression coefficients for the curve fitting of DEP models for sensing and recovery curves at different temperatures are $R^{2}$ > 99% (Section 9, Supplementary Information). The time-dependent adsorption and desorption kinetic parameters for different temperatures are tabulated in Table 4. Based on the sensing and recovery curves, the response time and recovery time is calculated for different temperatures. The response time ($t^{+}$) is defined as the time taken to reach 90% of the equilibrium capacitance value ($C_{max}$) and the recovery time ($t^{-}$) is defined as the time taken to reach 10% of the equilibrium capacitance value. The values for the response time and recovery time for different temperatures is given in Table 4. It can be seen that during the adsorption process the obtained kinetic rate constants $k_{1}$ is greater $k_{2}$. Upon increasing temperatures, the rate constant $k_{1}$ decreases whereas $k_{2}$ slightly increases. The overall response time decreases with the increase in temperatures. At a temperature of 20 ${}^{\circ }$C the response time is 207 s, whereas at 50 ${}^{\circ }$C the response time decreases to 147 s. During the desorption process, it is seen that the kinetic parameter k${}_{3}$ shows a decrease with increasing temperatures whereas k${}_{4}$ increases with temperature. The recovery time decreased at higher temperatures, at 20 ${}^{\circ }$C the recovery time is 981 s, at 50 ${}^{\circ }$C the recovery time obtained is 298 s. With the increase in temperature, changes in the pore structure of the ZIF-8 MOF can occur influencing the diffusivity of gases within the porous structure of ZIF-8 MOF [48]. These structural changes could lead to the observed overall decrease in both response and recovery time as the temperature increases.

**Figure 10:**
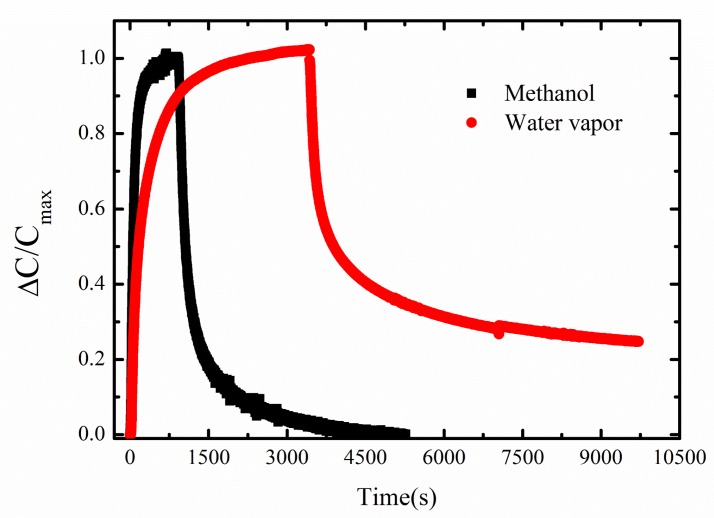
Methanol and water vapor sensing and recovery curves at 5000 ppm at 20 ${}^{\circ }$C.

Next, a comparison between the time-dependent methanol and water vapor adsorption and desorption at 20 ${}^{\circ }$C for a constant concentration of 5000 ppm of the analyte is shown in Figure 10. It can be seen that methanol has a higher rate of adsorption and desorption in ZIF-8 MOF in comparison to water vapor. The response time obtained for methanol and water vapor at 20 ${}^{\circ }$C is 207 s and 913 s, respectively. The recovery time obtained for methanol is 981 s at 20 ${}^{\circ }$C and for water vapor the recovery time is more than 3000 s. This shows that ZIF-8 MOF is kinetically selective for the adsorption of methanol than water vapor.

## 4. Conclusions

In this paper, we report a successful demonstration of ZIF-8 MOF capacitive sensor with integrated TiN microhotplate. The sensor shows changes in capacitance on exposure to methanol from 500 ppm–7000 ppm, with a capability for the detection of 100 ppm of methanol vapor at a temperature of 20 ${}^{\circ }$C. Cross-sensitivity study with exposure to water vapor in the concentration from 500 ppm–7000 ppm shows that water vapor has a higher capacitance response. However, at a temperature of 20 ${}^{\circ }$C it is seen that the adsorption of methanol is faster than water vapor for a constant concentration of 5000 ppm. The changes in the morphology of ZIF-8 MOF was investigated by in situ heating from 20 ${}^{\circ }$C to 300 ${}^{\circ }$C using the TiN microhotplate. A systematic experiment methodology was followed in this paper with careful consideration of the capacitance changes with temperature due to the PECVD silicon nitride isolation layer on the overall measured capacitance. The change in the capacitance of the bare device was found to be linear with temperature, and it is used as a calibration factor during sensing study with temperature. In situ heating using integrated microhotplate enabled the study of the kinetics of adsorption and desorption of methanol with temperatures from 20 ${}^{\circ }$C to 50 ${}^{\circ }$C. The temperature-dependent kinetic rate constants for both adsorption and desorption of methanol were derived. With increasing temperature from 20 ${}^{\circ }$C to 50 ${}^{\circ }$C, the response time decreased from 207 s to 147 s. A similar decrease in the recovery time was obtained from 981 s to 298 s at 20 ${}^{\circ }$C and 50 ${}^{\circ }$C, respectively. The ability for fast temperature cycle times using the microhotplate allows for the determination of the optimized temperature and reset time of the ZIF-8 MOF-coated devices during capacitive vapor sensing measurements. An array of such developed devices also allows the possible solution to combine different types of MOFs as affinity layers with different selectivity. This is an essential step towards a multi-sensing platform.

## Figures and Tables

**Table 1:** Design parameters for fabrication of the devices.

Design Parameter	Value	Unit
Thickness of silicon nitride membrane	500	nm
Diameter of membrane	1	mm
Thickness of silicon nitride isolation layer	1	μm
Thickness of TiN electrodes	400	nm
Thickness of Al electrodes	1	μm
Width of IDE (*W*)	2	μm
Gap between IDE (*G*)	1	μm
Number of IDE pairs (*N*)	164	-

**Table 2:** Comparison of power consumption and operating temperature of MEMS capacitor sensors with integrated microhotplate.

Device Temperature (${}^{\circ }$C)	Power (mW)	Material Studied	Reference
75	237	3-AMO and 30% PTMS	[19]
60	300	Similar to [19]	[34]
50	37	Polyimide	[17]
46	155	Polyimide	[35]
200	26	ZIF-8	This Work

${}^{1}$ aminopropyltrimethoxysiloxane(AMO); ${}^{2}$ propyltrimethoxysilane(PTMS).

**Table 3:** Comparison of the developed ZIF-8 MOF methanol sensor and a variety of methanol sensors in literature.

Sensing Material	Operating Temperature (${}^{\circ }$C)	Tested Concentration Range (ppm)	Detection Limit (ppm)	Reference
CuBTC-MOF	25	250–1500	62	[38]
NH${}_{2}$-MIL-53(Al) MOF in Matrimid	28	1000–8000	-	[14]
ZnO hexagonal nanorods	150–250 ${}^{\circ }$C	190–3040	-	[39]
CdS-doped tin oxide	200	0–5000	-	[40]
Copper (II) oxide	350	100–2500	-	[41]
MoS${}_{2}$ nanoflakes	200	200–400	-	[42]
Epoxy acrylate film	room temperature	200–16,000	-	[43]
ZIF-8 MOF	20	500–7000	100	This work

**Table 4:** Table of kinetic parameters and response/recovery time for Methanol.

Temperature (${}^{\circ }$C)	Kinetic Parameters					
	Adsorption		Desorption		Response Time	Recovery Time
	k${}_{1}$	k${}_{2}$	k${}_{3}$	k${}_{4}$	t${}^{+}$ (s)	t${}^{-}$ (s)
20	0.0158	0.0029	0.0141	0.0010	207	981
30	0.0151	0.0021	0.0109	0.0021	198	633
40	0.0141	0.0035	0.0107	0.0020	181	585
50	0.0131	0.0049	0.0108	0.0060	147	298

## Supplementary Materials

The following are available online at https://www.mdpi.com/1424-8220/19/4/888/s1, Figure S1a: SEM image of as-synthesized ZIF-8 at different magnifications, Figure S1b: Measured and simulated XRD plots, Figure S2: Laser profilometer data plot of ZIF-8 MOF coated device, Figure S3: Power consumption in bare device, Figure S4a: Heat transfer mechanisms in the device, Figure S4b: 3D geometry for the FEM model with thickness of the materials, Figure S4c: Micro-hotplate device design, Figure S5: Optical Images of ZIF-8 coated device, Figure S5a: 20 ${}^{\circ }$C, Figure S5b: 50 ${}^{\circ }$C, Figure S5c: 100 ${}^{\circ }$C, Figure S5d: 300 ${}^{\circ }$C, Figure S6a: Capacitive response for increasing methanol concentration, Figure S6b: Capacitive response after baseline drift calibration, Figure S7: Measured baseline capacitance of ZIF-8 coated device in nitrogen at 20 ${}^{\circ }$C, Figure S8a: Capacitive response for 5000ppm of methanol with baseline drift fitting, Figure S8b: Capacitive response after baseline drift calibration, Figure S9: Capacitance of bare device with temperature steps in vacuum, Figure S10a: Capacitance of the bare device with fast temperature steps in vacuum, Figure S10b: Capacitance change ΔC in vacuum of the bare device by fast temperature steps, Figure S10c: Capacitance change Δ*C* during temperature steps from 55 ${}^{\circ }$C to 115 ${}^{\circ }$C, Figure S10d: Δ*C* versus temperature of the bare device in vacuum during fast temperature steps, Figure S11: Comparison Δ*C* versus temperature of the bare device in vacuum for single and fast temperature steps, Figure S12: Fabrication process flowchart MIM capacitor, Figure S13: Capacitance versus frequency of MIM capacitor at different temperatures, Figure S14: Dielectric constant and capacitance of MIM capacitor at different temperatures, Figure S15: FEM Modelling of IDE capacitor, Figure S16: Δ*C* versus temperature of the bare device obtained by FEM model, Figure S17: Comparison of Δ*C* versus temperature of the bare device obtained by the calibration experiments, Figure S18: Capacitive response for exposure to methanol concentration of 5000ppm at (Figure S18a) 30 ${}^{\circ }$C, (Figure S18b) 40 ${}^{\circ }$C, (Figure S18c) 50 ${}^{\circ }$C, Capacitive response after SiN${}_{x}$ isolation layer capacitance calibration (Figure S18d) 30 ${}^{\circ }$C, (Figure S18e) 40 ${}^{\circ }$C, (Figure S18f) 50 ${}^{\circ }$C, Final Capacitive response (Δ*C*) after baseline drift calibration (Figure S18g) 30 ${}^{\circ }$C, (Figure S18h) 40 ${}^{\circ }$C, (Figure S18i) 50 ${}^{\circ }$C, Figure S19: Capacitive response for exposure to water vapor concentration of 5000 ppm at (Figure S19a) 30 ${}^{\circ }$C, (Figure S19b) 40 ${}^{\circ }$C, (Figure S19c) 50 ${}^{\circ }$C, Capacitive response after SiN${}_{x}$ isolation layer capacitance calibration (Figure S19d) 30 ${}^{\circ }$C, (Figure S19e) 40 ${}^{\circ }$C, (Figure S19f) 50 ${}^{\circ }$C, Final Capacitive response (Δ*C*) after baseline drift calibration (Figure S19g) 30 ${}^{\circ }$C, (Figure S19h) 40 ${}^{\circ }$C, (Figure S19i) 50 ${}^{\circ }$C, Figure S20: Stability test with ZIF-8 MOF to Methanol concentration of 5000 ppm, Figure S21: Temperature dependent capacitance response to 5000 ppm methanol measured over three weeks, Figure S22: Time-dependent adsorption kinetic fit for exposure to methanol concentration of 5000 ppm at (Figure S22a) 20 ${}^{\circ }$C, (Figure S22b) 30 ${}^{\circ }$C, (Figure S22c) 40 ${}^{\circ }$C, (Figure S22d) 50 ${}^{\circ }$C, Figure S23: Time-dependent desorption kinetic fit for exposure to methanol concentration of 5000 ppm at (Figure S23a) 20 ${}^{\circ }$C, (Figure S23b) 30 ${}^{\circ }$C, (Figure S23c) 40 ${}^{\circ }$C, (Figure S23d) 50 ${}^{\circ }$C, Table S1: Summary of material parameters for FEM analysis, Table S2: Summary of design parameters for FEM analysis, Table S3: Table of kinetic parameters and regression co-efficient for adsorption curves, Table S4: Table of kinetic parameters and regression co-efficient for desorption curves.
