# Supplementary material for: A Low-Power MEMS IDE Capacitor with Integrated Microhotplate: Application as Methanol Sensor using a Metal-Organic Framework Coating as Affinity Layer
Source: Sensors (Basel). 2019 Feb 20;19(4):888. doi: 10.3390/s19040888 (PMC6412504; doi:10.3390/s19040888)
Supplement: Supplementary file 1 [file sensors-19-00888-s001.zip › MDPI_Sensors_425899_Supplementary.pdf]

Supplementary Materials: A low-power MEMS IDE Capacitor with Integrated Microhotplate: Application as Methanol Sensor using a Metal Organic Framework Coating as Affinity Layer

Manjunath R Venkatesh , Sumit Sachdeva , Brahim El Mansouri , Jia Wei, Andre Bossche, Duco Bosma, Louis C.P.M. de Smet, Ernst J.R. Sudhölter and Guo.Qi. Zhang

1. Structural characterization of ZIF-8 MOF

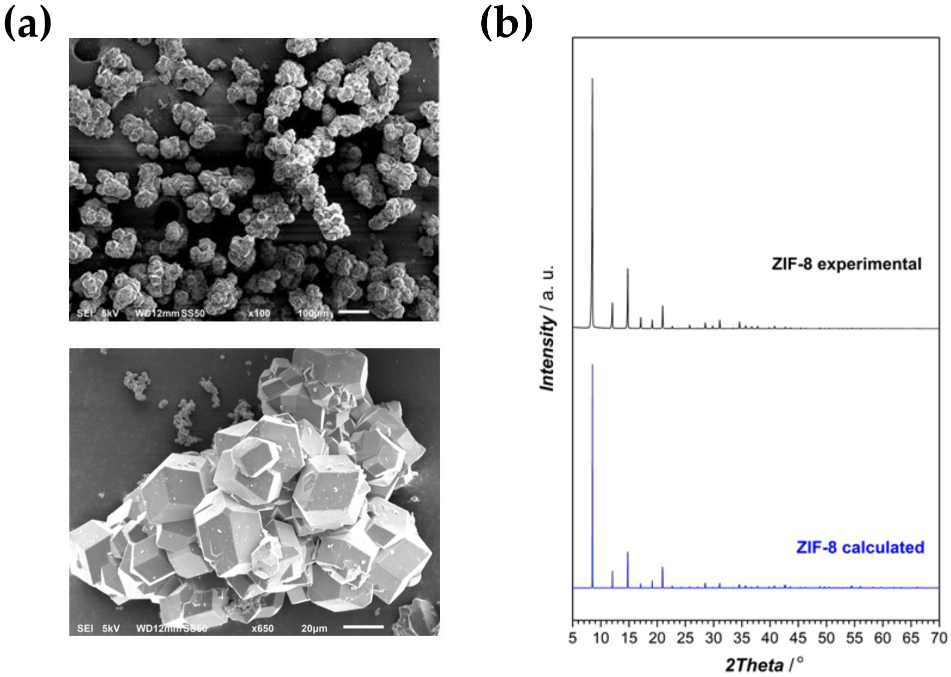

Figure S1. (**a**) SEM image of as-synthesized ZIF-8 at different magnifications. (**b**) Measured and simulated XRD plots.

2. Thickness measurements of ZIF-8 MOF coated device

Keyance Laser profilometer was used and the resulting thickness measurements along the diagonal of the device is shown in Figure S2.

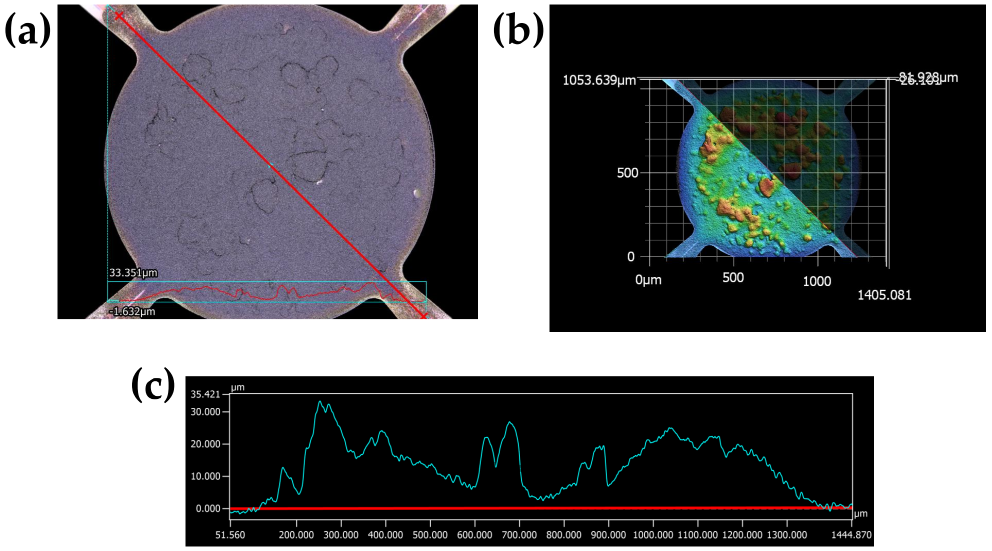

Figure S2. Laser profilometer data plot of ZIF-8 MOF coated device. (**a**) Measured region. (**a**) 3D map of the ZIF-8 MOF coating. (**c**) Layer thickness profile.

3. Thermal characterization of the micro-hotplate and analysis using FEM

3.1. Analysis of the steady-state power consumption

The steady-state power consumption of the bare device in nitrogen (P_{N_2}) and in vacuum (P_{vacuum}) is shown in Figure S3. The heat loss by different means of heat transfer of the bare device is estimated as following[1][2],

- The radiative power loss is theoretically calculated for the top and bottom area of the device using Stefan-Boltzmann law given by,

$$P_{rad}(T_h) = 2 * \epsilon\sigma A_h((T_h^4 - T_0^4)) \tag{1}$$

where ϵ is the effective emissivity of the microhotplate taken as 1[1], σ is the Stefan-Boltzmann constant given as 56.7$\cdot$ 10^{-9} $Wm^{-2}K^{-2}$, A_h is the area of the microhotplate calculated to be $5.25{\cdot}10^{-7}$ m^2 , T_h is the average temperature of the heater at a given input power and T_0 is the ambient temperature. The calculated radiative heat loss $P_{rad}(T_h)$ is shown in Figure S3. At a temperature of 200°C, $P_{rad}(T_h)$ is 2.5mW.

- The conductive power loss through the membrane and beams of the microhotplate is determined by subtracting the radiative power loss from the power consumption of the bare device in vacuum given by,

$$P_{cond}(T_h) = P_{vacuum}(T_h) - P_{rad}(T_h) \tag{2}$$

At a temperature of 200°C the obtained conductive power loss P_{cond}(T) is 6.8mW.

- The convective power loss $P_{conv}(T_h)$ (Figure S3) due to nitrogen flow is obtained by subtracting the power consumption of the device in nitrogen with power consumption in vacuum given by ,

$$P_{conv}(T_h) = P_{N_2}(T_h) - P_{vacuum}(T_h) \tag{3}$$

The obtained convective power loss $P_{conv}(T_h)$ at 200°C is 16.5mW. Considering the convective power $P_{conv}(T_h)$, the convection coefficient $h_{conv}(T_h)$ is extracted using the following equation[1],

$$h_{conv}(T_h) = \frac{P_{conv}(T_h)}{A_h(T_h - T_0)} \tag{4}$$

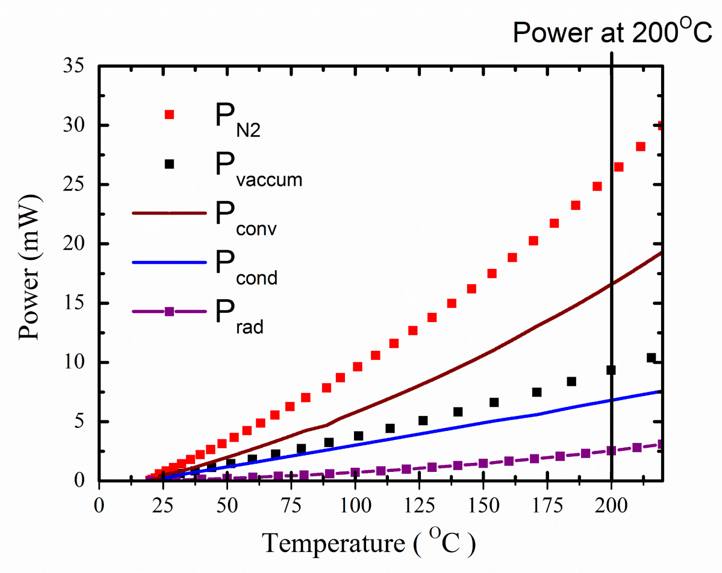

Figure S3. Power consumption in bare device.

For the bare device an input power of 3.5mW and 25.8mW is required to obtain a temperature of 50°C and 200°C in nitrogen respectively. The obtained power loss due to convection is 64%, 26% due to conduction and 10% is due to radiation. Hence, heat loss due to convection is the significant component that inhibits low power operation for a long duration.

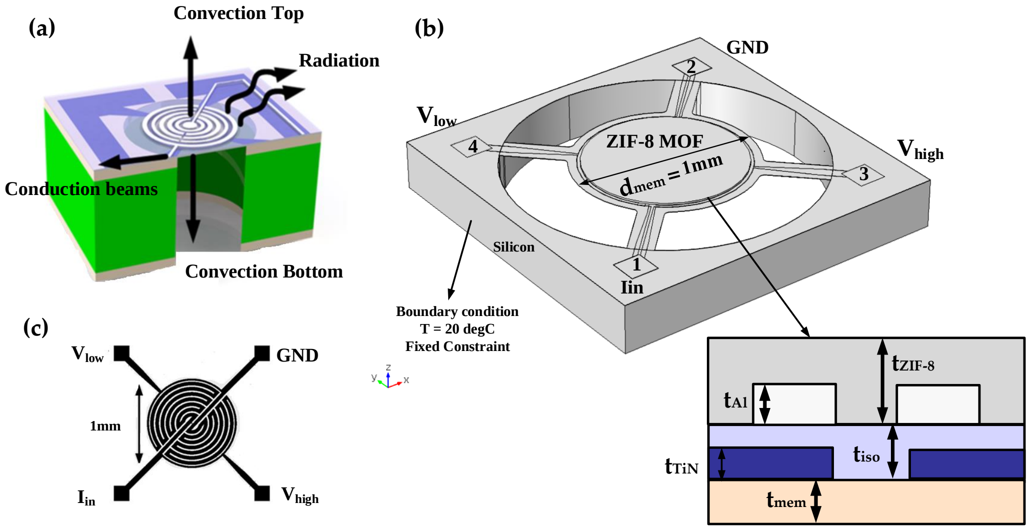

Figure S4. Thermal analysis (**a**) Heat transfer mechanisms in the device. (**b**) 3D geometry for the FEM model with thickness of the materials. (**c**) Micro-hotplate device design.

3.2. Thermal Analysis using FEM

The heat generated in the micro-hotplate is transferred by three methods namely: 1) conduction through the beams of the device, 2) convection through the top and bottom of the device and 3) radiation as shown in Figure S4a.

A 3D geometry of the device is developed in COMSOL Multiphysics® 5.3 (Figure S4b). In this 3D model, the electro-thermal analysis is performed by coupling the electrical domain to the thermal domain. The conjugate heat transfer physics is selected for the entire domain, whereas the electric current physics module is only selected for the TiN micro-hotplate with boundary conditions for current input at terminal 1(I_{in}) and ground terminal 2 (GND). The voltage output is measured at terminal 3 (V_{high}) and terminal 4 (V_{low}) enabling a four-probe IV measurement configuration.

The heat generated due to joule heating is proportional to the current density given by [3][4],

$$Q \propto |J|^2 \tag{5}$$

$$Q = \frac{|J|^2}{\sigma(T)} \tag{6}$$

where σ(T) is the electrical conductivity, T is the temperature and J is the current density. The electrical conductivity is a function of temperature given by,

$$\sigma(T) = \frac{1}{\rho_0(1 + \alpha(T - T_0))} \tag{7}$$

where ρ_0 is the electrical conductivity at room temperature (T_0) and α is the temperature co-efficient of resistance (TCR). The value of TCR of the device used for measurement is α = $0.000725°C^{-1}$.

The current density is proportional to the electric field E, which is the negative gradient of potential V. Using this in Equation 3.2, we get,

$$Q = \frac{|J|^2}{\sigma(T)} = \frac{|\sigma(T)E|^2}{\sigma(T)} = \sigma(T)|\nabla V|^2 \tag{8}$$

The governing equations for the steady-state heat conduction for joule heating process are described below[5],

$$\nabla \cdot (k(T)\nabla T) + \frac{(J^2)}{\sigma(T)} = 0 \tag{9}$$

where *k(T)* is the respective thermal conductivity of silicon-nitride membrane, isolation layer, TiN and aluminum electrodes. The material parameters for the FEM model is described in Table S1.

Table S1. Summary of material parameters for FEM analysis.

Material	Thermal Conductivity (k) W/mK	Density Kg/m3	Heat Capacity (Cp) J/kgK
LPCVD silicon nitride[6]	10-13	3200	700
PECVD silicon nitride[6]	2-4.5	2800	700
Silicon[6]	157	2320	700
TiN[6]	19.2	5220	600
Al[6]	236	2700	900
ZIF-8[7]	0.326	950	700

The generated heat transferred through the beams of the micro-hotplate is computed based on the thermal conductivity of the layers. The radiative heat flux is defined on the entire surface with an effective emissivity (ϵ) of silicon nitride layers equal to 1 [5]. The convective heat transfer coefficient

is defined on the entire device with $h_{conv}(T)$ determined from the measured results. Using these conditions and design parameters shown in Table S1, the thermal modeling of the device is done. In the 3D model, the boundary condition for the silicon substrate is fixed at a temperature of 20°C. The current (I_{in}) is swept from 100μA to 5mA for the thermal analysis of the device in vacuum and up to 10mA for the device under convective conditions.

Table S2. Summary of design parameters for FEM analysis.

Design Parameter	Value	Unit
Thickness of silicon nitride (t_{mem})	500	nm
Diameter of membrane (d_{mem})	1	mm
Thickness of silicon nitride isolation layer (t_{SiNx})	1000	nm
Thickness of TiN electrodes (t_{TiN})	400	nm
Thickness of Al electrodes (t_{Al})	1000	nm
Thickness of ZIF-8 MOF (t_{ZIF-8})	10	μm

4. Optical imaging

The surface morphology of ZIF-8 MOF at different magnification at 20°C, 50°C, 100°C and 300°C is shown in Figure S5a, S5b, S5c and S5d respectively.

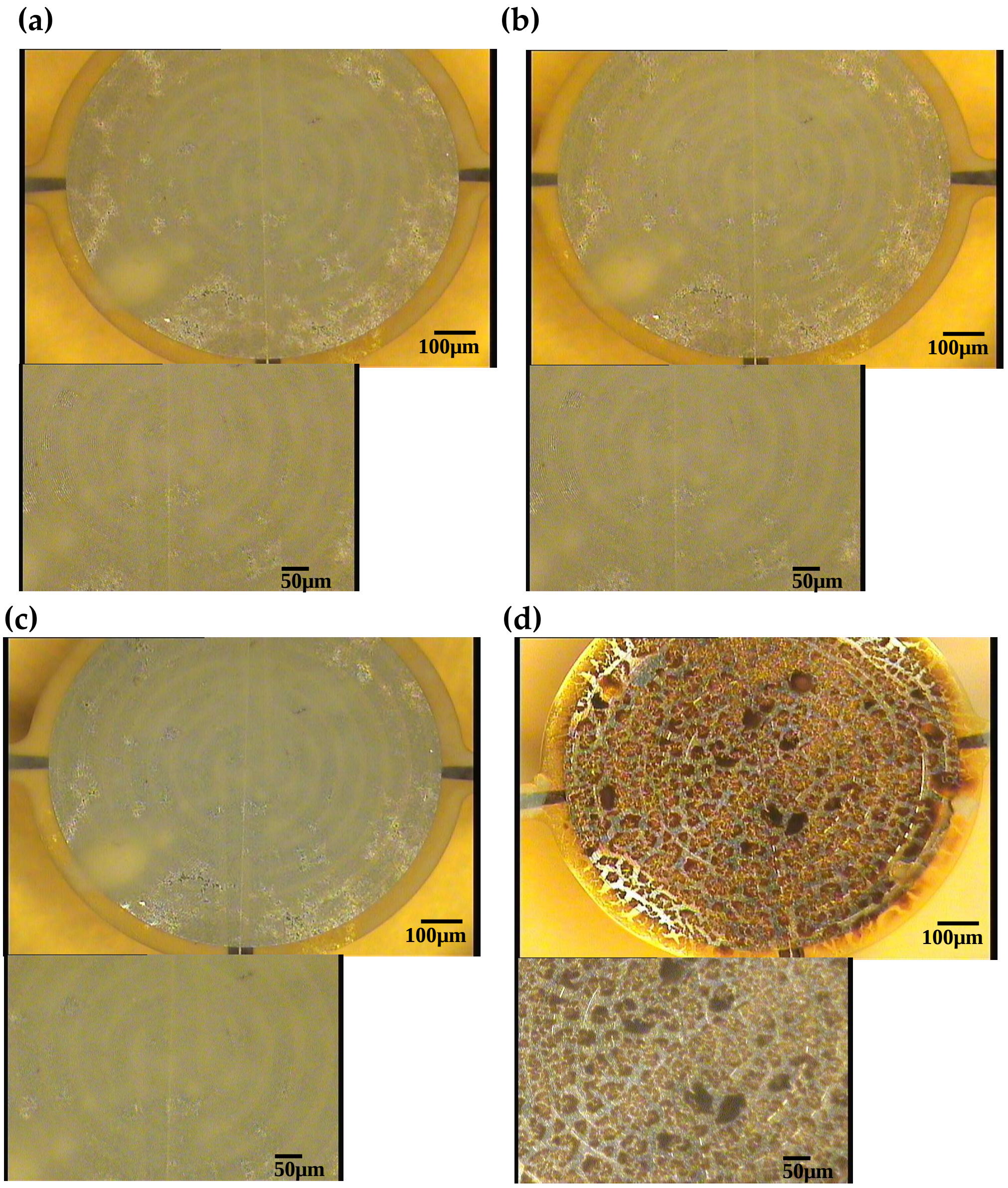

Figure S5. Optical Images of ZIF-8 coated device (a) 20°C (b) 50°C (c) 100°C (d) 300°C.

5. Methanol sensing study

The sensing measurements for increasing methanol concentration from 500ppm to 7000ppm with baseline drift calibration line is shown in Figure S6a. After the baseline drift calibration is done, the data for sensing response towards methanol is shown in Figure S6b.

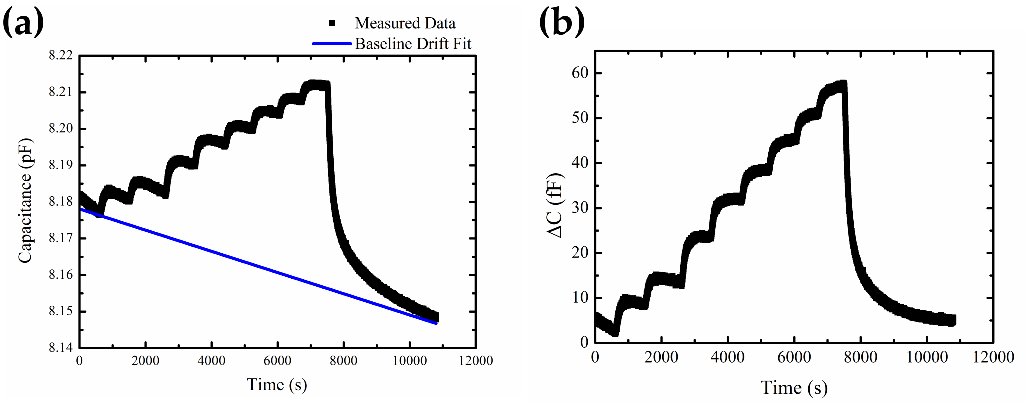

Figure S6. (**a**) Capacitive response for increasing methanol concentration. (**b**) Capacitive response after baseline drift calibration.

The baseline capacitance measurement for ZIF-8 coated device in nitrogen is shown in S7.

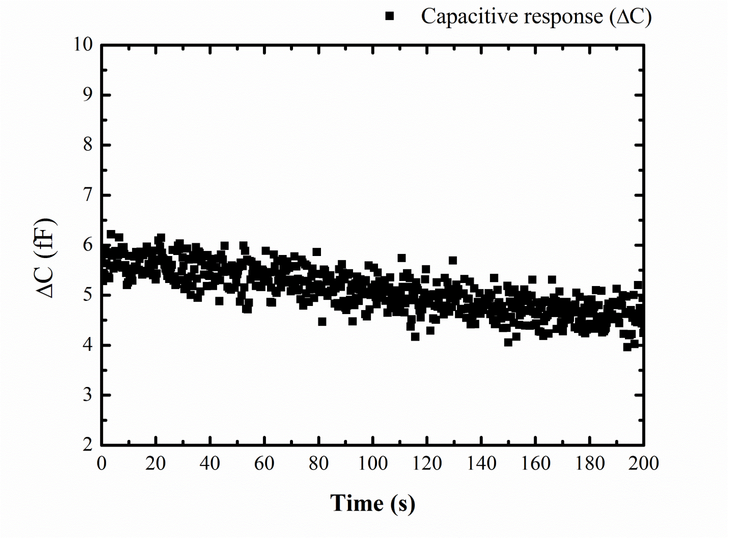

Figure S7. Measured baseline capacitance of ZIF-8 coated device in nitrogen at 20°C.

The reversibility study for exposure to methanol concentration of 5000ppm with baseline drift calibration line is shown in Figure S8a. The response after the baseline drift calibration is shown in Figure S8b.

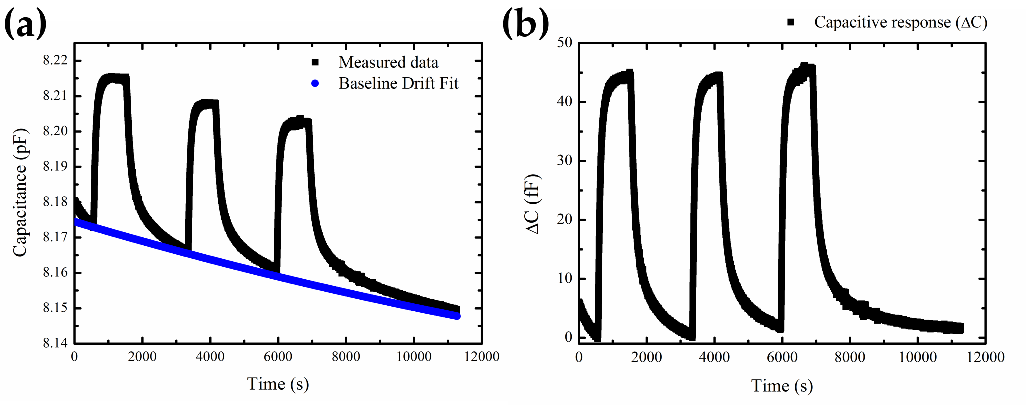

Figure S8. (**a**) Capacitive response for 5000ppm of methanol with baseline drift fitting. (**b**) Capacitive response after baseline drift calibration.

6. Temperature-dependent capacitance characterization of the bare device

In this section, the changes in the capacitance of the bare device with temperature due to the SiN_x isolation layer is characterized.

Before the measurement of the capacitance of the bare device, two preconditioning steps in vacuum were performed. In the first step, the entire gas chamber with the MEMS device was heated in an oven at 100°C for 1hr and cooled to room temperature. In the second step, the operating temperature of the device was increased to 200°C for 30 minutes using the microhotplate and cooled to ambient temperature of 20°C for one hour. These steps were done to remove moisture content that could be present on top of the IDEs while placing the device in the chamber. The capacitance was measured at a constant oscillation voltage of 1V, and at a frequency of 10kHz. The measured capacitance of the bare device after the preconditioning steps was 6.969 pF.

Next, a sequence of temperature steps was programmed using the Keithley 2611B by changing the current through the microhotplate, and the capacitance measured simultaneously as shown in Figure S9. From the measurement data, it is evident that during the temperature steps, there is a change in the capacitance of the bare device. The changes in the capacitance for the temperature steps to 55°C and 120°C is a combination of two behaviors, (i) a sharp increase in ΔC could be due to the change in dielectric constant (ϵ_r) of the PECVD SiN_x isolation layer with increasing temperature, (ii) a slow decay in the capacitance of the device caused by the desorption of residual moisture adsorbed on the top of the IDEs from the chamber surrounding. This can be due to the fact that the vacuum could not be high enough to remove the moisture present in the chamber. For the next temperature steps to 165°C and 220°C, the measured change in capacitance (ΔC) increases and remains almost constant during the temperature cycle. At these high temperatures, the water molecules are not adsorbed on the IDEs, and thus the slow decay is not observed.

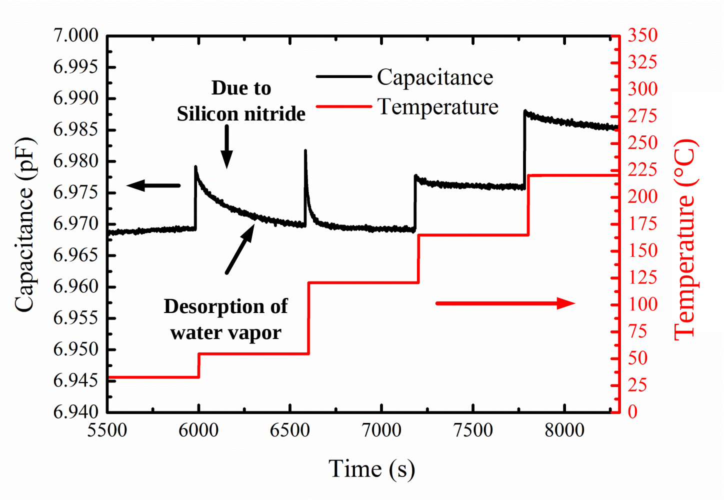

Figure S9. Capacitance of bare device with temperature steps in vacuum.

In order to accurately determine the observed changes in the bare device capacitance with temperature (behavior (i) and (ii)), the following two experiments were performed,

1. The sharp increase in capacitance (behavior (i)) and the slow decay (behavior (ii)) can be decoupled by changing the temperature of the microhotplate between two temperature points rapidly. In this experiment, the current through the microhotplate is changed per second to obtain two temperature points (fast temperature steps). The capacitance is measured simultaneously at a frequency of 10kHz and oscillation voltage of 1V. The measured capacitance for the fast temperature steps is described in Figure S10a. The temperature of the microhotplate is changed between 30°C to 55°C, 55°C to 115°C, 115°C to 160°C and 160°C to 215°C. Figure S10c shows the temperature steps between 55°C to 115°C with the capacitance of the bare device changing from 6.956pF to 6.968pF. The difference in capacitance ΔC of the bare device for the corresponding temperature steps is shown in Figure S10b. The resulting average changes in capacitance ΔC for the fast temperature steps is shown in Figure S10d. It is seen that upon increasing the temperature from 30°C to 220°C, the ΔC of the bare device increases linearly. This change in capacitance is due to the change in the dielectric constant of PECVD SiN_x layer, which needs to be considered as a calibration factor during temperature cycle measurements. It is seen that even during the fast temperature steps, there is an inherent change in the capacitance of the bare device. The resulting change in the bare device capacitance (ΔC) obtained from the single temperature step and fast temperature steps is shown in Figure S11. The change in capacitance ΔC is found to be linear with increasing temperatures. The ΔC obtained from the linear fit at a temperature of 50°C is 7.5fF, and at 200°C it is 38 fF.

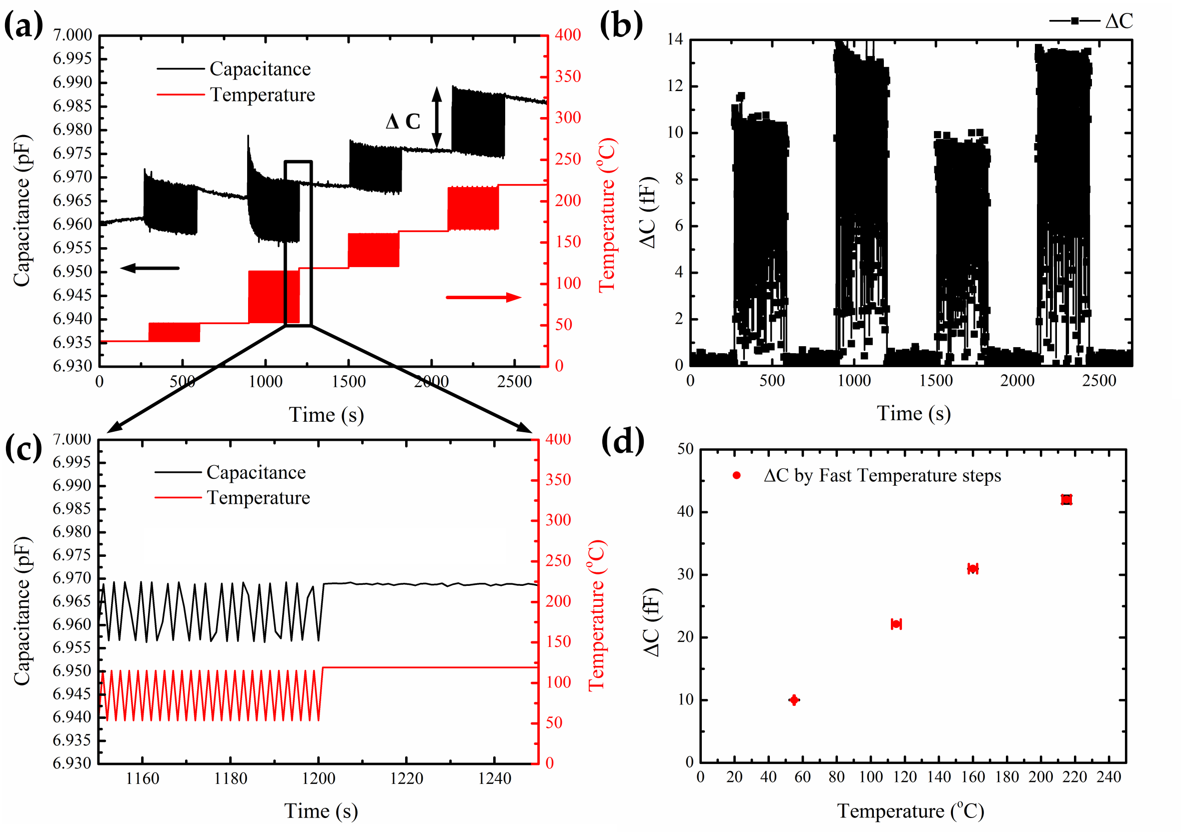

Figure S10. (**a**) Capacitance of the bare device with fast temperature steps in vacuum. (**b**) Capacitance change ΔC in vacuum of the bare device by fast temperature steps. (**c**) Capacitance change ΔC during temperature steps from 55°C to 115°C. (**d**) ΔC versus temperature of the bare device in vacuum during fast temperature steps.

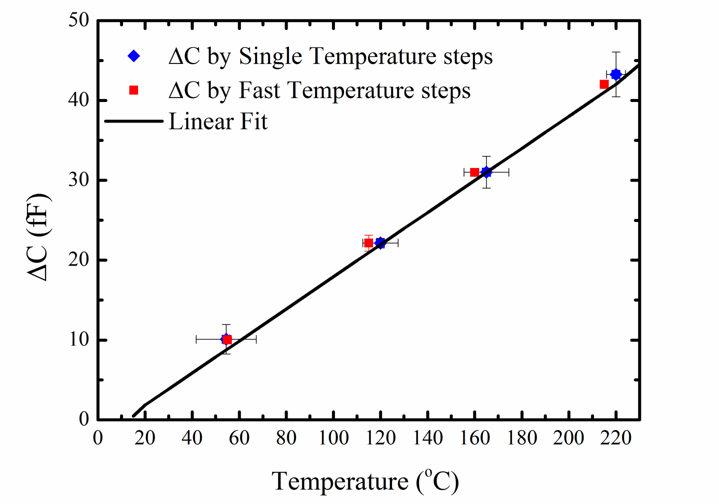

Figure S11. Comparison ΔC versus temperature of the bare device in vaccum for single and fast temperature steps.

2. The change in capacitance purely due to the dielectric constant variation (behavior (i)) can be determined by characterizing a metal-insulator-metal (MIM) capacitor structure. In this experiment, a MIM capacitor with 200nm PECVD SiN_x as the dielectric layer is fabricated and characterized. The fabrication flowchart of the MIM capacitor with PECVD silicon nitride as the dielectric layer is shown in Figure S12. A standard 4-inch p-type silicon wafer was used as the substrate for the device. A metal layer of 1μm aluminum was deposited using a Trikon Sigma sputter coater. A layer of 200nm thick PECVD silicon nitride was deposited using Novellus PECVD equipment. The contact opening mask layer was exposed to etch silicon nitride to obtain the contact to the bottom aluminum layer. For the top electrode, an aluminum metal layer of 1.4μm was deposited on top of the PECVD silicon nitride.

   The capacitance of the MIM capacitor with PECVD SiN_x is given by,

$$C_{SiN_x} = \epsilon_0 \cdot \epsilon_r \cdot \frac{A_{cap}}{d_{cap}} \tag{10}$$

   where, ϵ_0 is the permittivity of free space, ϵ_r is the dielectric constant of PECVD SiN_x, A_{cap} is the area of the MIM capacitor and d_{cap} is the thickness of the SiN_x layer. The temperature steps were done using the thermochuck temperature controller from 20°C to 200°C. The capacitance was measured as a function of frequency from 1kHz to 100kHz for different temperatures as shown in Figure S13. The measured capacitance and the extracted dielectric constant for different temperatures at a frequency of 10kHz and oscillation voltage of 1V is shown in Figure S14. The capacitance of the MIM capacitor increases with increasing temperatures due to the increase in the dielectric constant of the PECVD SiN_x layer. The measured capacitance C_{SiN_x} is 227.78 pF at 20°C and C_{SiN_x} = 230.12 pF at 200°C. The extracted dielectric constant (ϵ_r) increases from 6.554 at 20°C to 6.621 at 200°C.

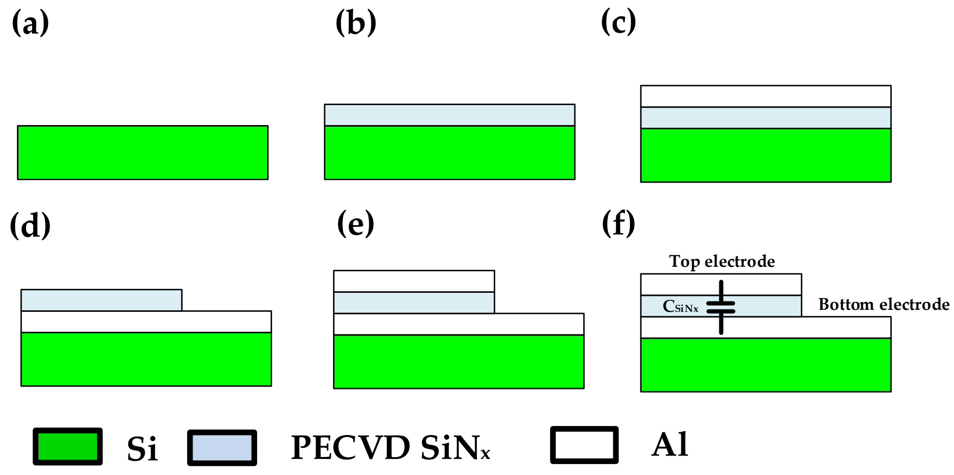

Figure S12. Fabrication process flowchart MIM capacitor (**a**) Silicon substrate. (**b**) 1μm Al 1%Si for bottom metal. (**c**) 200nm PECVD Silicon nitride. (**d**) Bottom electrode contact opening and silicon nitride etching. (**e**) 1μm Al/1%Si for top metal. (**f**) Schematic representation of MIM capacitor C_{SiN_x}.

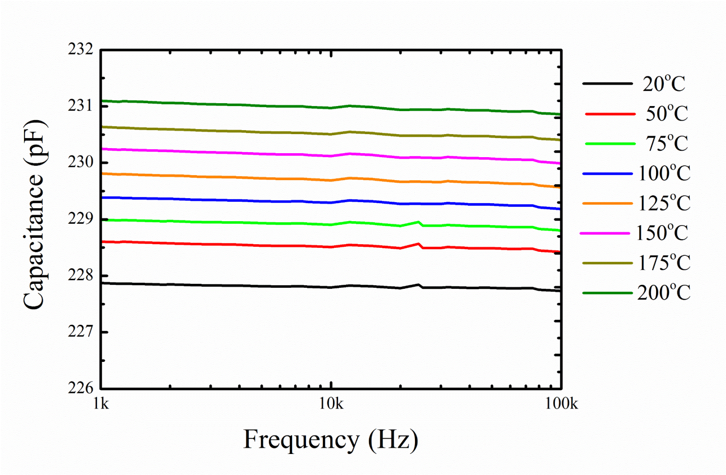

Figure S13. Capacitance versus frequency of MIM capacitor at different temperatures.

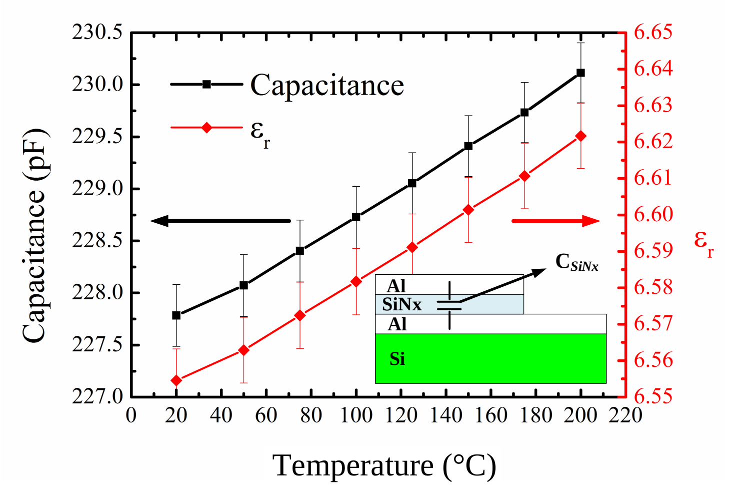

Figure S14. Dielectric constant and capacitance of MIM capacitor at different temperatures.

6.1. FEM analysis of IDE capacitor

In this section, the increase in capacitance due to the temperature-dependent dielectric constant of the SiN_x isolation layer is validated by developing a 2D FEM model of the IDE capacitor.

The obtained relation of ϵ_r vs temperature for the SiN_x isolation layer is used to determine the total capacitance of the device and contribution of the shielded and unshielded electrodes by using a 2D FEM model. The total capacitance between the electrodes depends on the dielectric constants of the substrate and dielectric constant of the layer above the electrodes. Due to the design of the microhotplate electrodes, there are regions where the IDEs are not adequately shielded by the underlying microhotplate electrodes. For a microhotplate designed within a membrane having a diameter of 1mm, about 67% of the area underneath the IDE is shielded by the microhotplate pattern whereas 33% of the area under the IDEs is not shielded. Thus, the total capacitance is obtained by the sum of two capacitances: shielded IDEs ($C_{shielded}$) and unshielded IDEs ($C_{unshielded}$).

Figure S15b illustrates a local cross-section of the IDE and heater structure, together with a corresponding circuit model (only capacitive components are shown.). The total capacitance is given by,

$$C_{total} = C_{shielded} + C_{unshielded} \tag{11}$$

where,

$$C_{shielded} \text{ and } C_{unshielded} = C_{IDE} + C_{parasitic} \tag{12}$$

The parasitic capacitance contributing to the shielded IDEs is mainly due to the isolation layer capacitance (C_{1s}). However, the parasitic capacitances for the unshielded IDEs are due to the isolation layer capacitance (C_1), membrane layer capacitance (C_2), and capacitance through the space underneath the membrane (C_3) as described in Figure S15a.

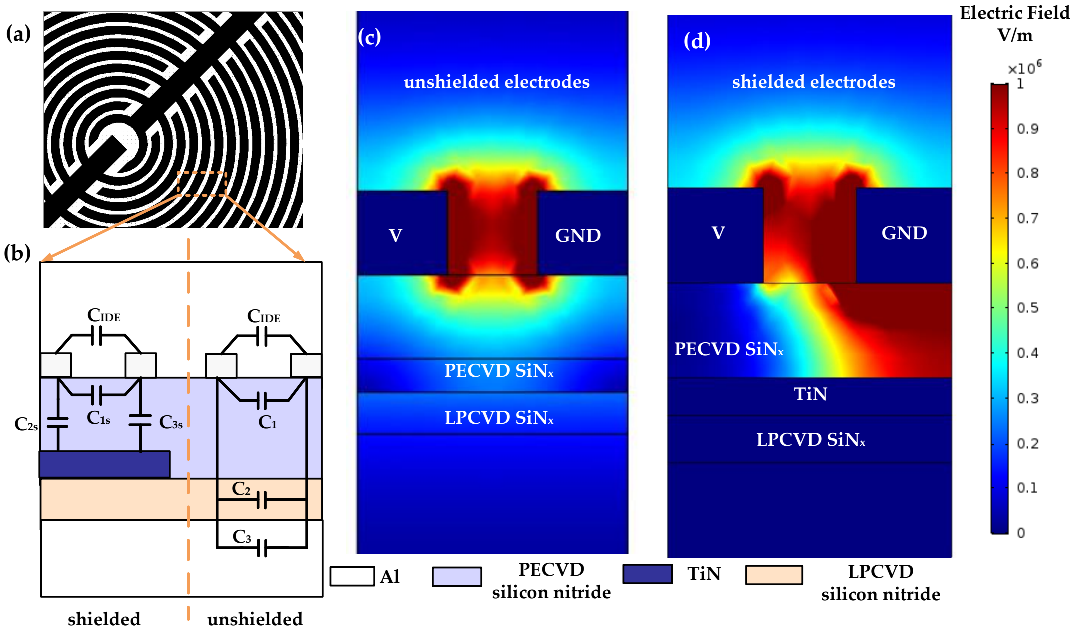

Figure S15. FEM Modelling of IDE capacitor. (**a**) Mask layout of a circular IDE. (**b**) Cross-section view of IDE with microhotplate electrodes. (**c**) Electric field distribution of unshielded electrodes. (**b**) Electric field distribution of shielded electrodes.

A 2D FEM model is developed by considering the half spacial wavelength for aluminum IDE pair with SiN_x membrane, TiN electrode, SiN_x isolation layer (Figure S15c and S15d)[8][9]. One electrode terminal is defined with potential V_0 = 1V and second electrode terminal is defined as ground. The other sides of the 2D model are at a boundary condition of zero charge. The electric field for the FEM is computed by solving Gauss's law with electric potential V_0 as the independent variable[9]. The capacitance of the IDEs in the FEM model is calculated by computing the terminal charges at the electrodes given by,

$$C_{total} = N \cdot \frac{Q}{V_0} \tag{13}$$

where, N is the number of electrode pairs. The IDE dimensions based on the design parameters are W = 2μm, G = 1μm and the number of electrodes (N) is 164. The thickness of the isolation layer is 1μm and thickness of aluminum electrodes is 1μm. The device capacitance is simulated at a frequency of 10kHz. The capacitance C_{total} is obtained as a function of the dielectric constant of the silicon nitride layer and the change in capacitance (ΔC) for the temperature steps is calculated. The resulting change in capacitance (ΔC) of the IDE capacitor is found to be increasing linearly with temperature as shown in Figure S16.

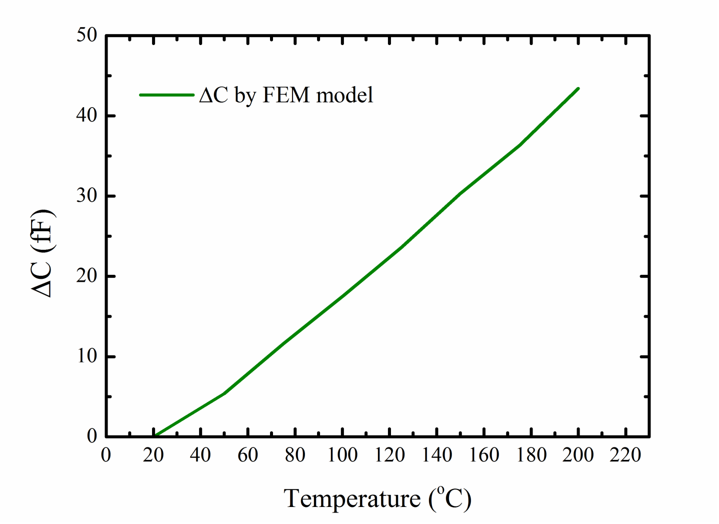

Figure S16. ΔC versus temperature of the bare device obtained by FEM model.

6.2. Capacitance Calibration

The linear change in the capacitance of the bare device (ΔC) with increasing temperatures as described in Figure S17, is found to be mainly due to the change in the dielectric constant of PECVD SiN_x isolation layer with temperature, and it is validated by the above two experiments. At a temperature of 200°C, the difference between FEM data and experimental calibration of ΔC is 12% for single temperature steps, and 17% for fast temperature steps (Figure S17). The length of the electrode, the area of the shielded and unshielded electrode pair need to be more accurately determined for the 2D model. Thus, the FEM model can be used for flexible designs of the IDE capacitor integrated with microhotplate. The linear fit derived in this section is used as the calibration factor for temperature-dependent capacitance sensing study in Section 3.5 in the main manuscript.

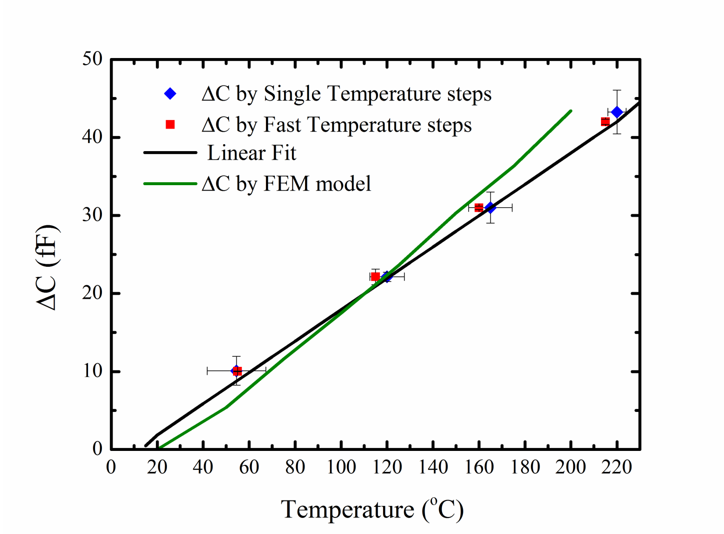

Figure S17. Comparison of ΔC versus temperature of the bare device obtained by the calibration experiments.

7. Temperature-dependent methanol and water vapor sensing response

The measured response has two calibration steps for the data analysis of temperature-dependent sensing response. First, the measured data is calibrated with temperature-dependent isolation layer capacitance (ΔC_{SiN_x}). In the second step, the baseline drift is calibrated. The data analysis for the capacitance sensing response to exposure of methanol and water vapor at a constant concentration of 5000ppm for different temperatures is shown in Figure S18a - S18i and Figure S19a - S19i, respectively.

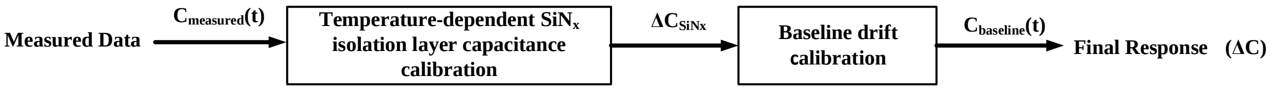

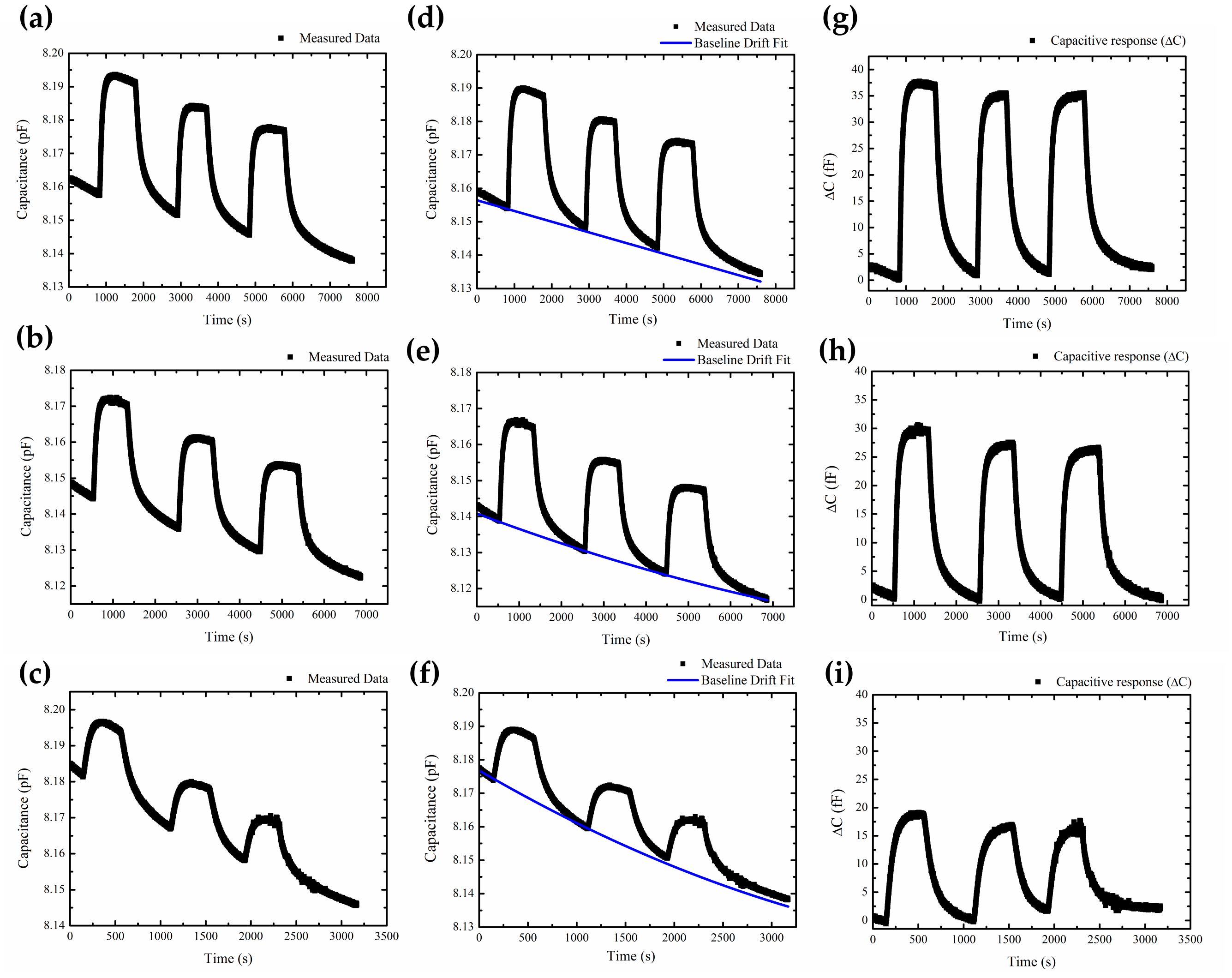

Figure S18. Capacitive response for exposure to methanol concentration of 5000ppm at (**a**) 30°C (**b**) 40°C (**c**) 50°C. Capacitive response after SiN_x isolation layer capacitance calibration (**d**) 30°C (**e**) 40°C (**f**) 50°C. Final Capacitive response (Δ C) after baseline drift calibration (**g**) 30°C (**h**) 40°C (**i**) 50°C.

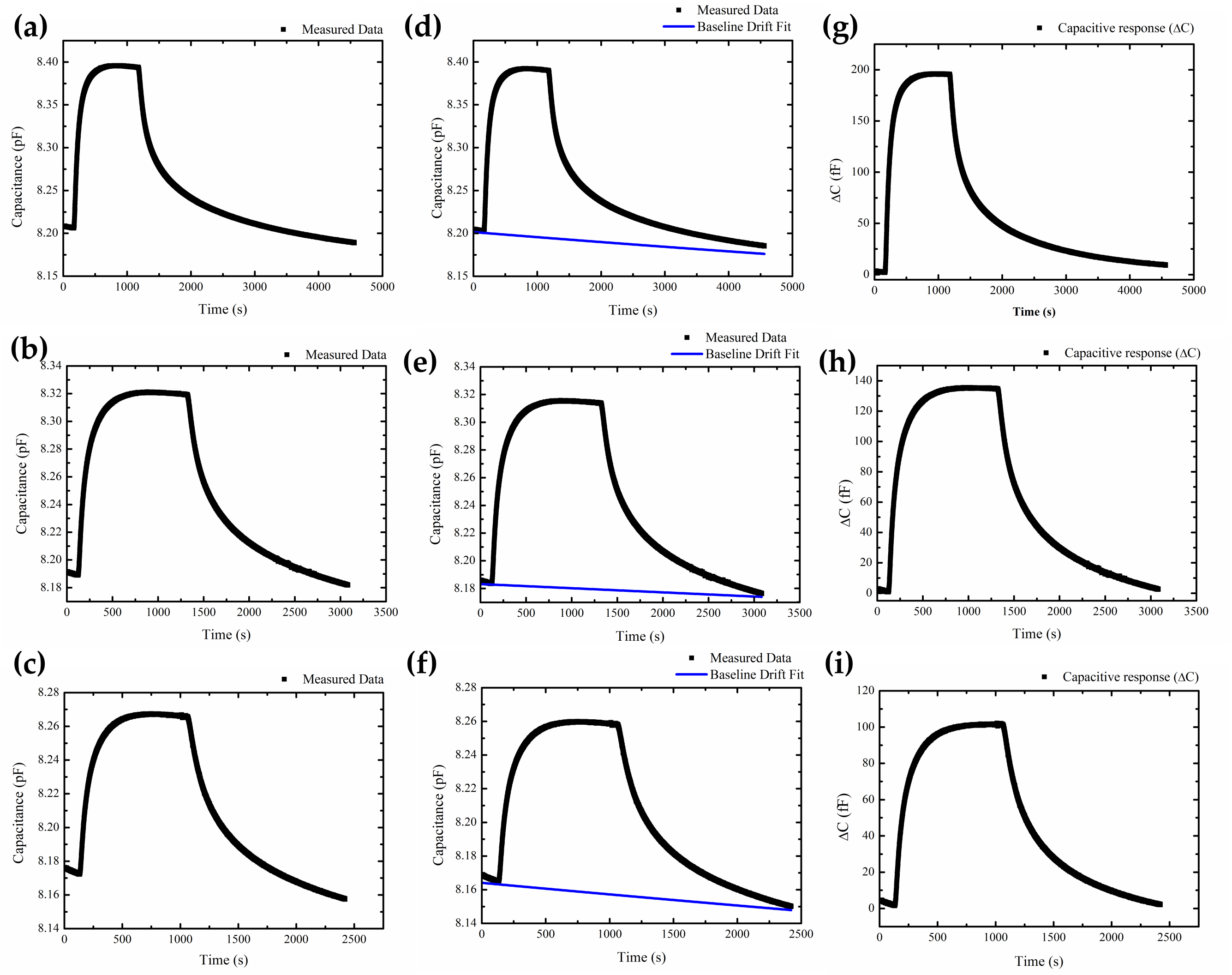

Figure S19. Capacitive response for exposure to water vapor concentration of 5000ppm at (**a**) 30°C (**b**) 40°C (**c**) 50°C. Capacitive response after SiN_x isolation layer capacitance calibration (**d**) 30°C (**e**) 40°C (**f**) 50°C. Final Capacitive response (Δ C) after baseline drift calibration (**g**) 30°C (**h**) 40°C (**i**) 50°C.

8. Stability test

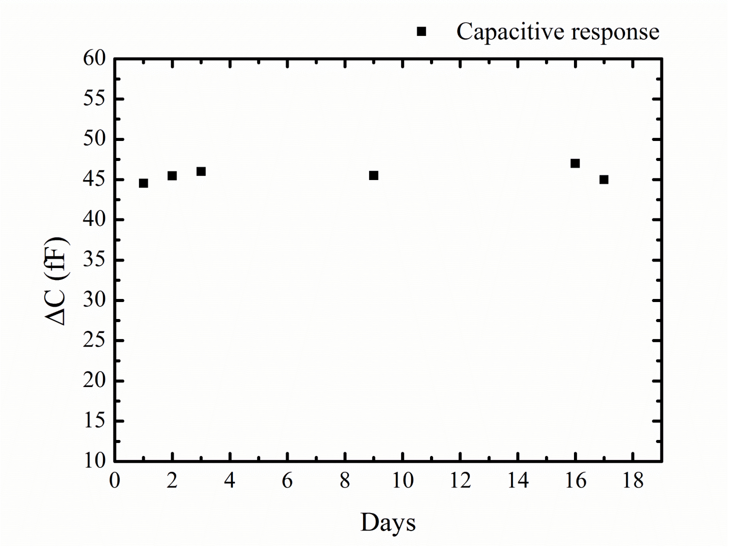

Figure S20. Stability test with ZIF-8 MOF to Methanol concentration of 5000ppm.

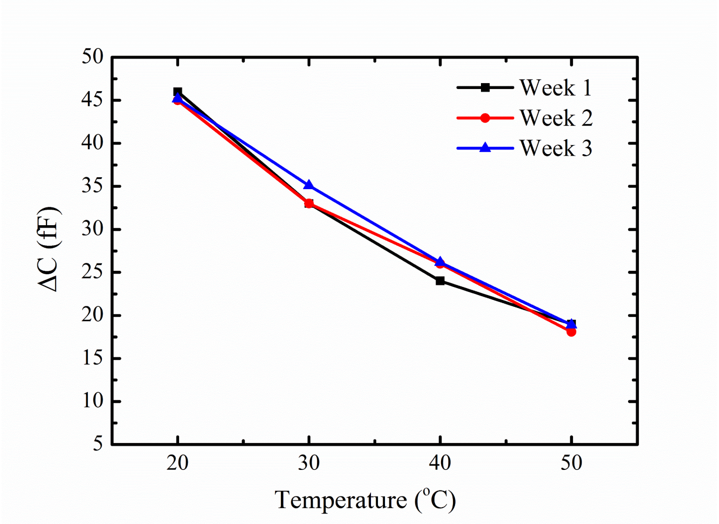

Figure S21. Temperature dependent capacitance response to 5000ppm methanol measured over three weeks.

9. Temperature-dependent adsorption and desorption kinetic modelling

In this section, the time-dependent kinetics of methanol fitted using double exponential model at different temperatures is described. The curve fit is obtained using Equation 14 for the adsorption process and described in Figures S22a - d. Equation 15 is used for the curve fit of the desorption process and shown in Figures S23a - d. The kinetic parameters extracted for the adsorption and desorption curves with corresponding regression co-coefficients is shown in Table S3 and Table S4 respectively.

$$\frac{\Delta C}{C_{max}} = A_{1a}(1 - e^{-k_1}) + A_{2a}(1 - e^{-k_2}) \tag{14}$$

$$\frac{\Delta C}{C_{max}} = A_{1d}(e^{-k_3}) + A_{2d}(e^{-k_4}) \tag{15}$$

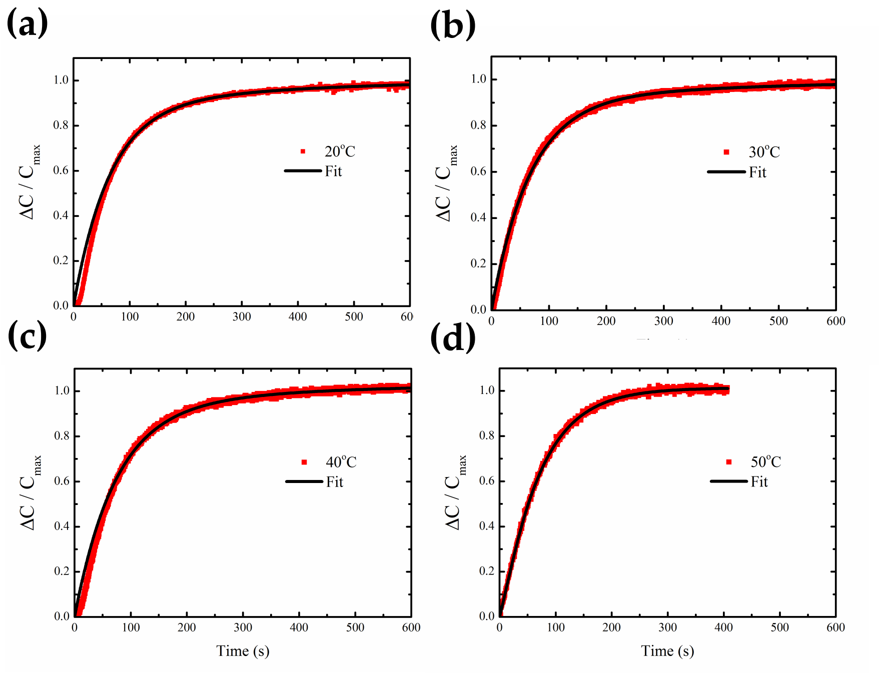

Figure S22. Time-dependent adsorption kinetic fit for exposure to methanol concentration of 5000ppm at (**a**) 20°C (**b**) 30°C (**c**) 40°C (**d**) 50°C.

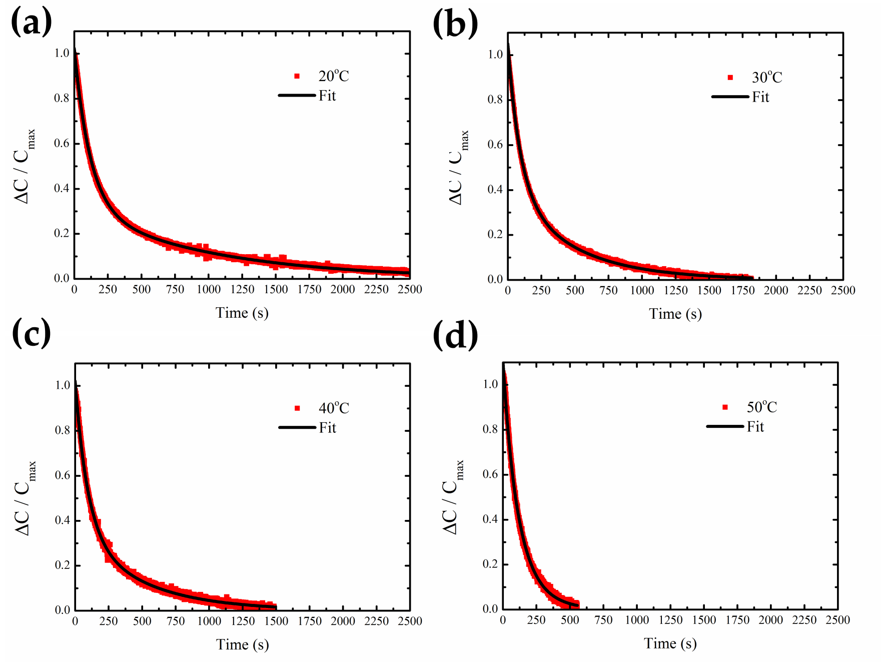

Figure S23. Time-dependent desorption kinetic fit for exposure to methanol concentration of 5000ppm at (**a**) 20°C(**b**) 30°C (**c**) 40°C (**d**) 50°C.

Table S3. Table of kinetic parameters and regression co-efficient for adsorption curves.

Temperature	k_1	k_2	R^2
20	0.0158	0.0029	99.95
30	0.0151	0.0021	99.81
40	0.0141	0.0035	99.86
50	0.0131	0.0049	99.89

Table S4. Table of kinetic parameters and regression co-efficient for desorption curves.

Temperature	k_3	k_4	R^2
20	0.0141	0.0010	99.86
30	0.0109	0.0021	99.89
40	0.0107	0.0020	99.85
50	0.0108	0.0060	99.80

References

1. Silvestri, C.; Riccio, M.; Poelma, R.H.; Morana, B.; Vollebregt, S.; Santagata, F.; Irace, A.; Zhang, G.Q.; Sarro, P.M. Thermal characterization of carbon nanotube foam using MEMS microhotplates and thermographic analysis. *Nanoscale* **2016**, *8*, 8266–8275.
2. Pike, A.; Gardner, J.W. Thermal modelling and characterisation of micropower chemoresistive silicon sensors. *Sensors and Actuators B: Chemical* **1997**, *45*, 19–26.
3. Example—Resistive Heating, COMSOL Multiphysics. https://www.comsol.com/forum/thread/attachment/65707/resistive_heating_sbs-10665.pdf.
4. Heating Circuit, COMSOL Multiphysics. https://www.comsol.com/model/download/468651/models.sme.heating_circuit.pdf.
5. Silvestri, C.; Picciafoco, P.; Morana, B.; Santagata, F.; Zhang, G.; Sarro, P. Electro-thermal simulation and characterization of vertically aligned CNTs directly grown on a suspended microhoplate for thermal management applications. *IEEE Sensors*, 2014, pp. 827–830.
6. Madou, M. *Fundamentals of Microfabrication: The Science of Miniaturization, Second Edition*; Taylor & Francis, 2002.
7. Cravillon, J.; Nayuk, R.; Springer, S.; Feldhoff, A.; Huber, K.; Wiebcke, M. Controlling zeolitic imidazolate framework nano-and microcrystal formation: insight into crystal growth by time-resolved in situ static light scattering. *Chemistry of Materials* **2011**, *23*, 2130–2141.
8. Mamishev, A.V.; Sundara-Rajan, K.; Yang, F.; Du, Y.; Zahn, M. Interdigital sensors and transducers. *Proceedings of the IEEE* **2004**, *92*, 808–845.
9. Oberländer, J.; Jildeh, Z.B.; Kirchner, P.; Wendeler, L.; Bromm, A.; Iken, H.; Wagner, P.; Keusgen, M.; Schöning, M.J. Study of interdigitated electrode arrays using experiments and finite element models for the evaluation of sterilization processes. *Sensors* **2015**, *15*, 26115–26127.
