# Supplementary figures and images for: A Low-Power MEMS IDE Capacitor with Integrated Microhotplate: Application as Methanol Sensor using a Metal-Organic Framework Coating as Affinity Layer
Source: Sensors (Basel). 2019 Feb 20;19(4):888. doi: 10.3390/s19040888 (PMC6412504; doi:10.3390/s19040888)

(a)

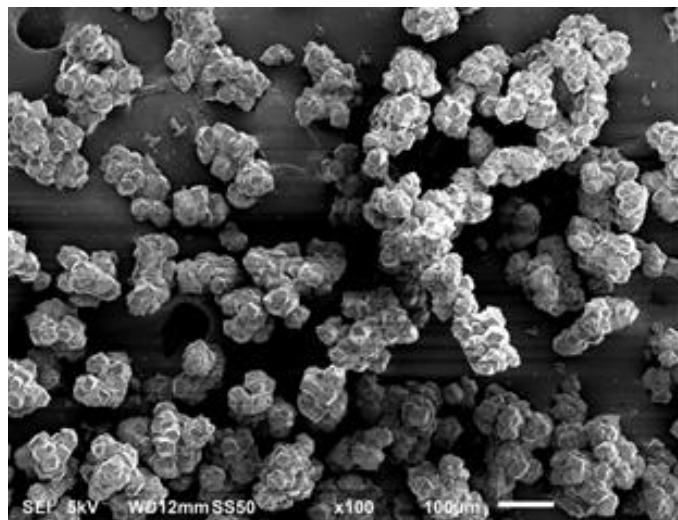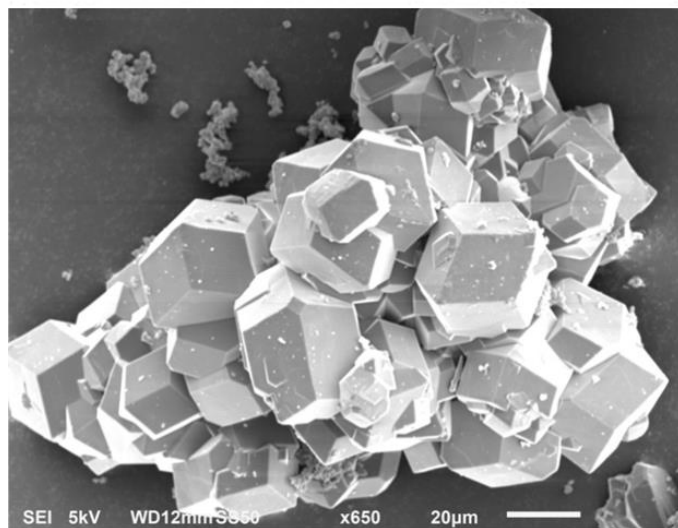

(b)

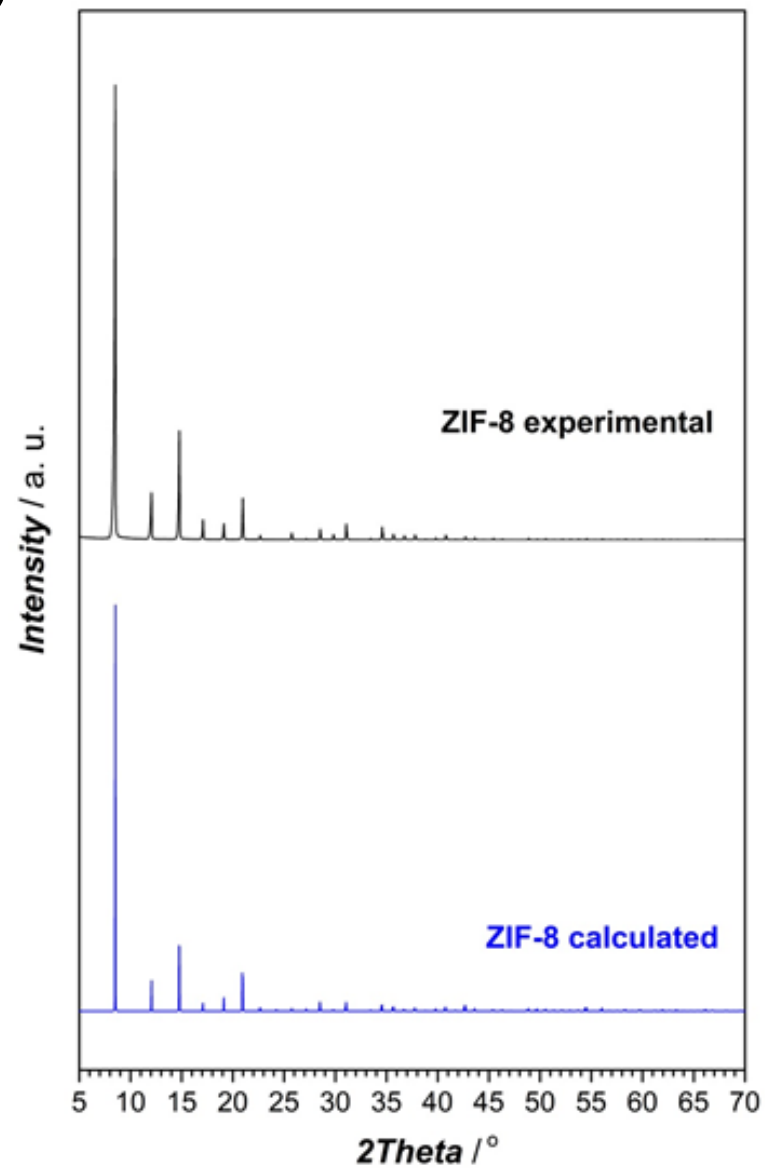

Supplement: Supplementary file 1 [file sensors-19-00888-s001.zip › Images/Fig_S1.pdf]

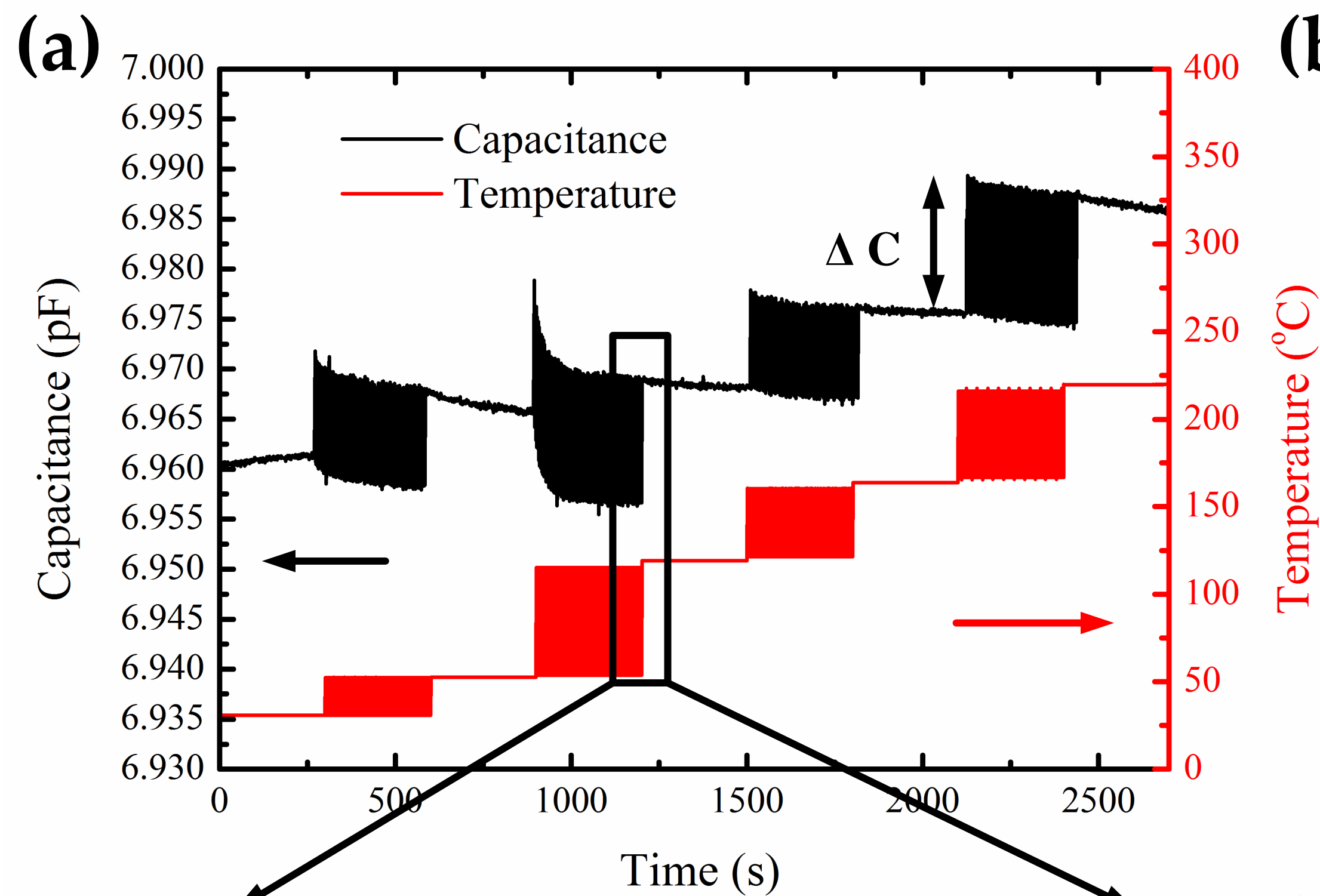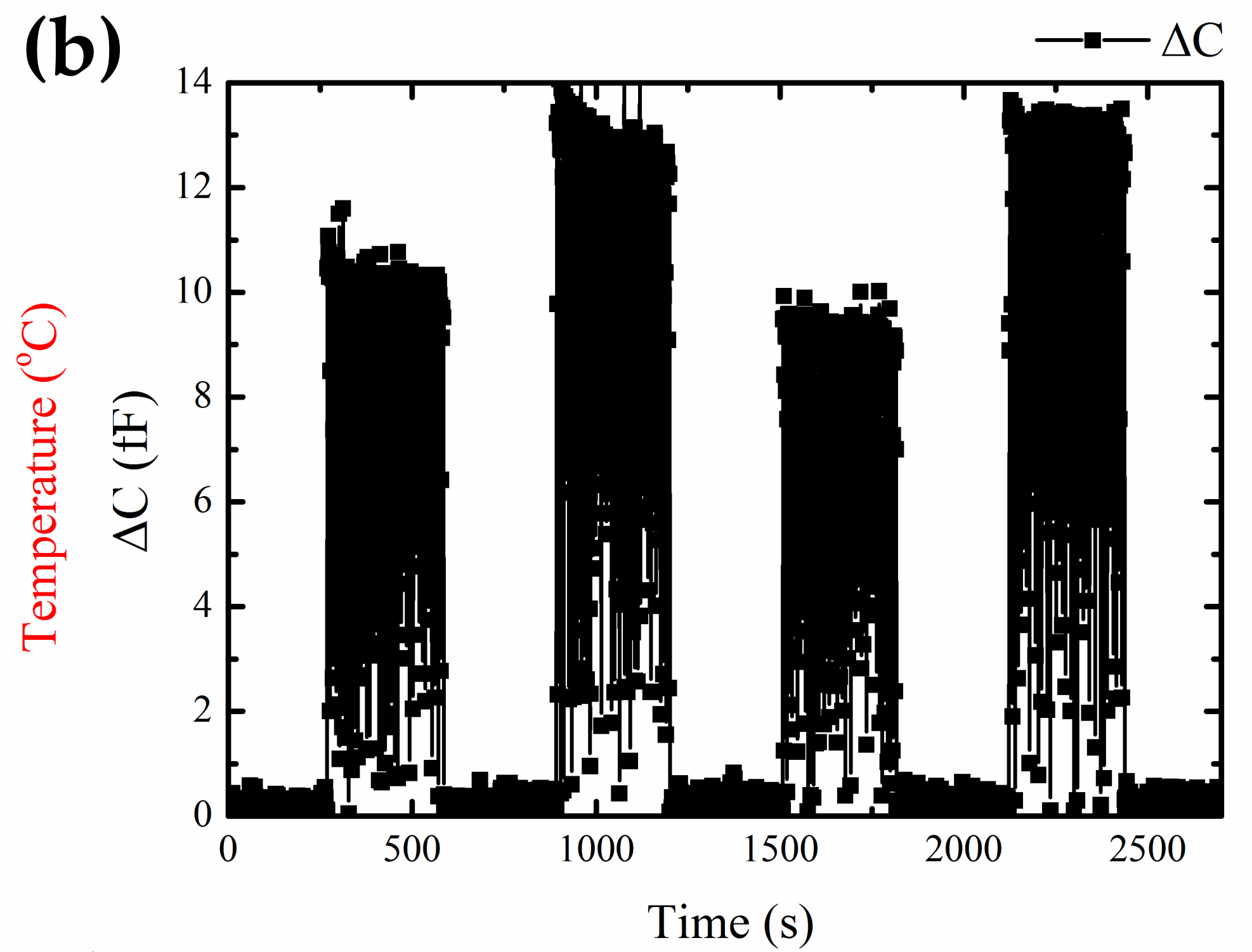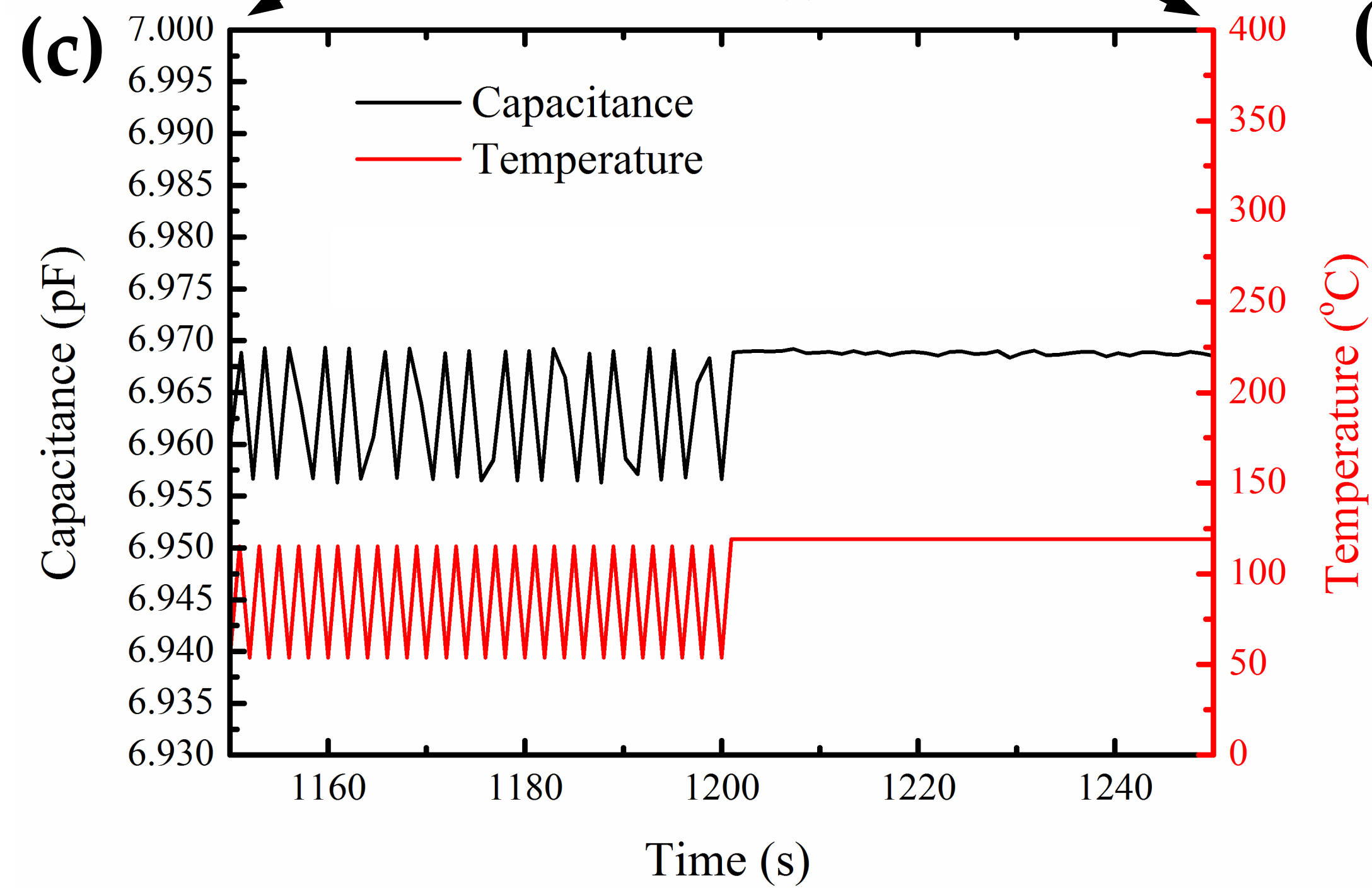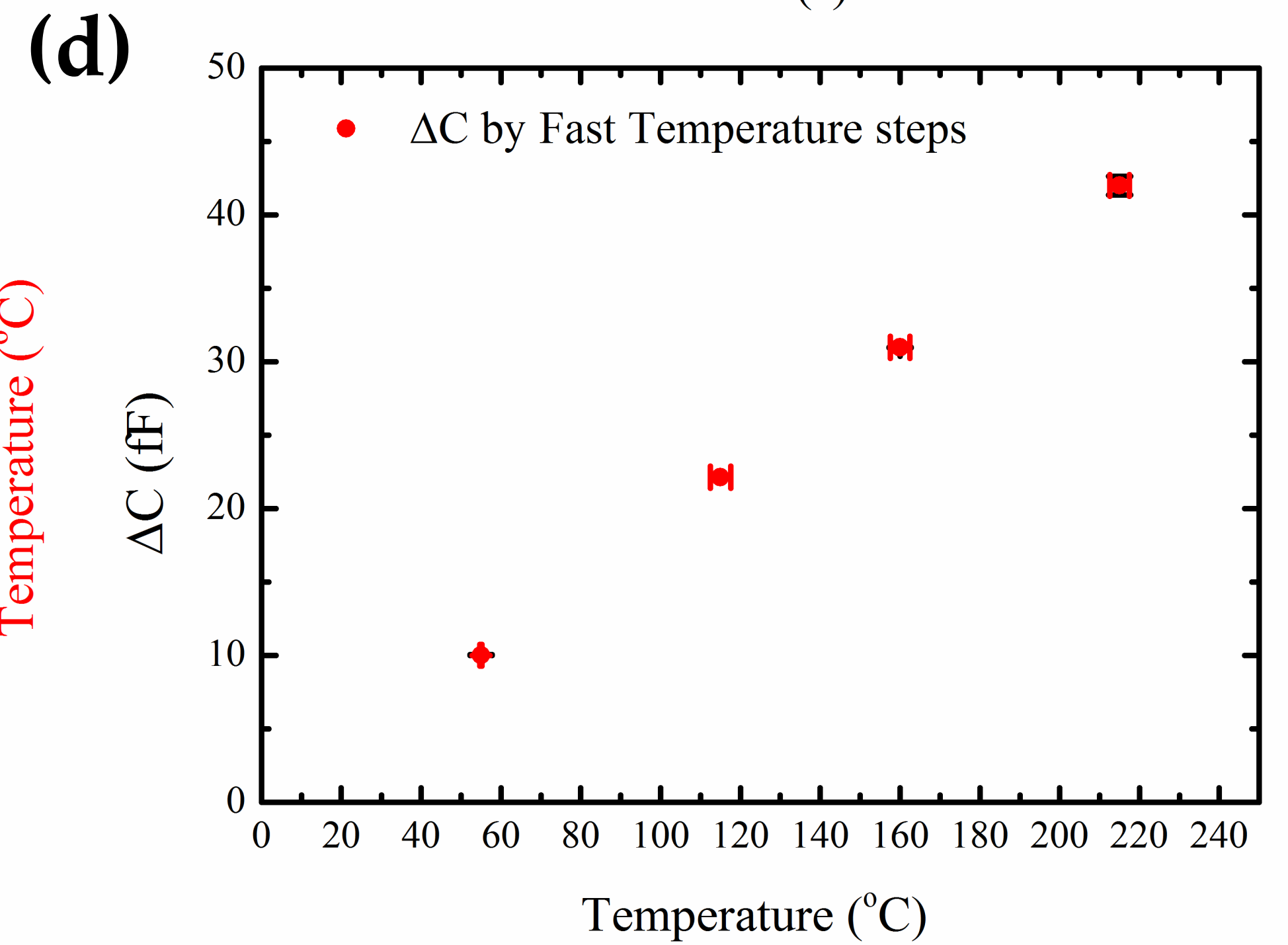

Supplement: Supplementary file 1 [file sensors-19-00888-s001.zip › Images/Fig_S10.pdf]

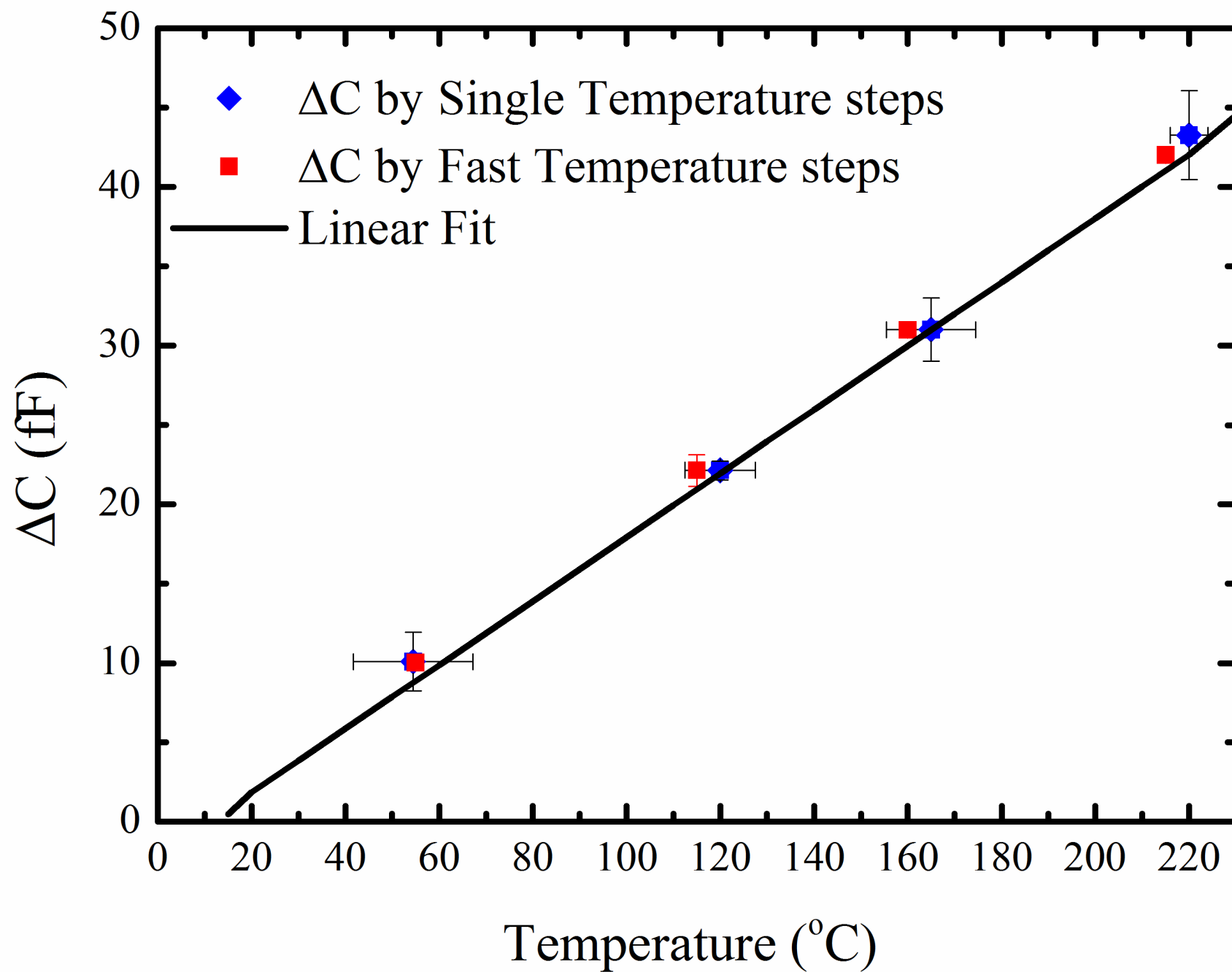

Supplement: Supplementary file 1 [file sensors-19-00888-s001.zip › Images/Fig_S11.pdf]

(a)

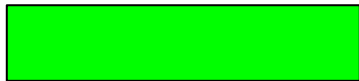

(b)

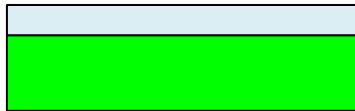

(c)

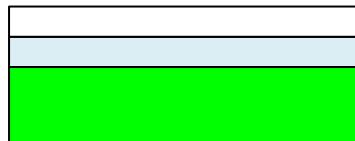

(d)

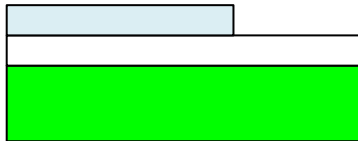

(e)

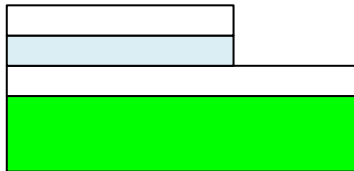

(f)

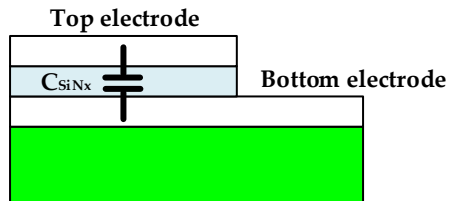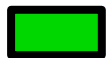

Si

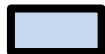

PECVD SiN<sub>x</sub>

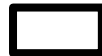

Al

Supplement: Supplementary file 1 [file sensors-19-00888-s001.zip › Images/Fig_S12.pdf]

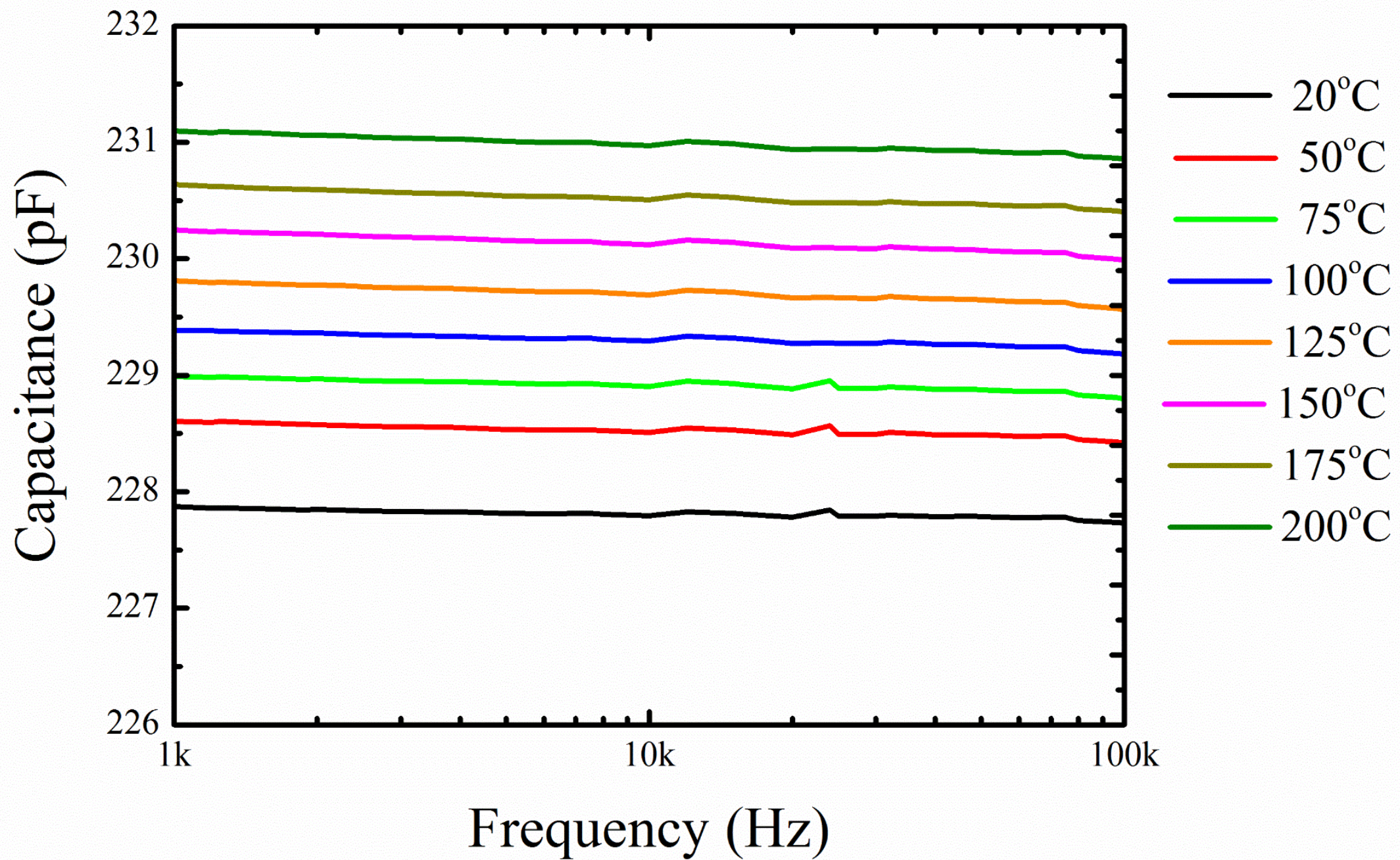

Supplement: Supplementary file 1 [file sensors-19-00888-s001.zip › Images/Fig_S13.pdf]

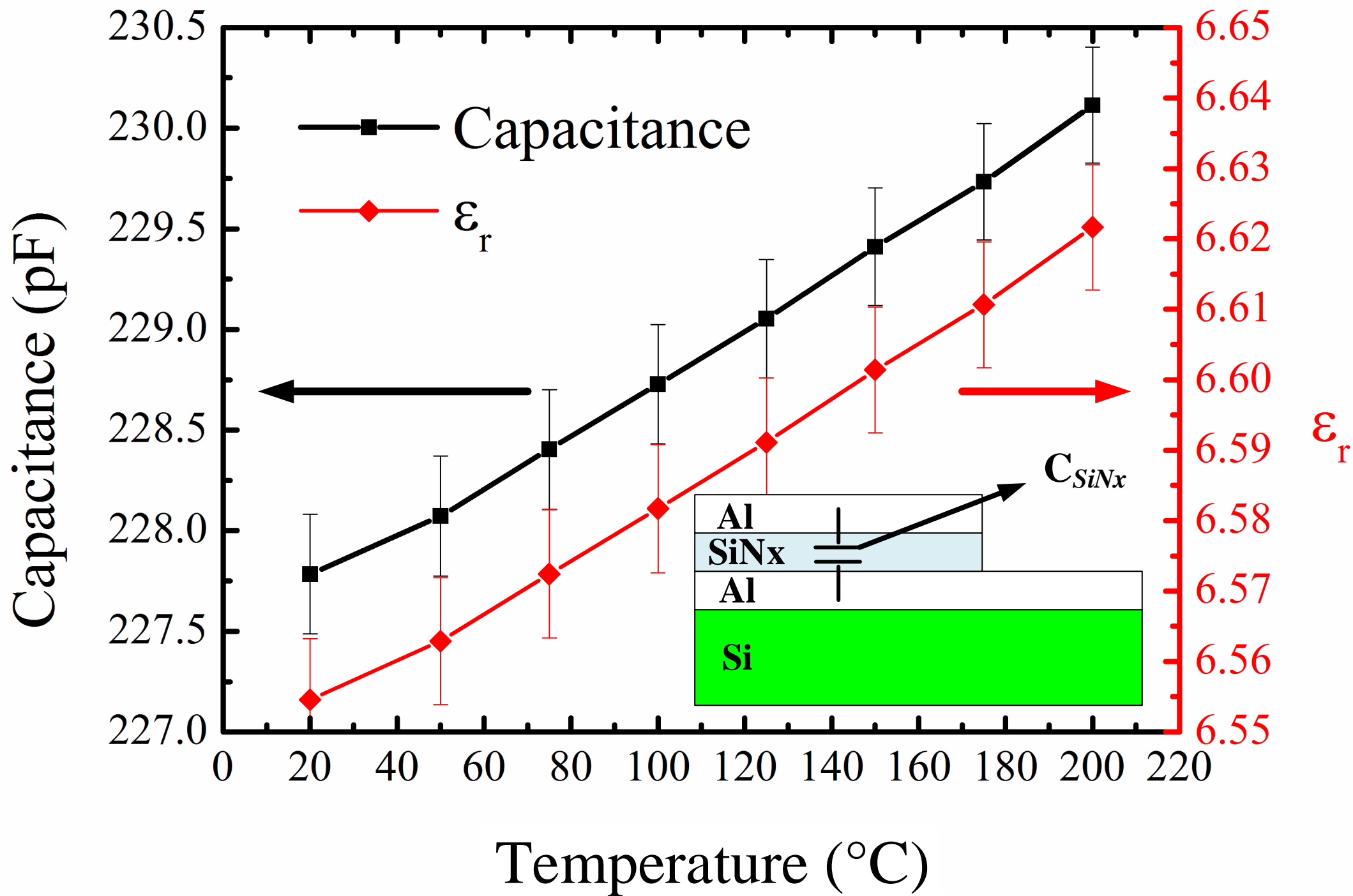

Supplement: Supplementary file 1 [file sensors-19-00888-s001.zip › Images/Fig_S14.pdf]

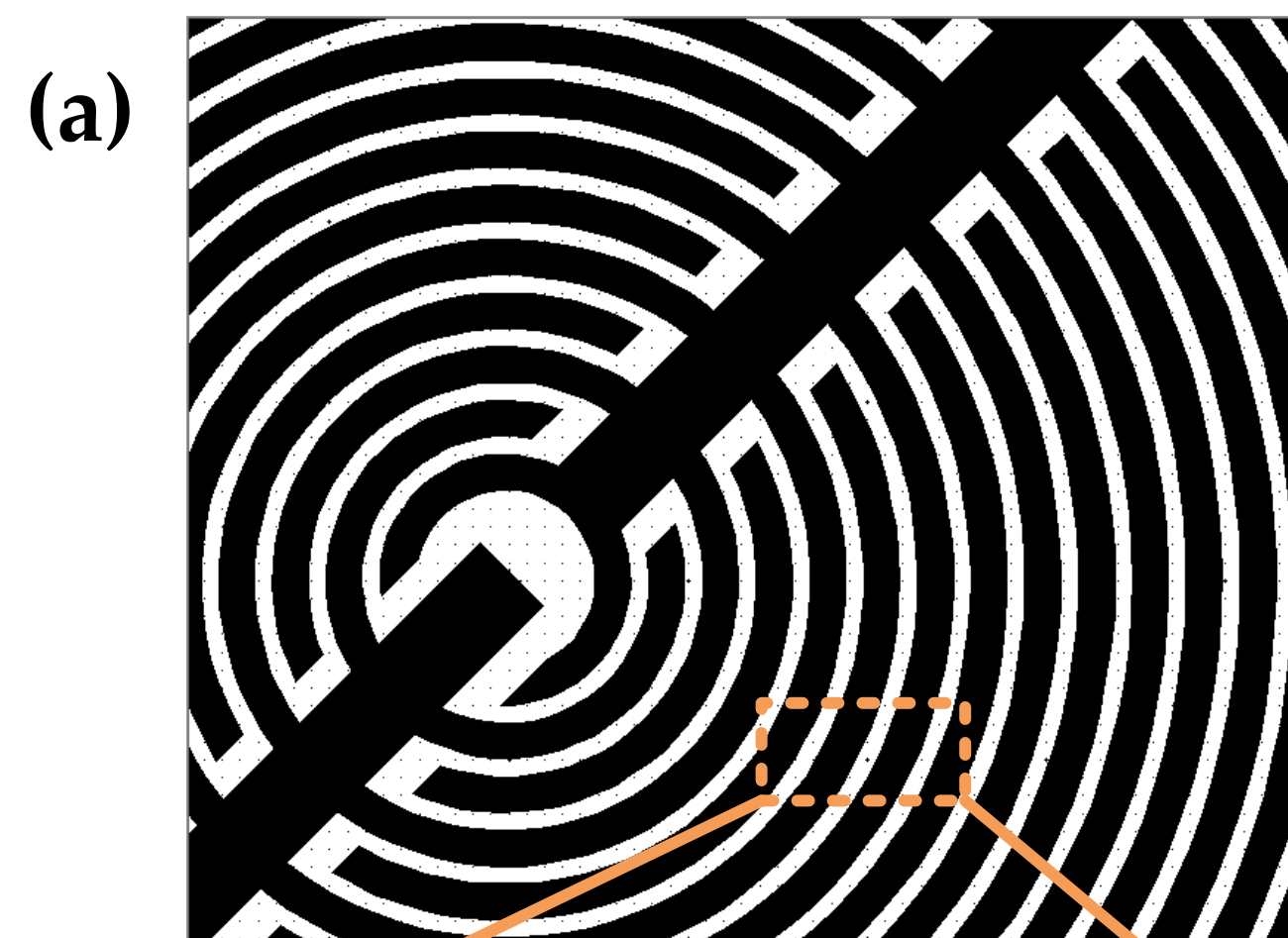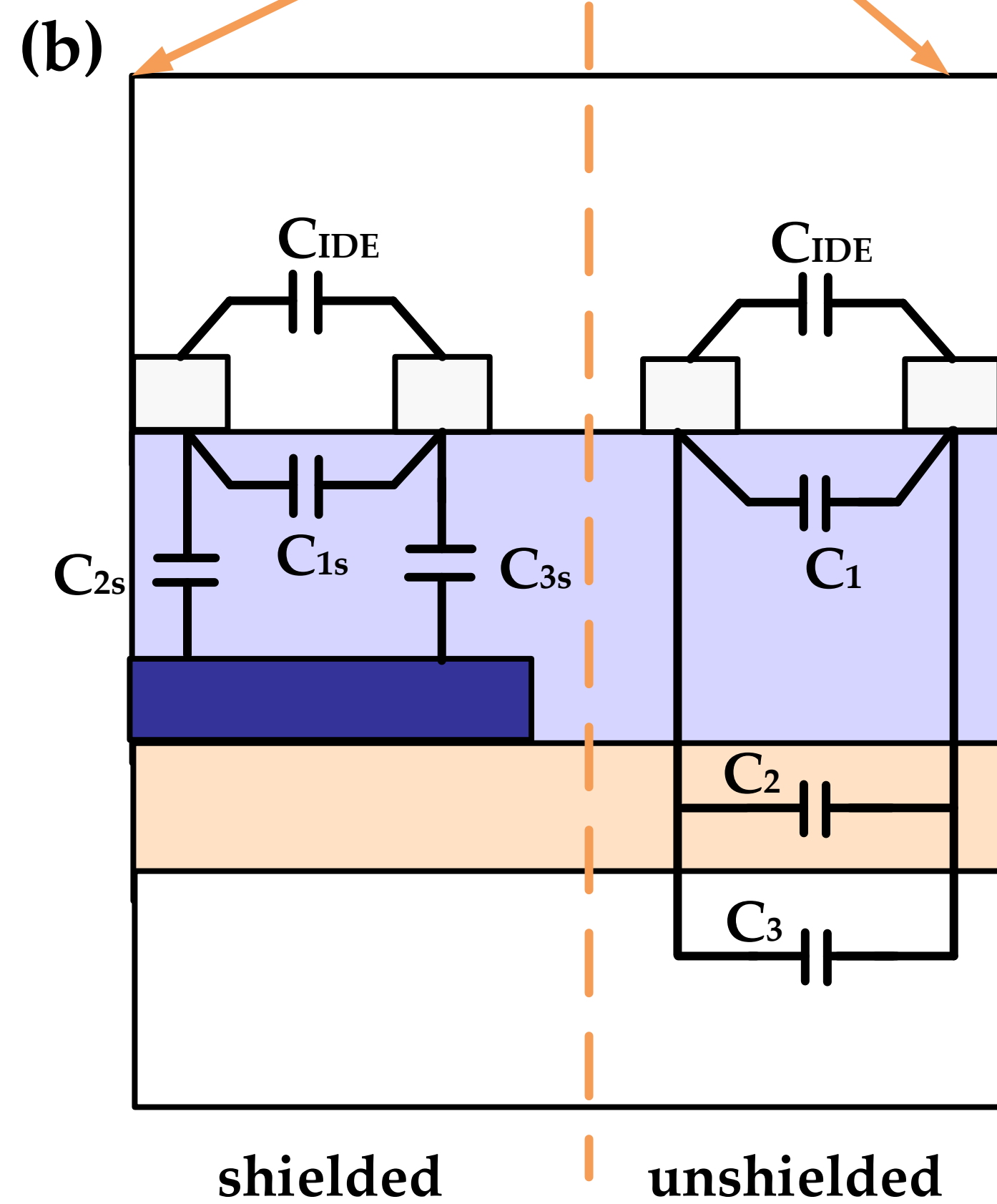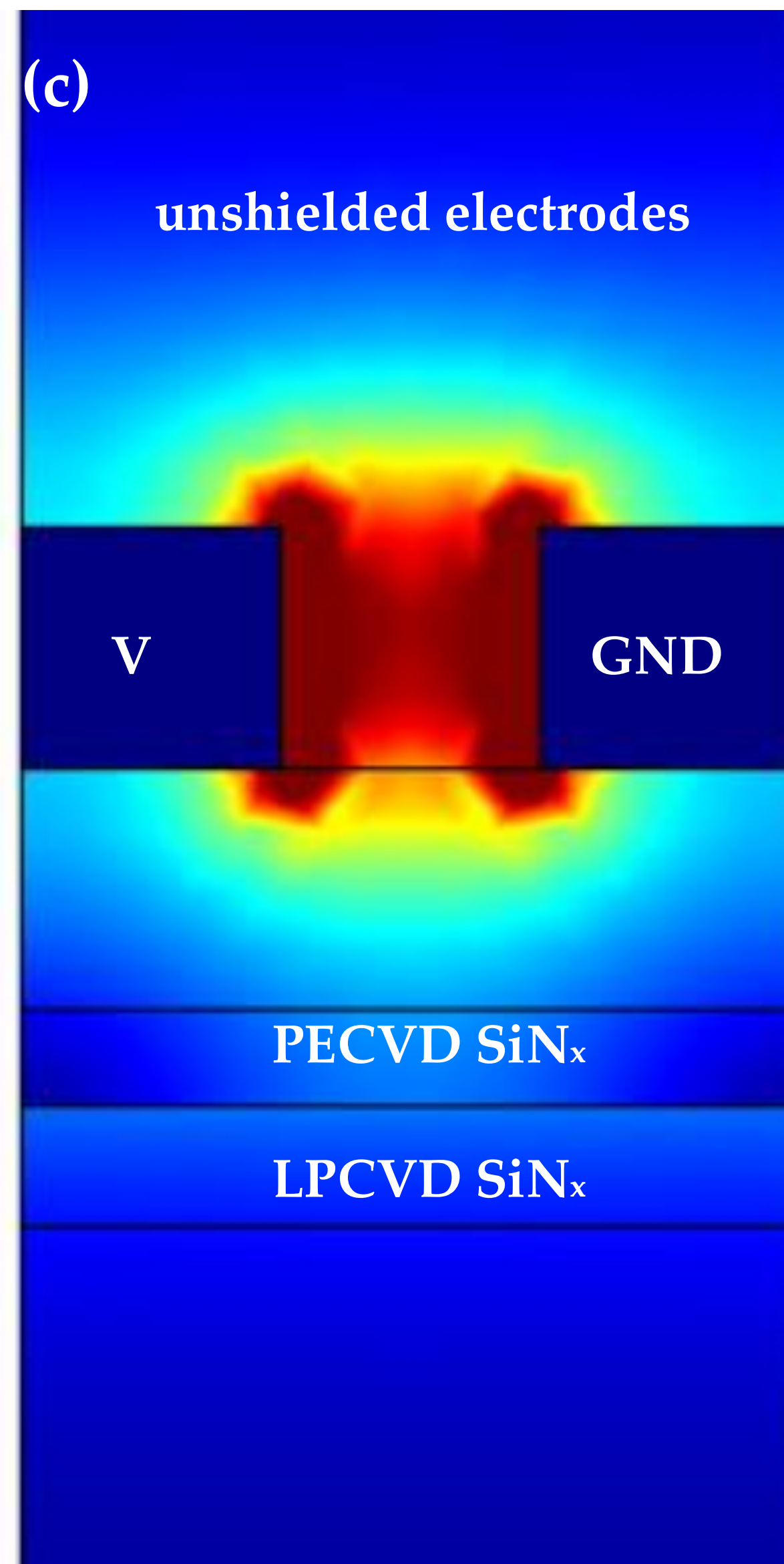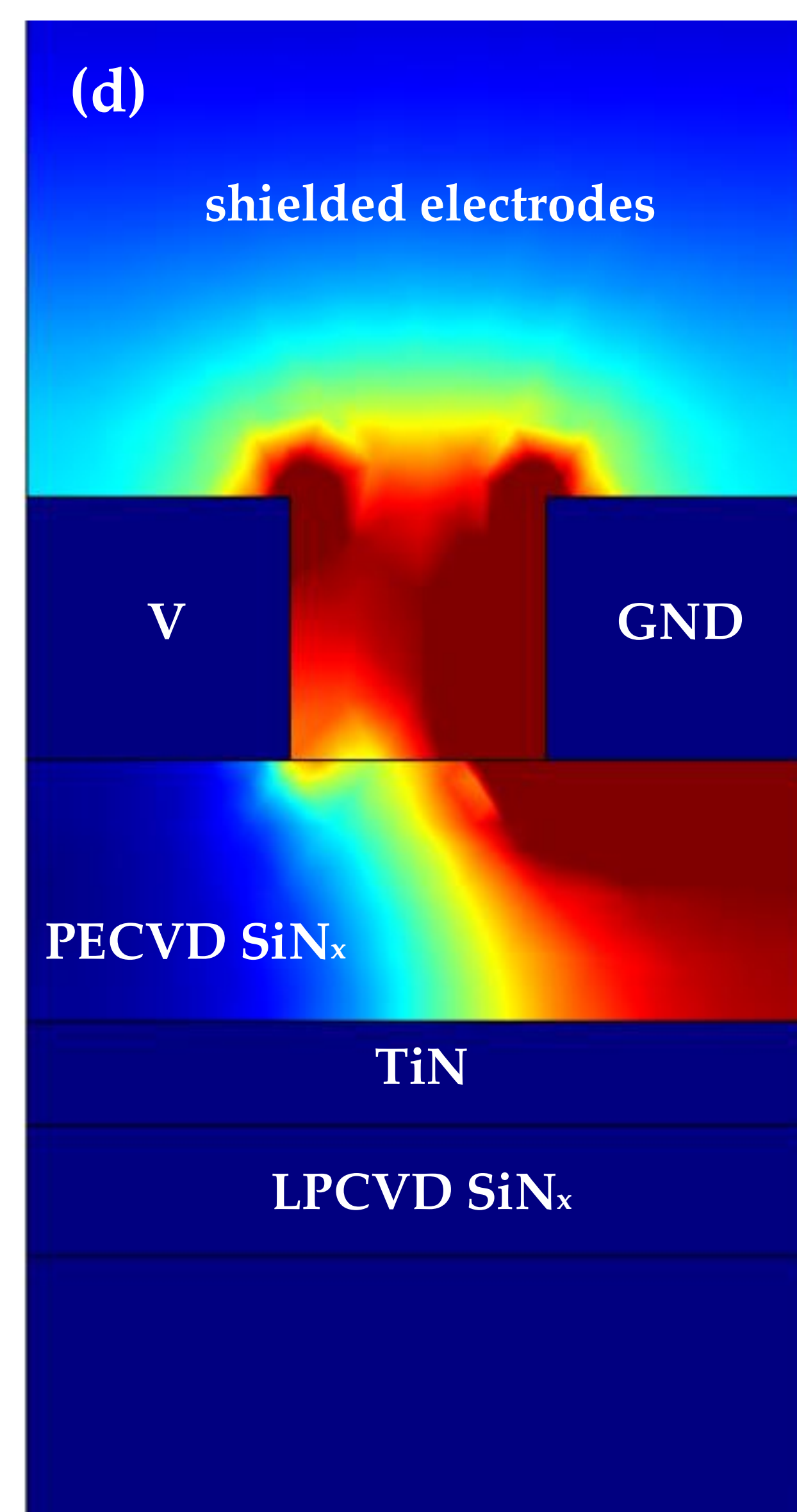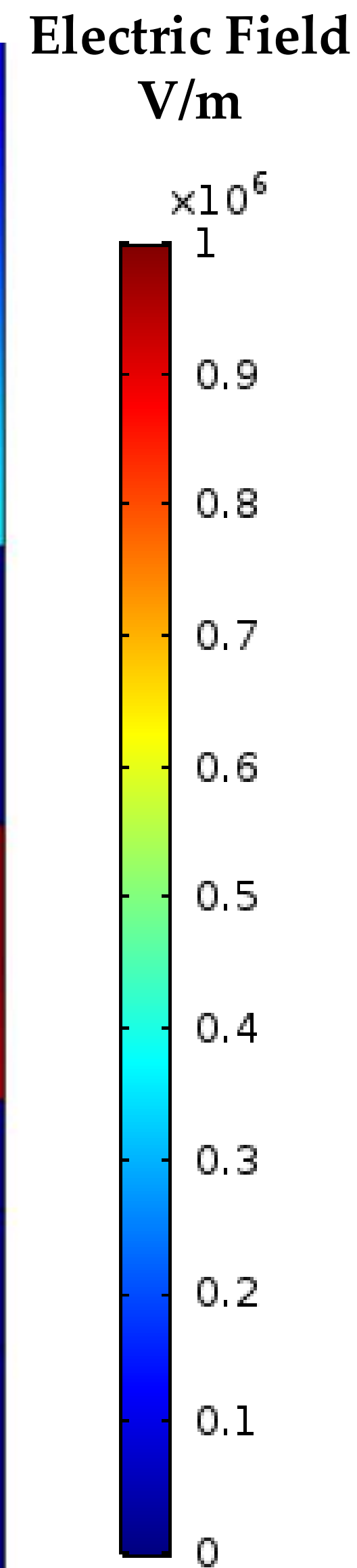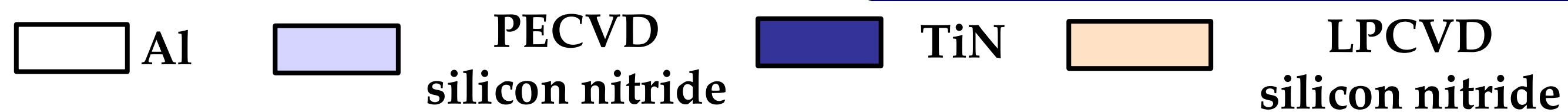

Supplement: Supplementary file 1 [file sensors-19-00888-s001.zip › Images/Fig_S15.pdf]

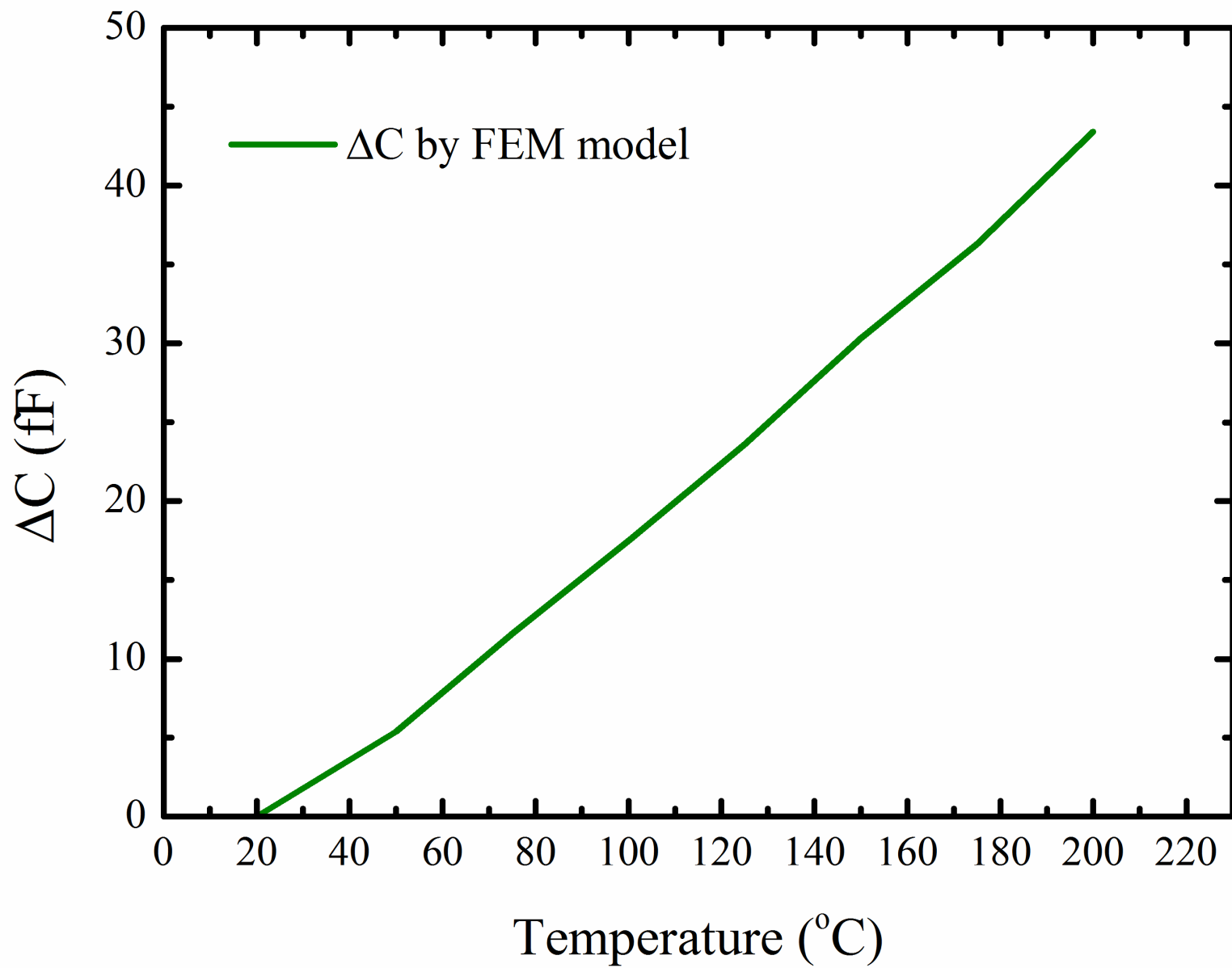

Supplement: Supplementary file 1 [file sensors-19-00888-s001.zip › Images/Fig_S16.pdf]

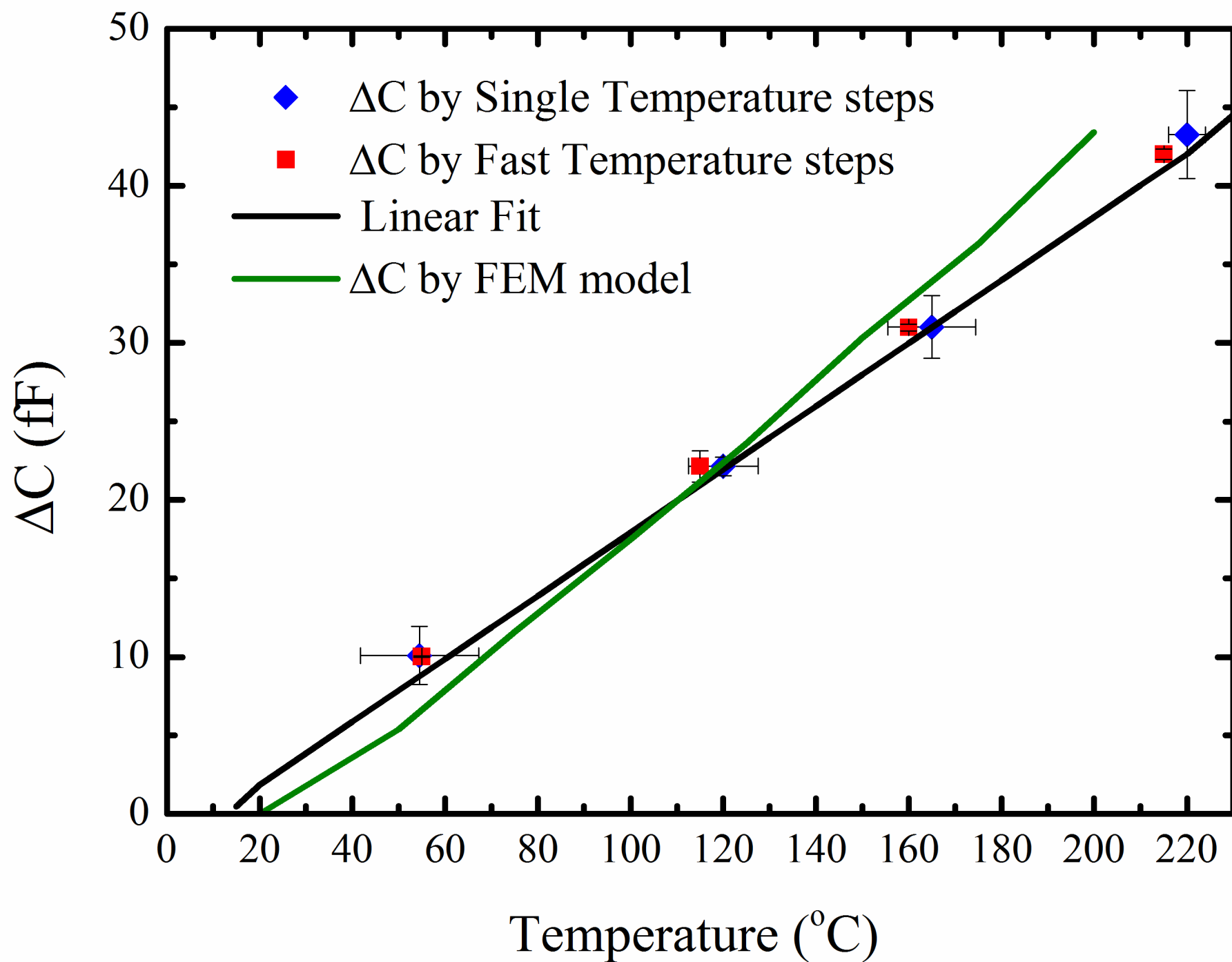

Supplement: Supplementary file 1 [file sensors-19-00888-s001.zip › Images/Fig_S17.pdf]

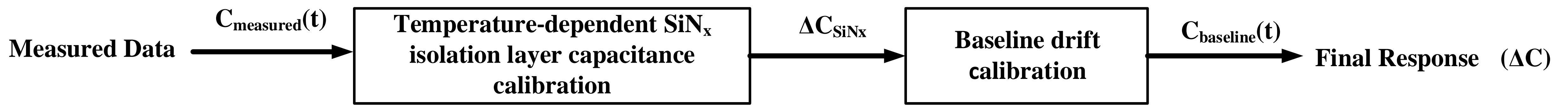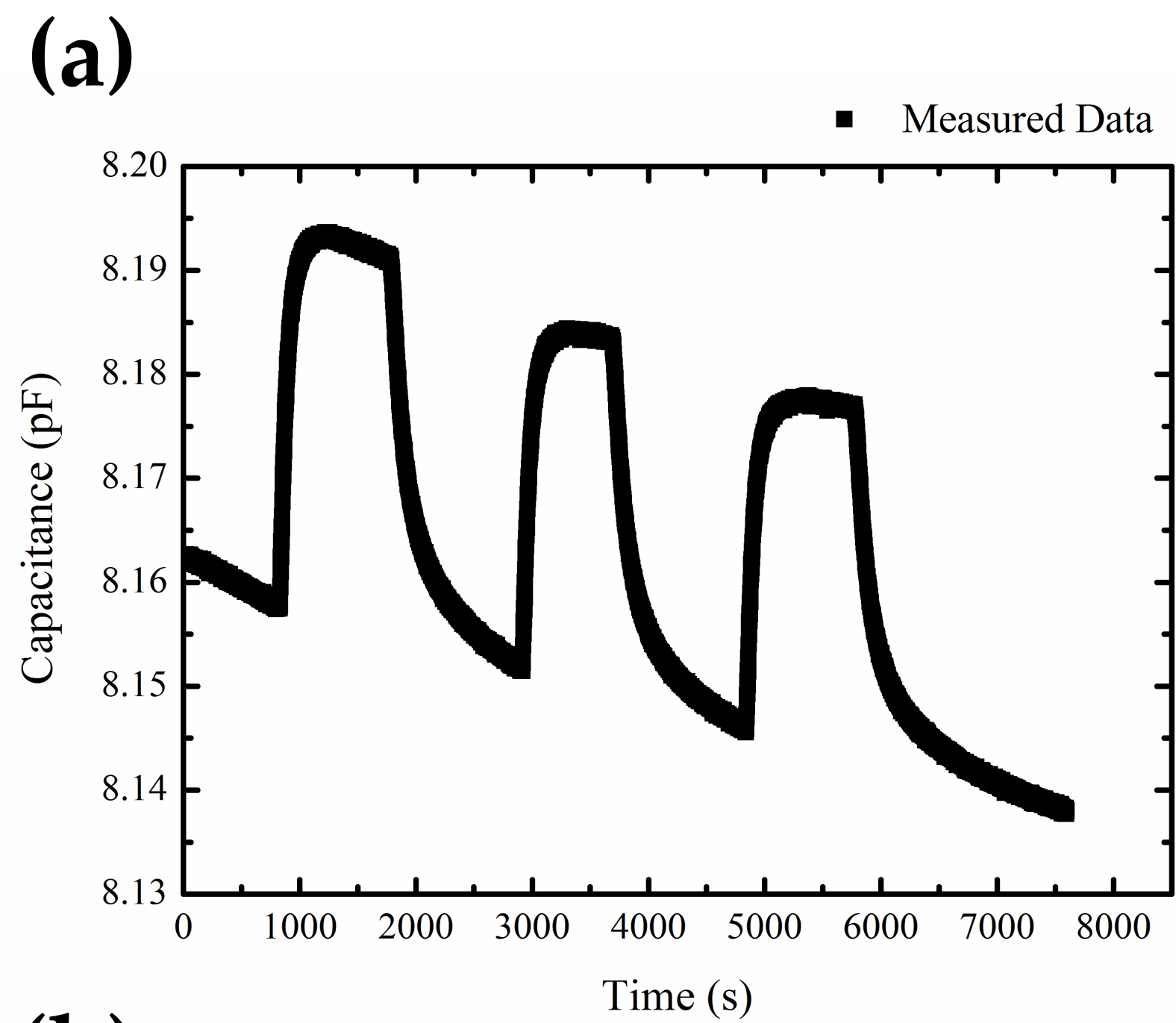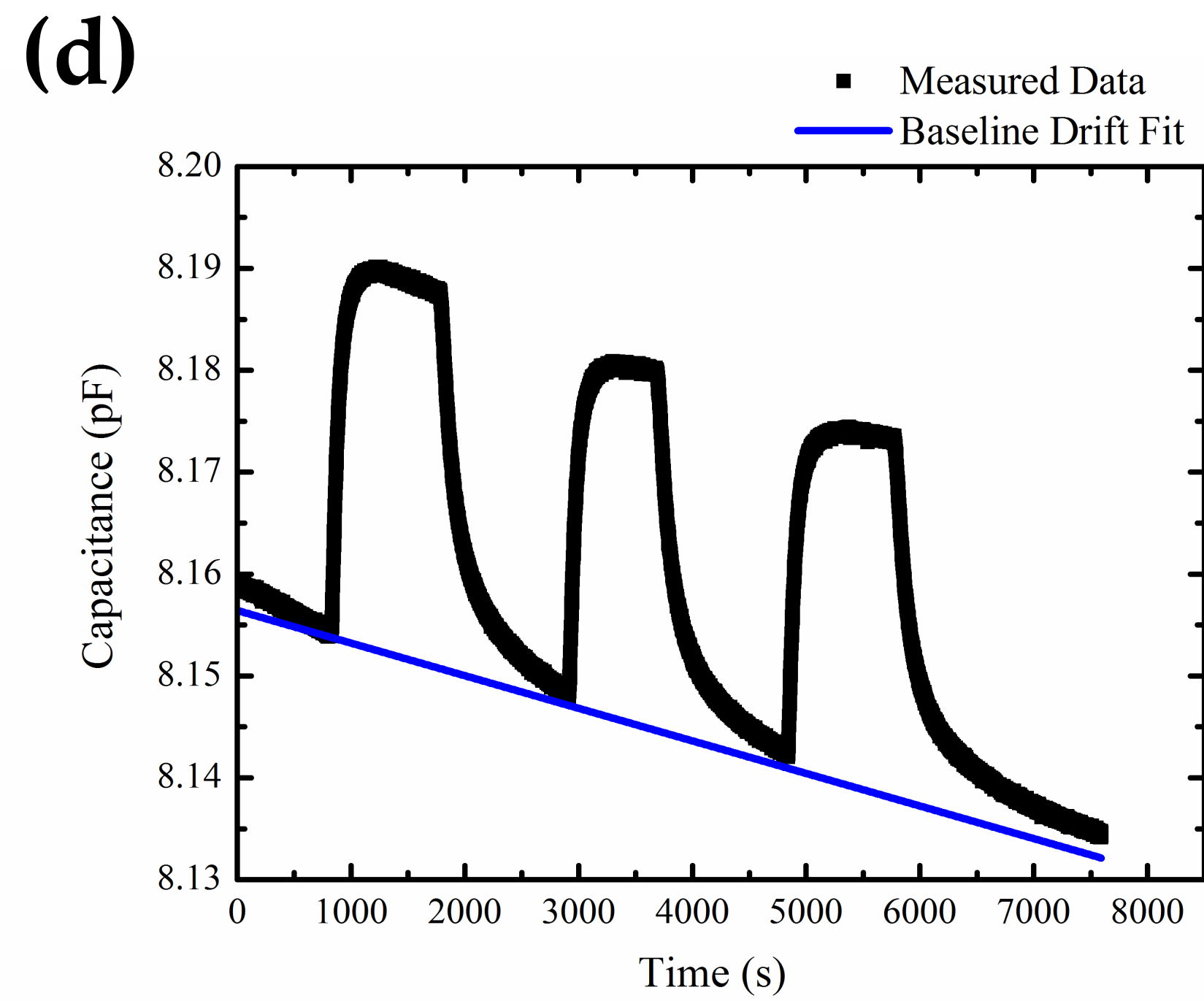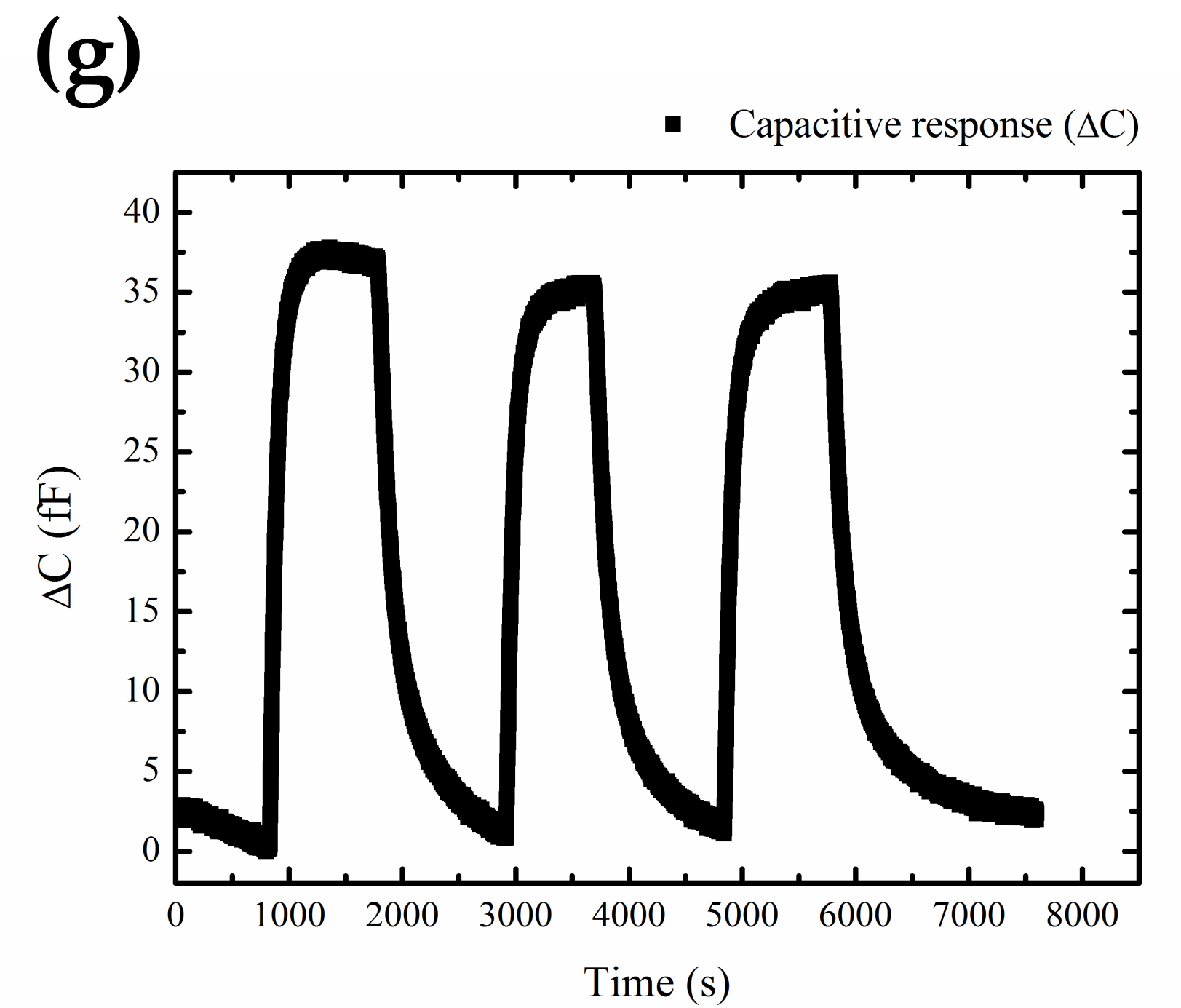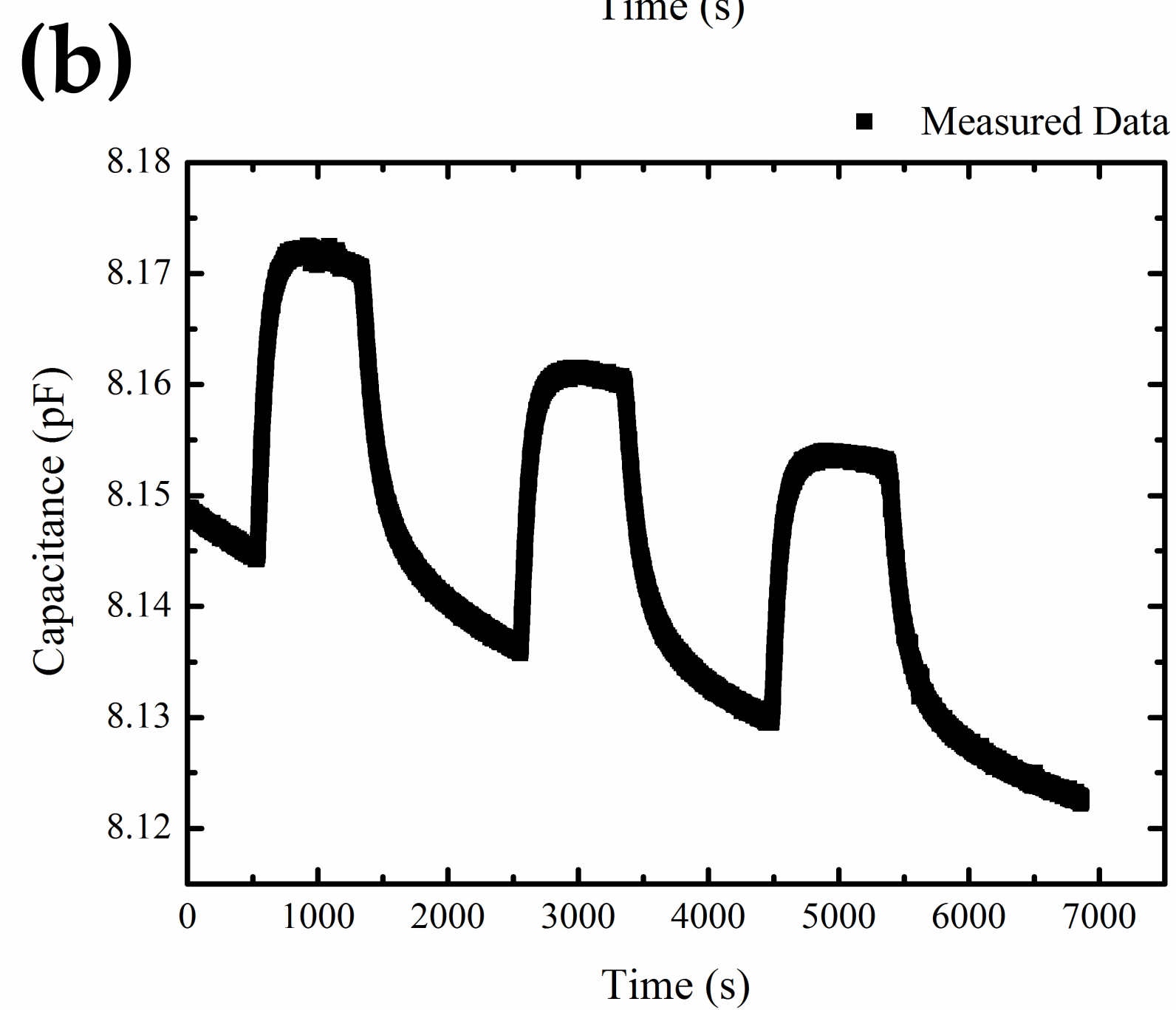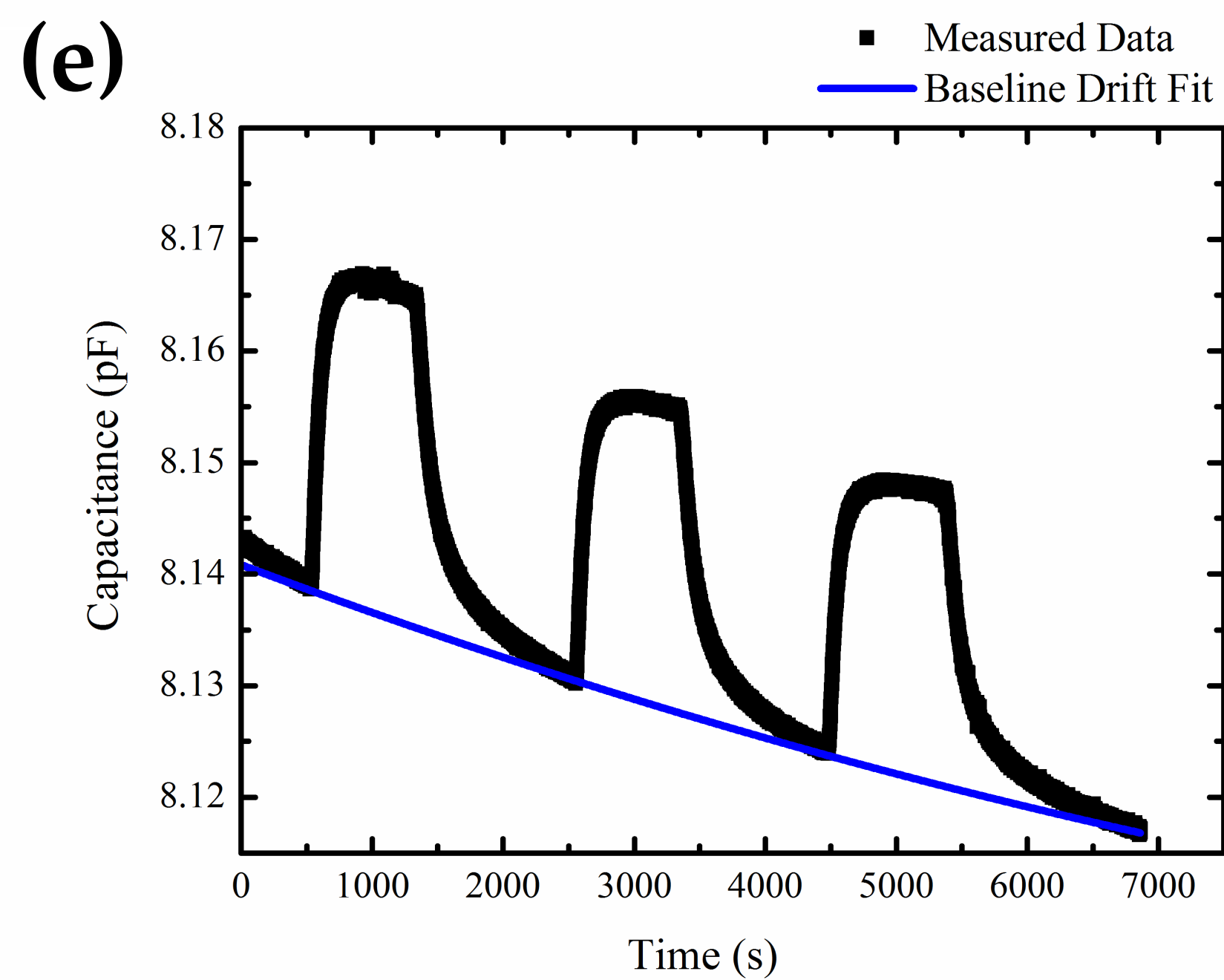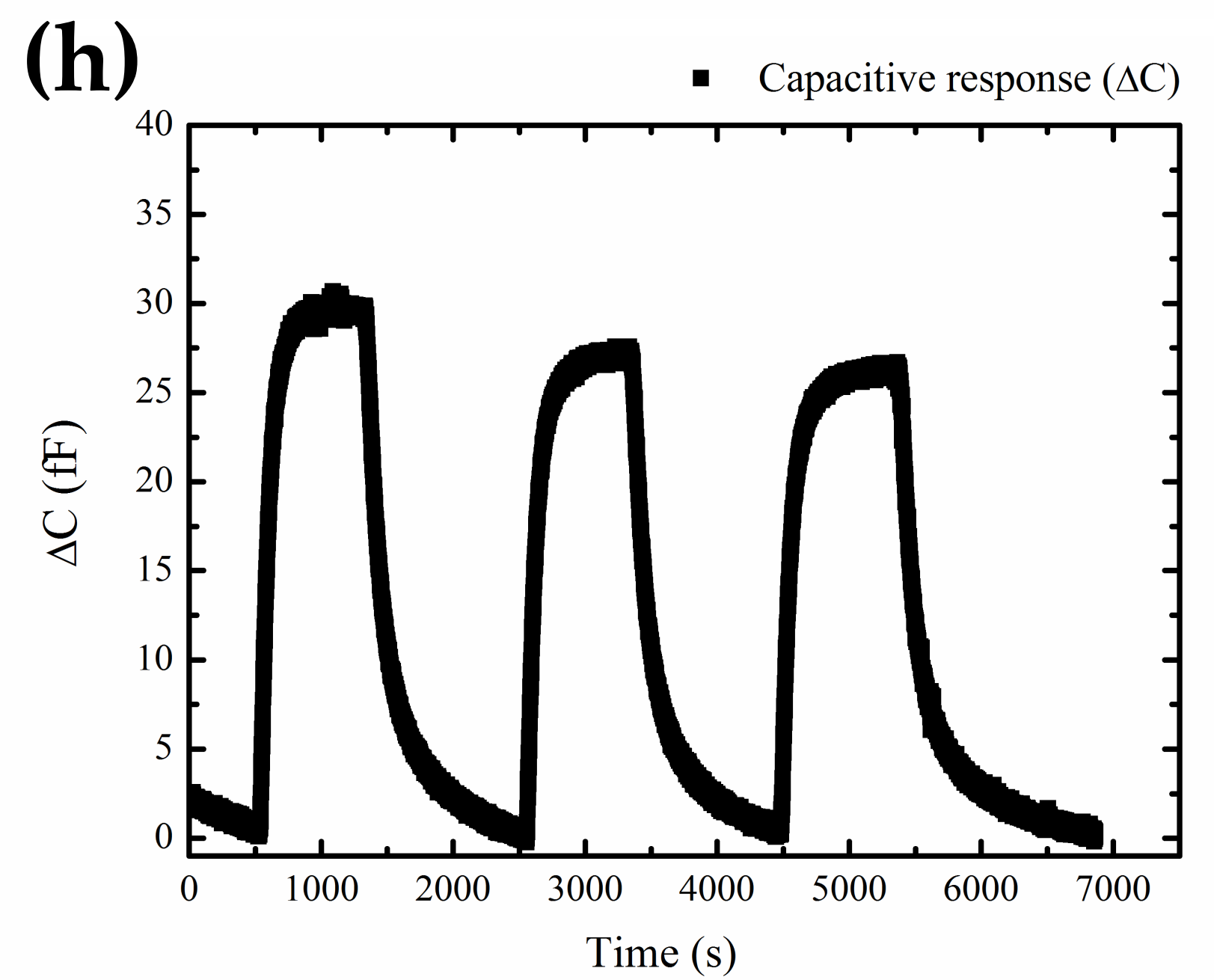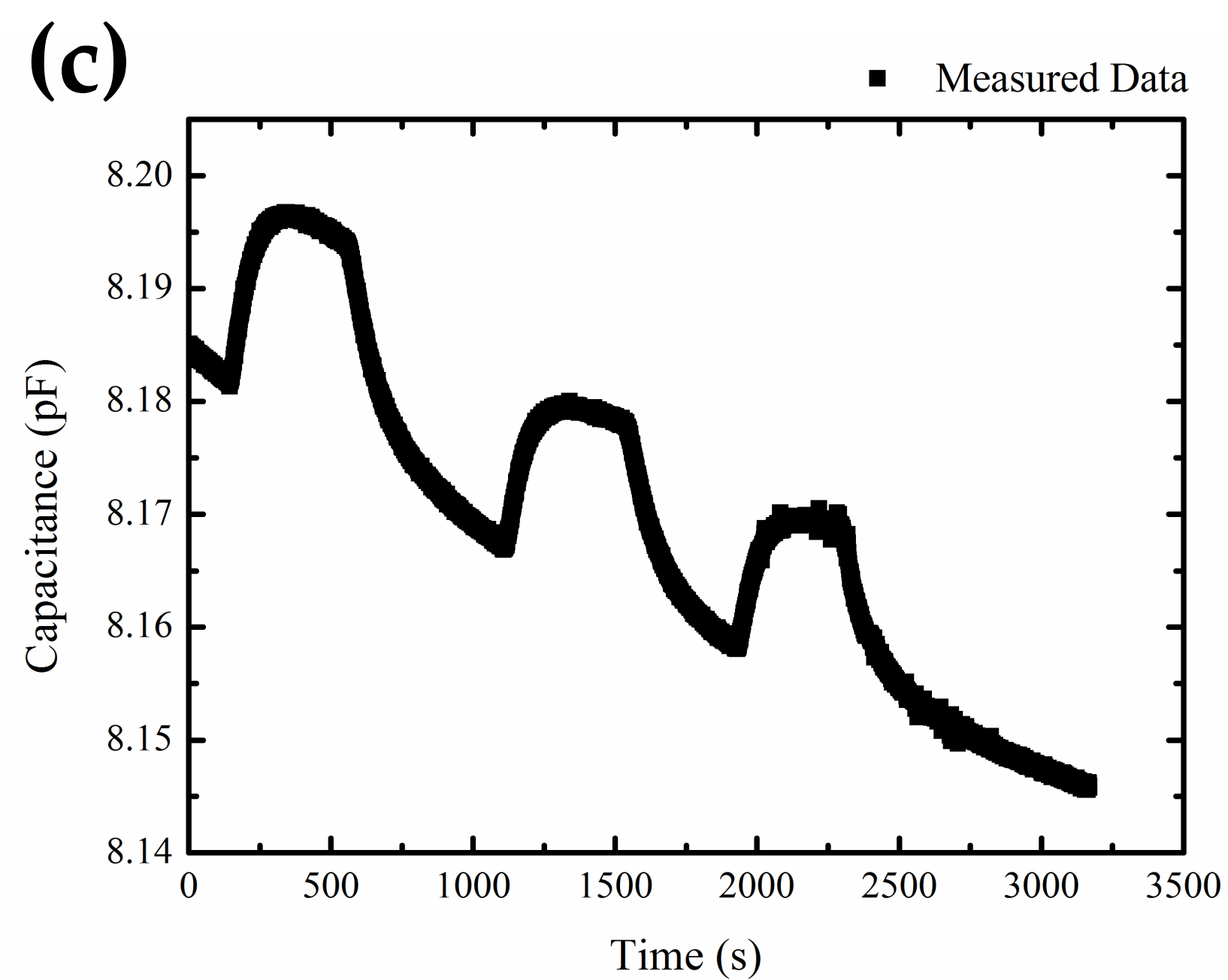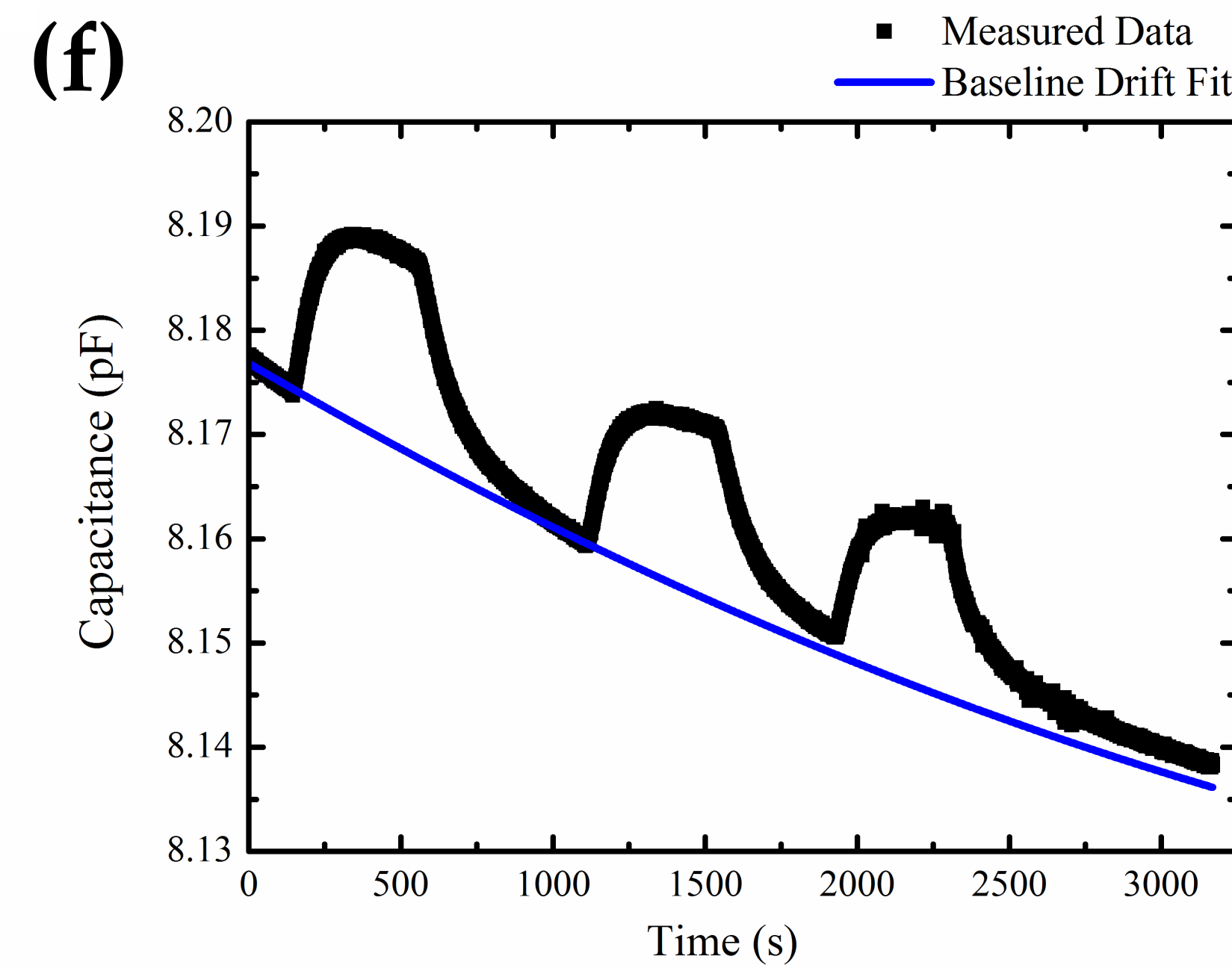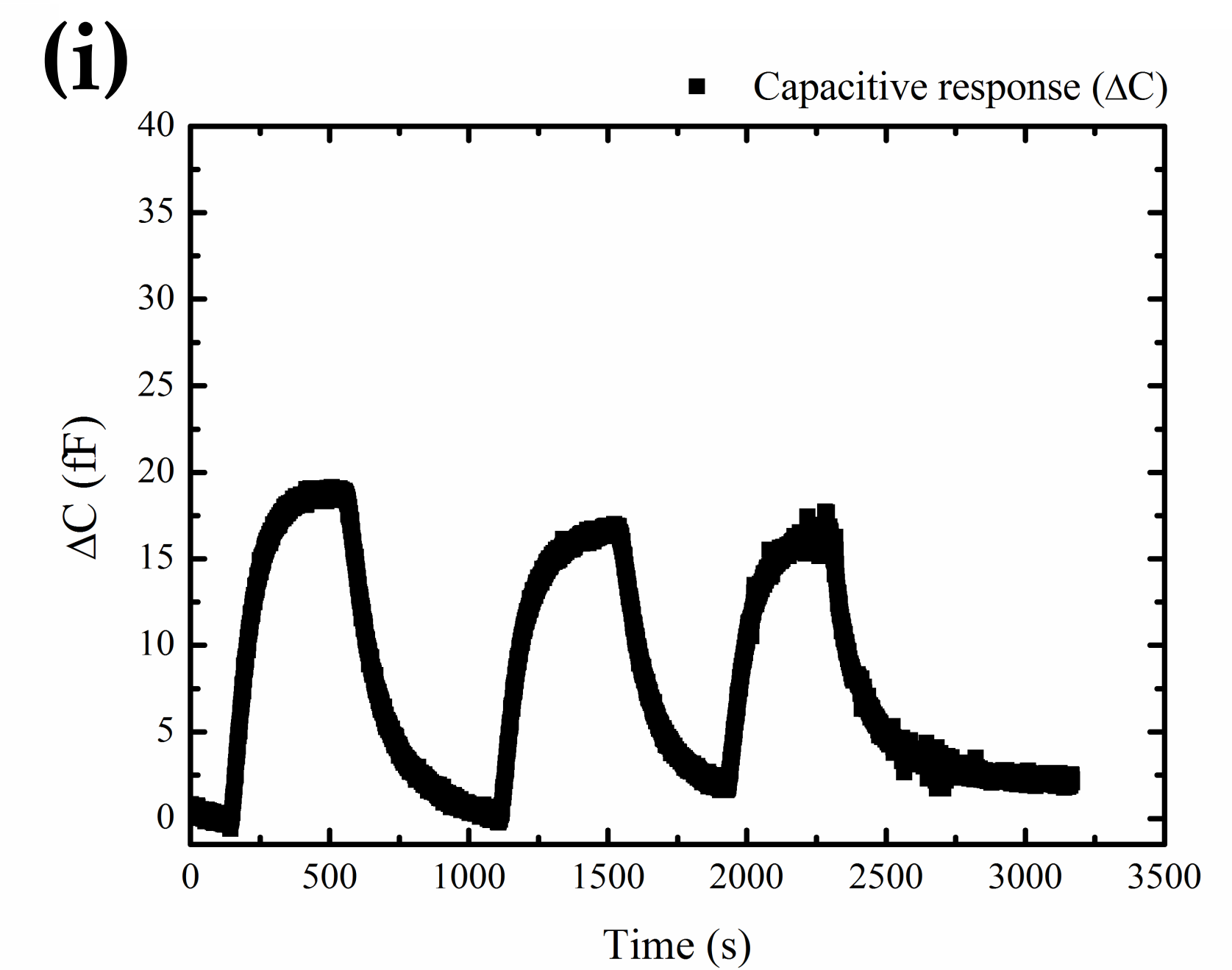

Supplement: Supplementary file 1 [file sensors-19-00888-s001.zip › Images/Fig_S18.pdf]

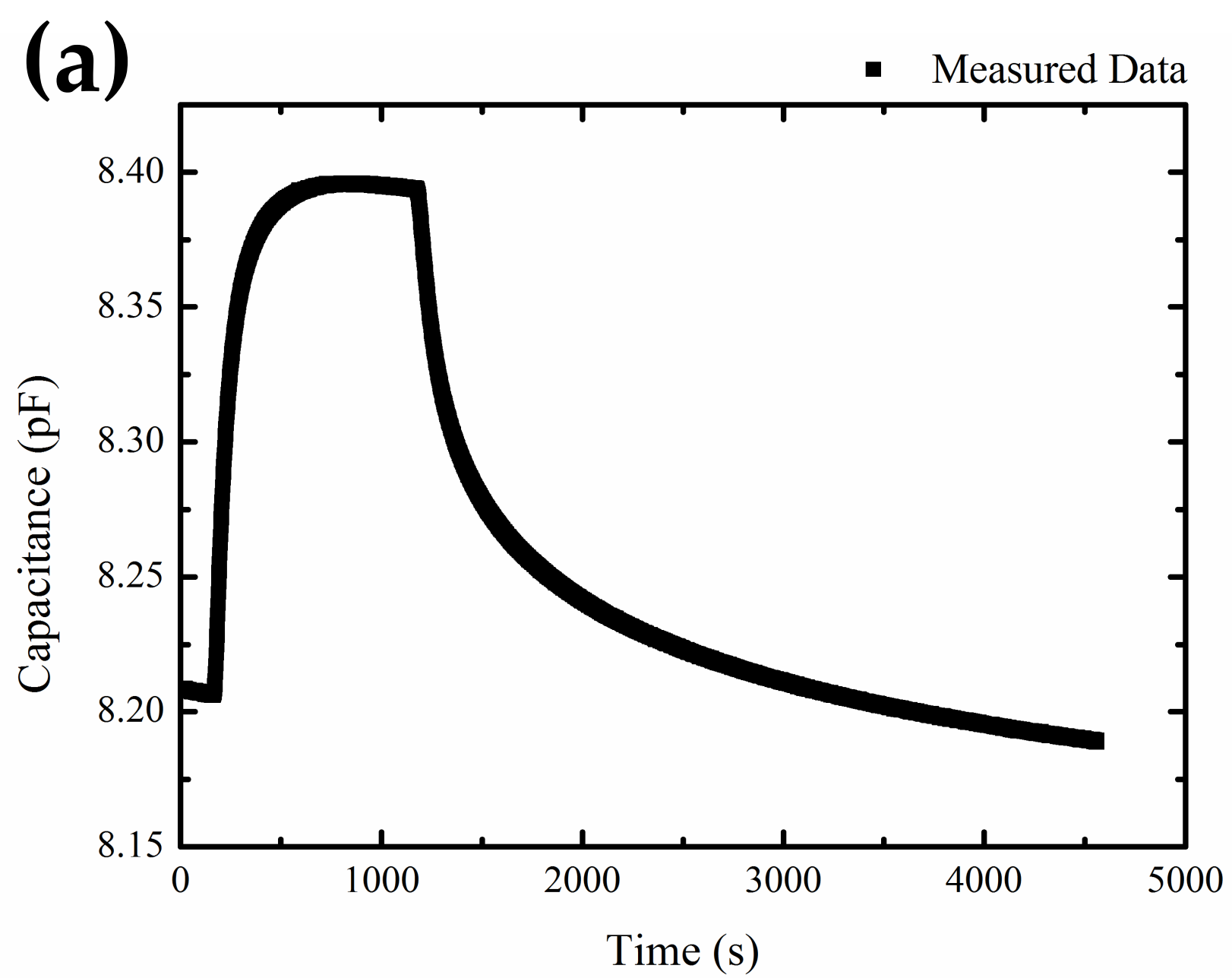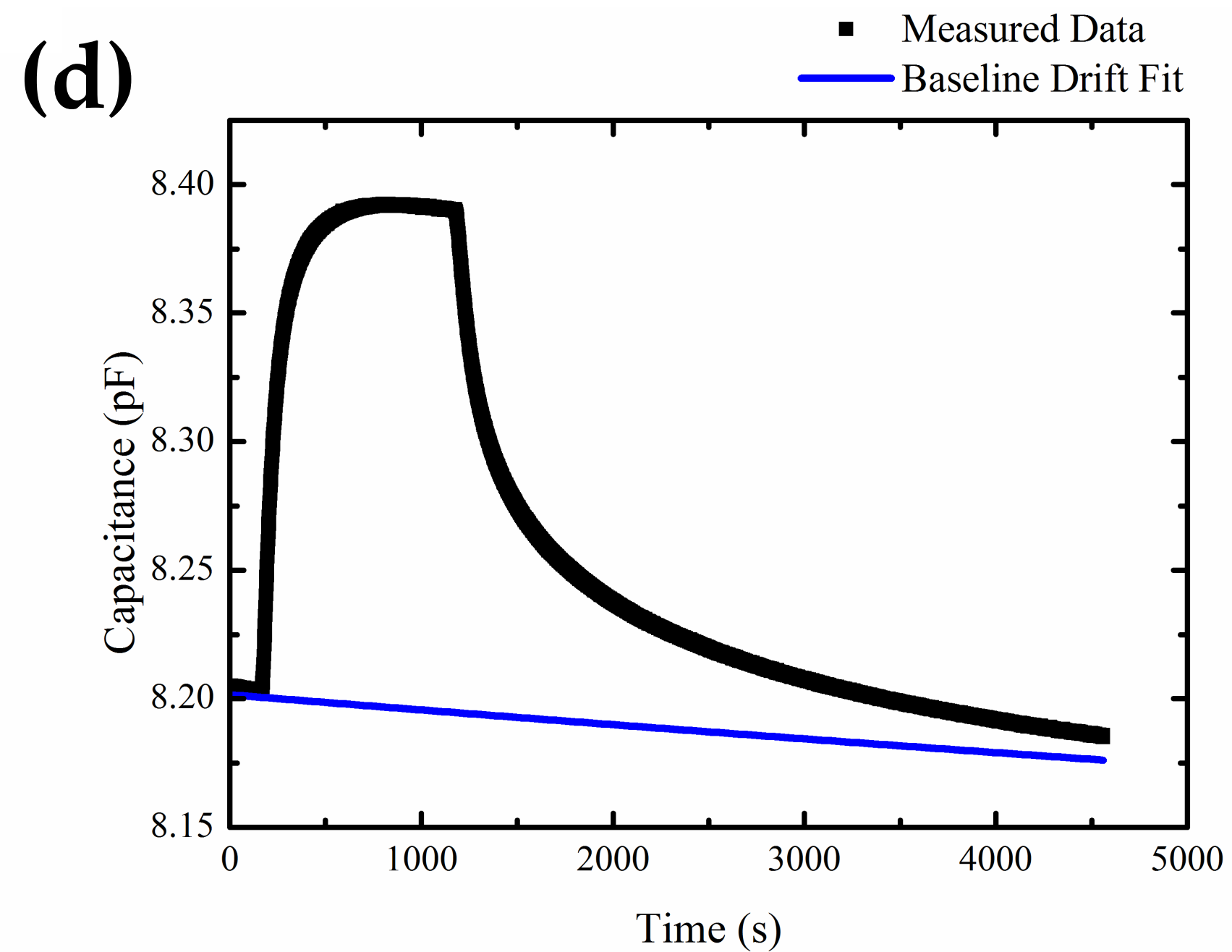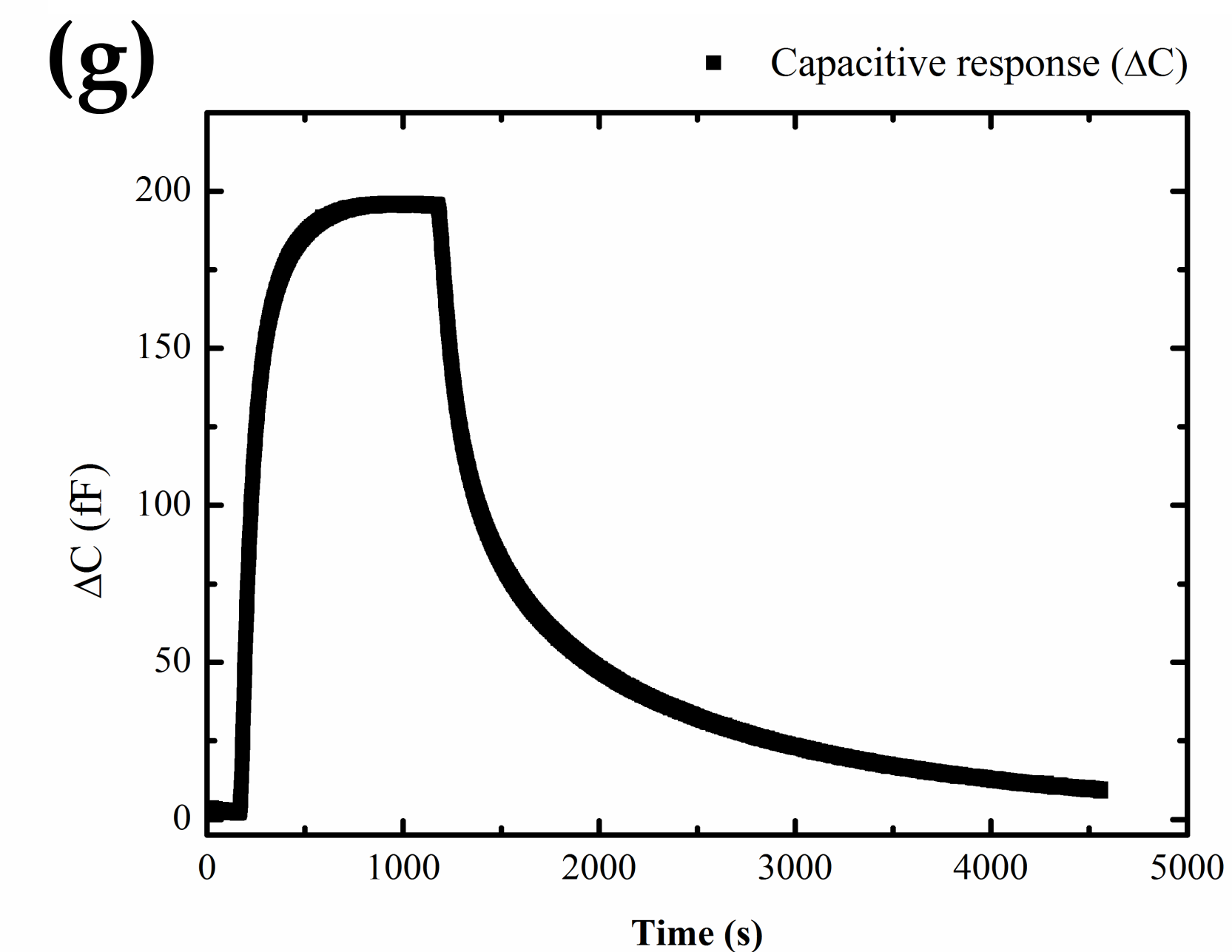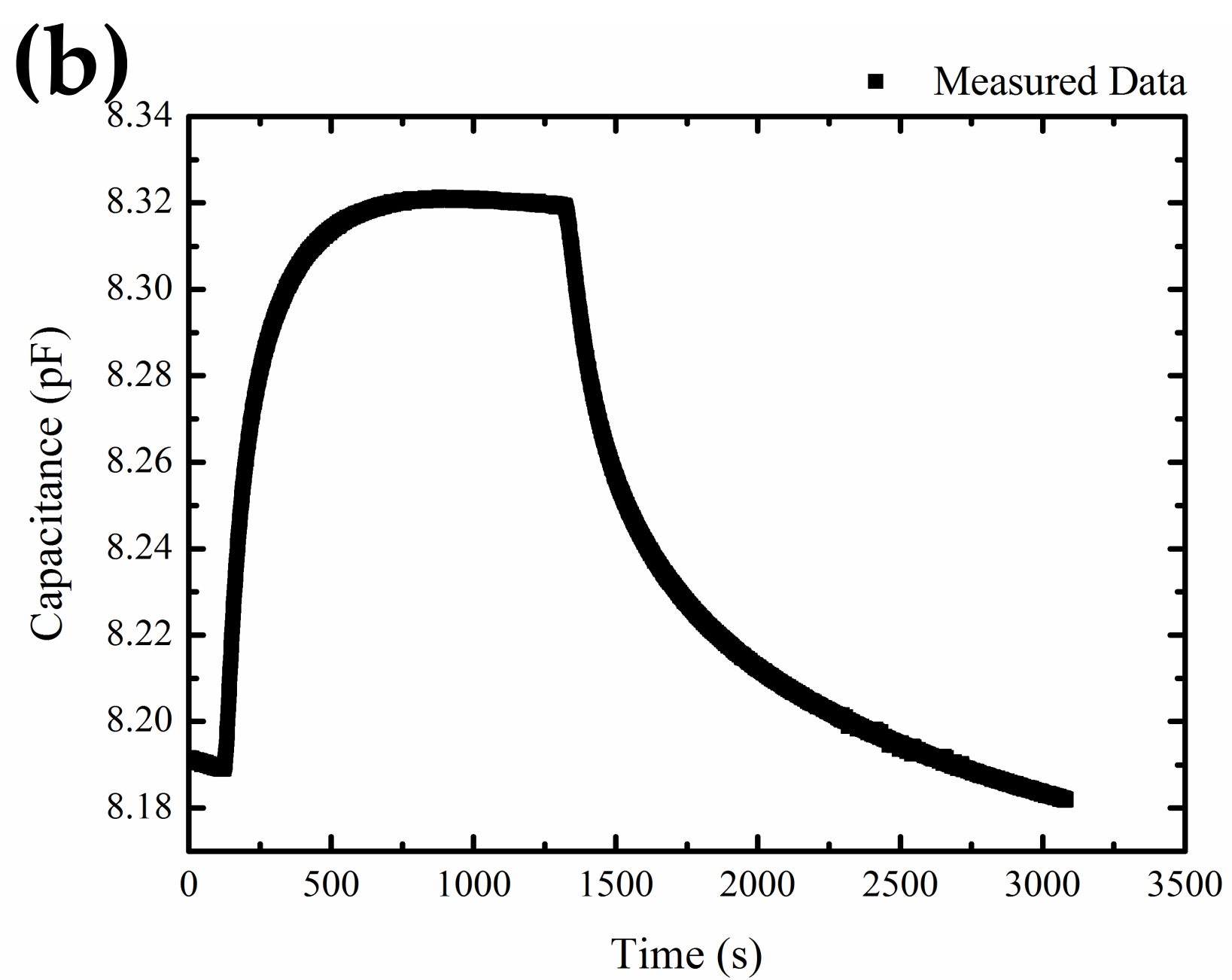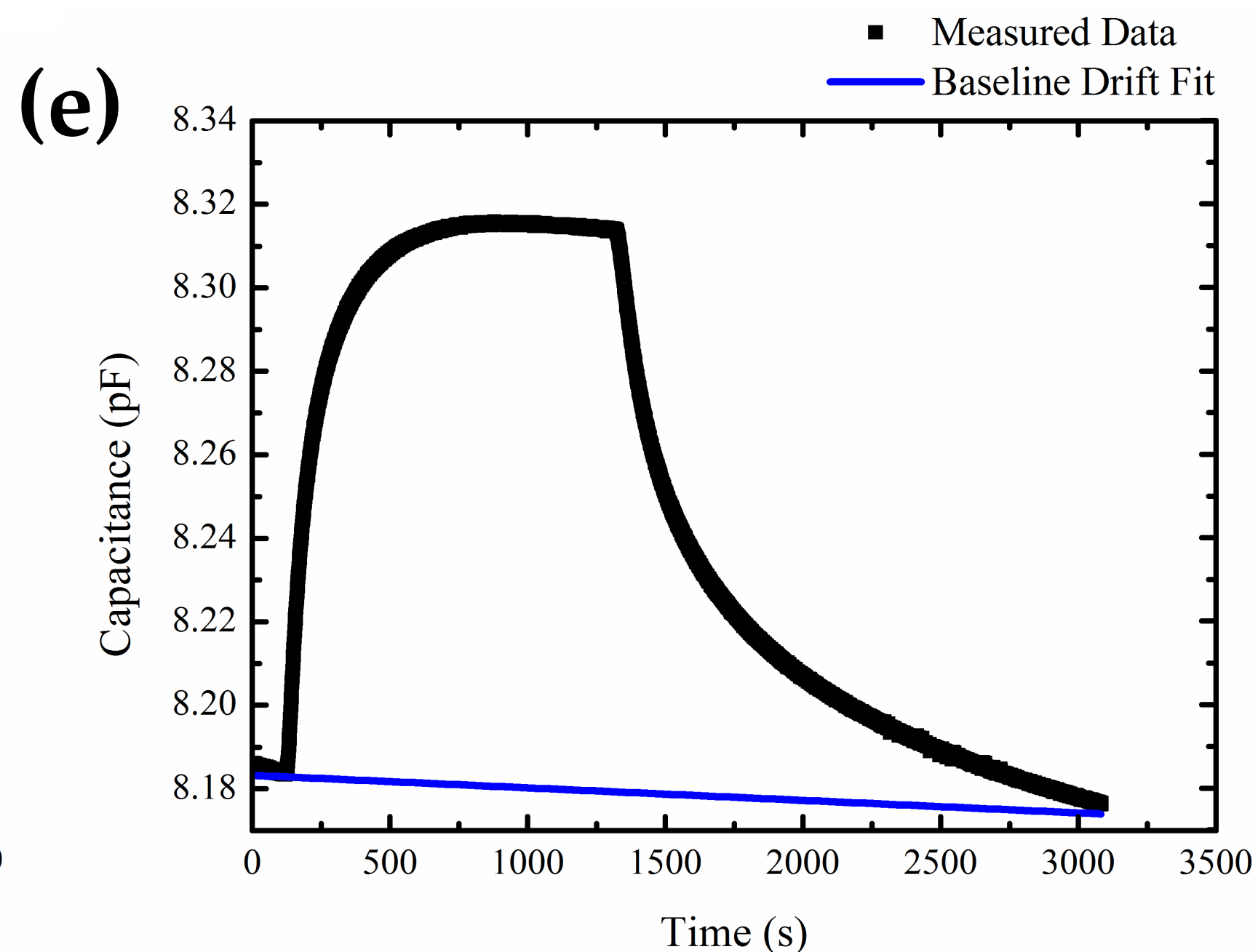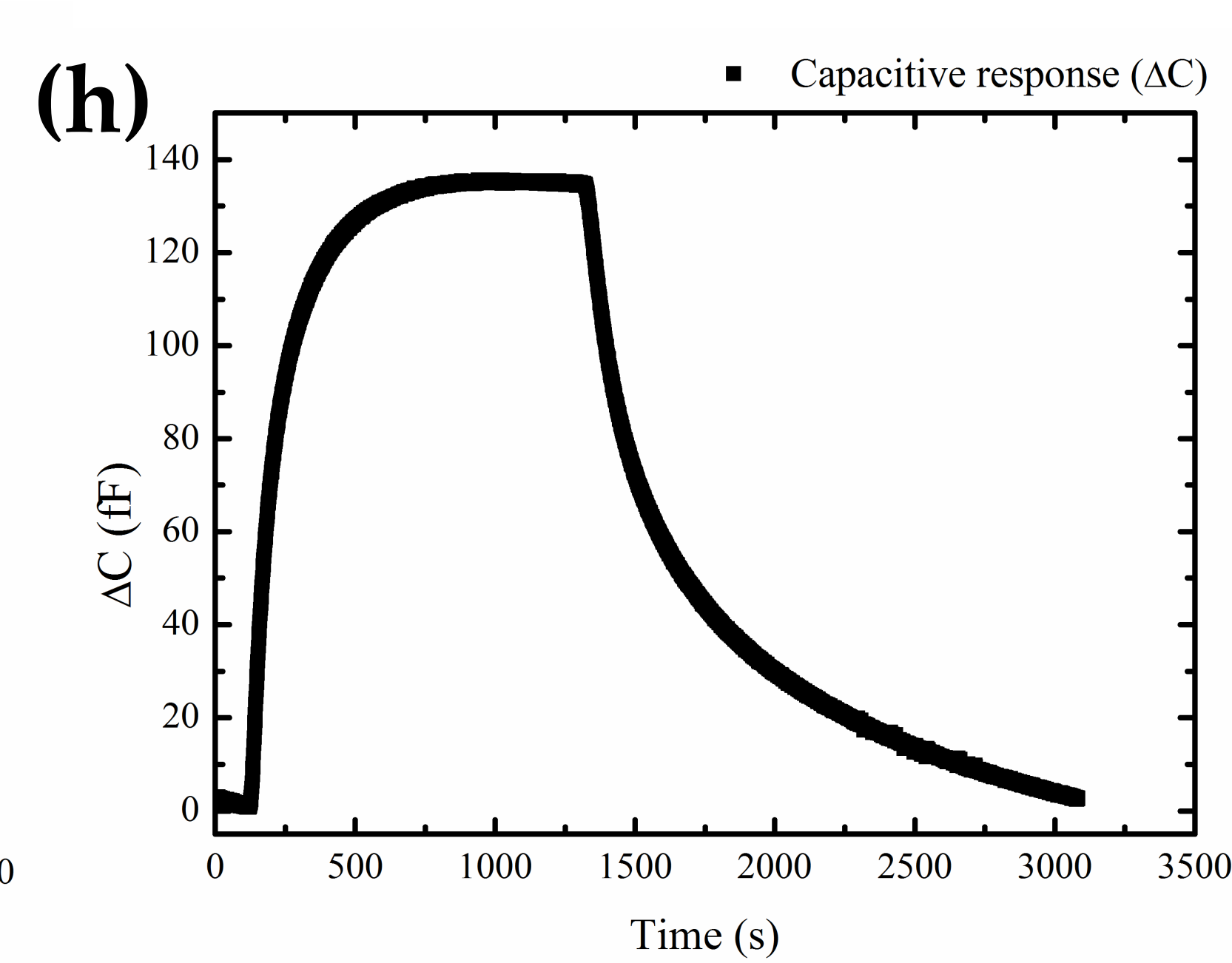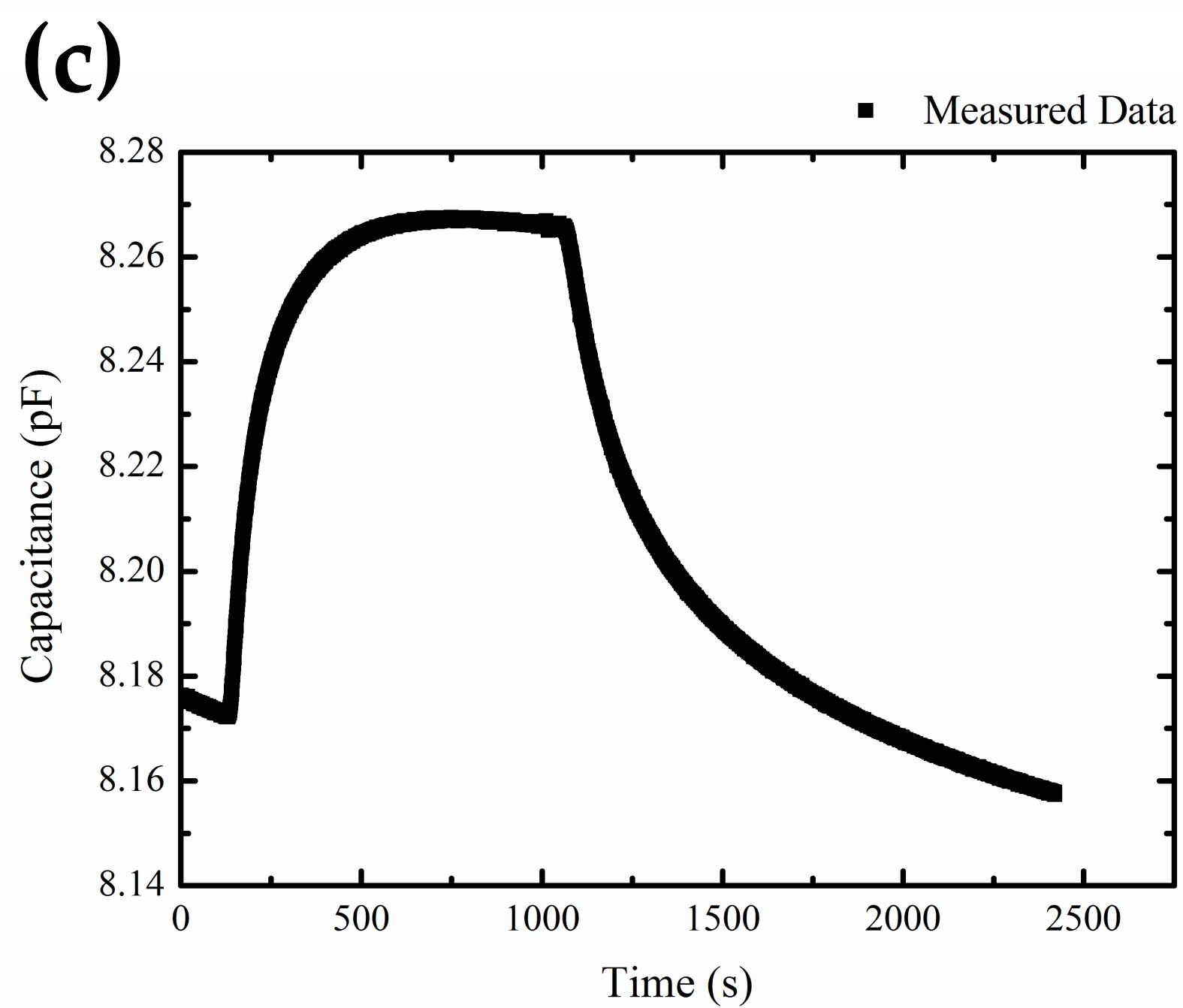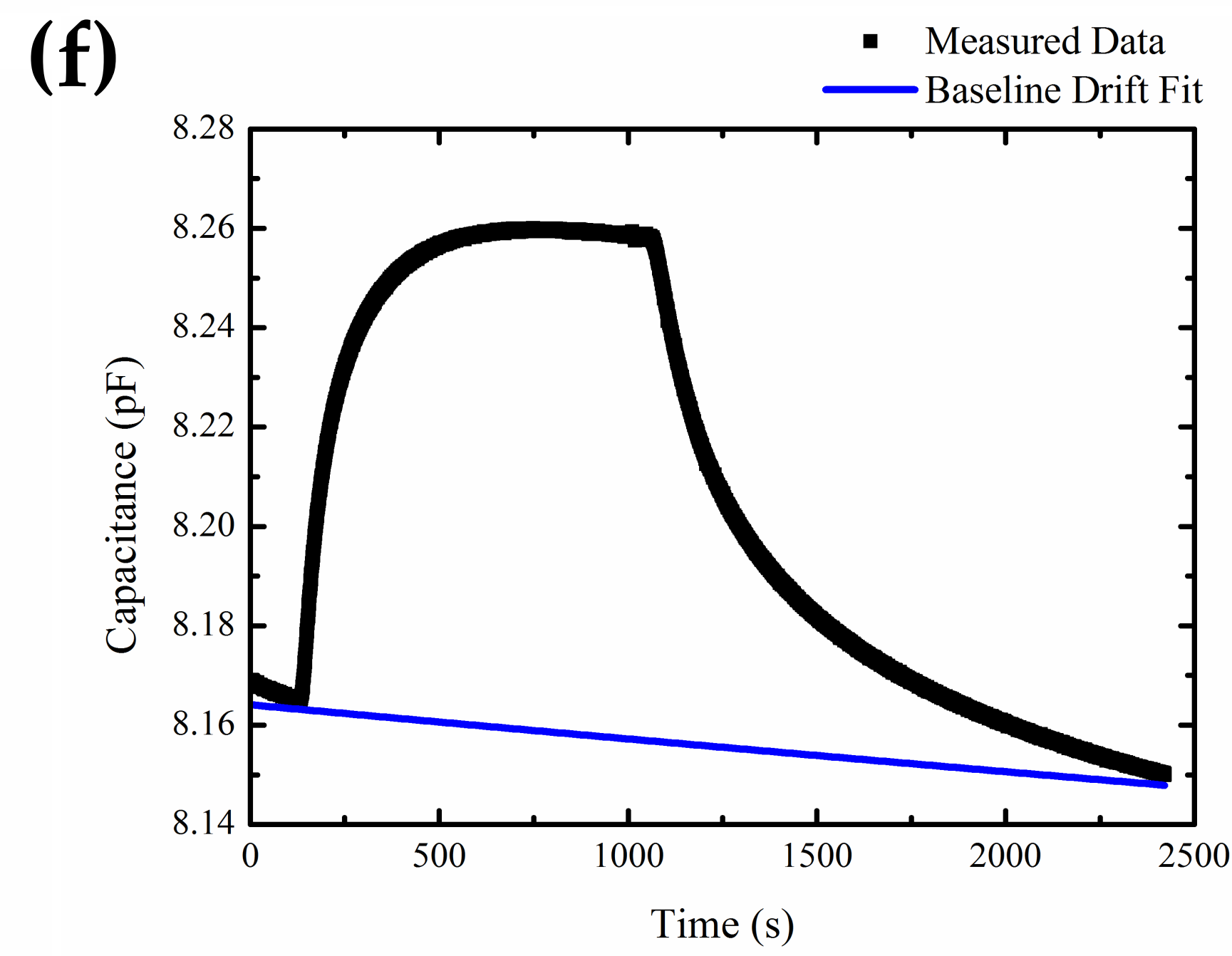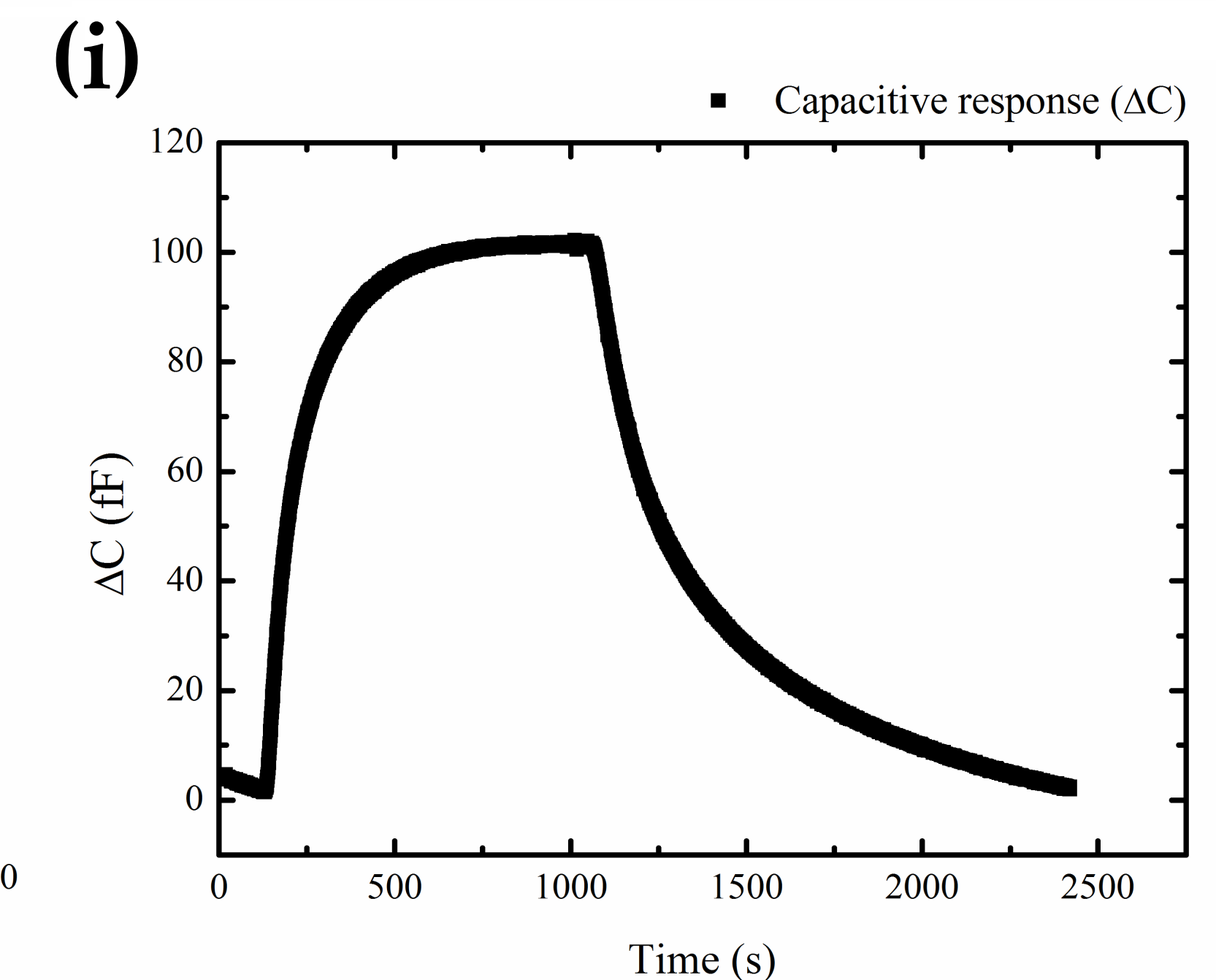

Supplement: Supplementary file 1 [file sensors-19-00888-s001.zip › Images/Fig_S19.pdf]

(a)

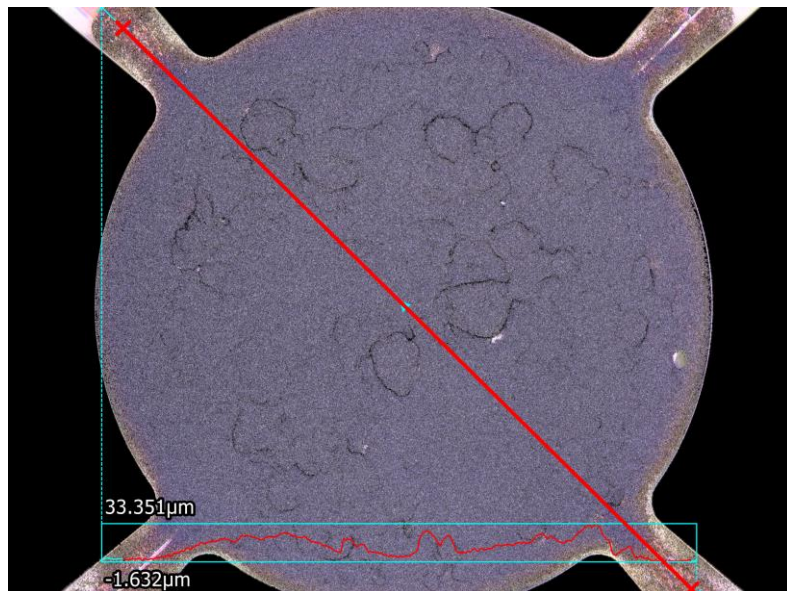

(b)

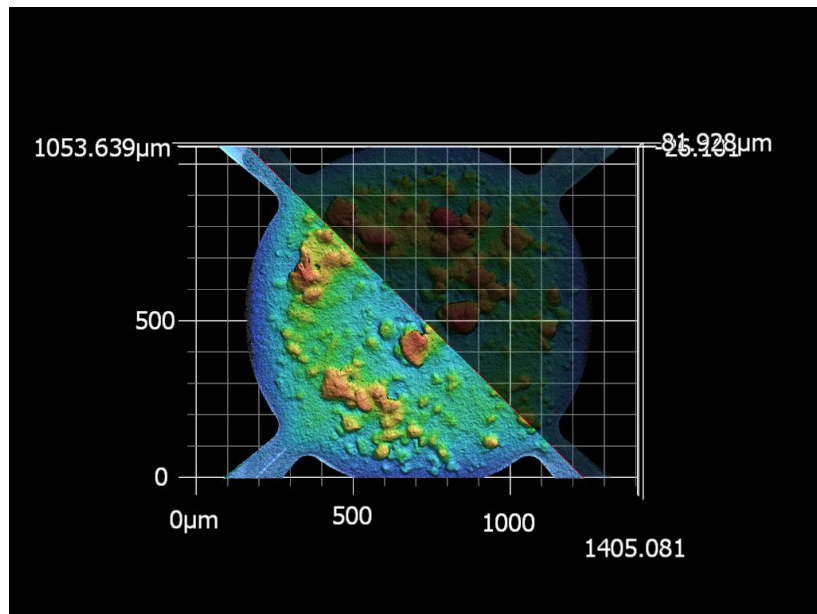

(c)

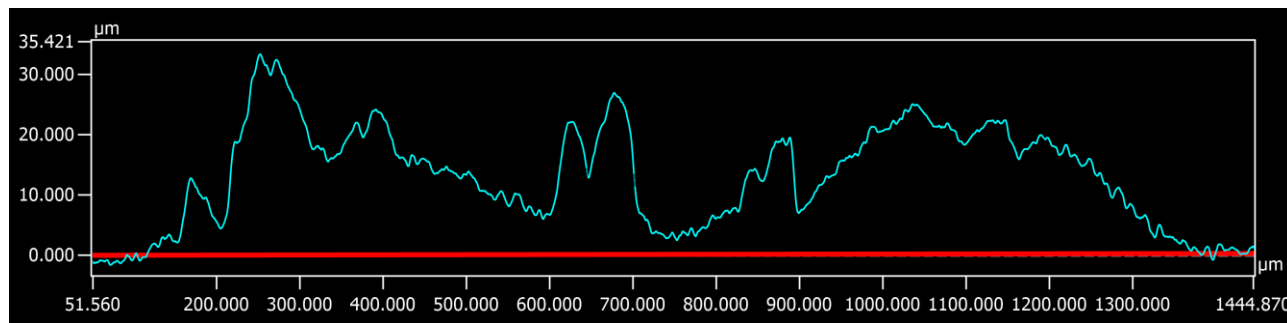

Supplement: Supplementary file 1 [file sensors-19-00888-s001.zip › Images/Fig_S2.pdf]

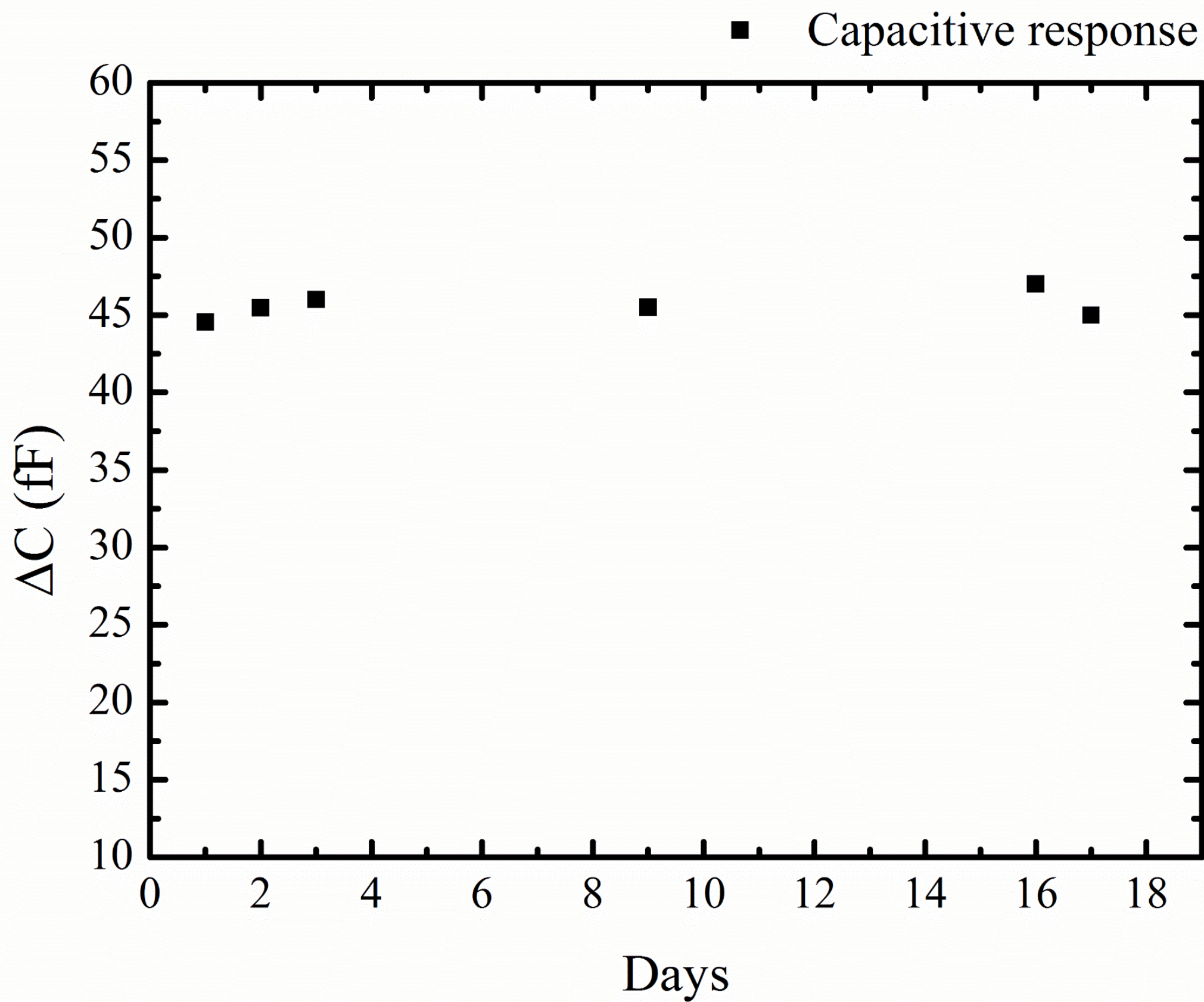

Supplement: Supplementary file 1 [file sensors-19-00888-s001.zip › Images/Fig_S20.pdf]

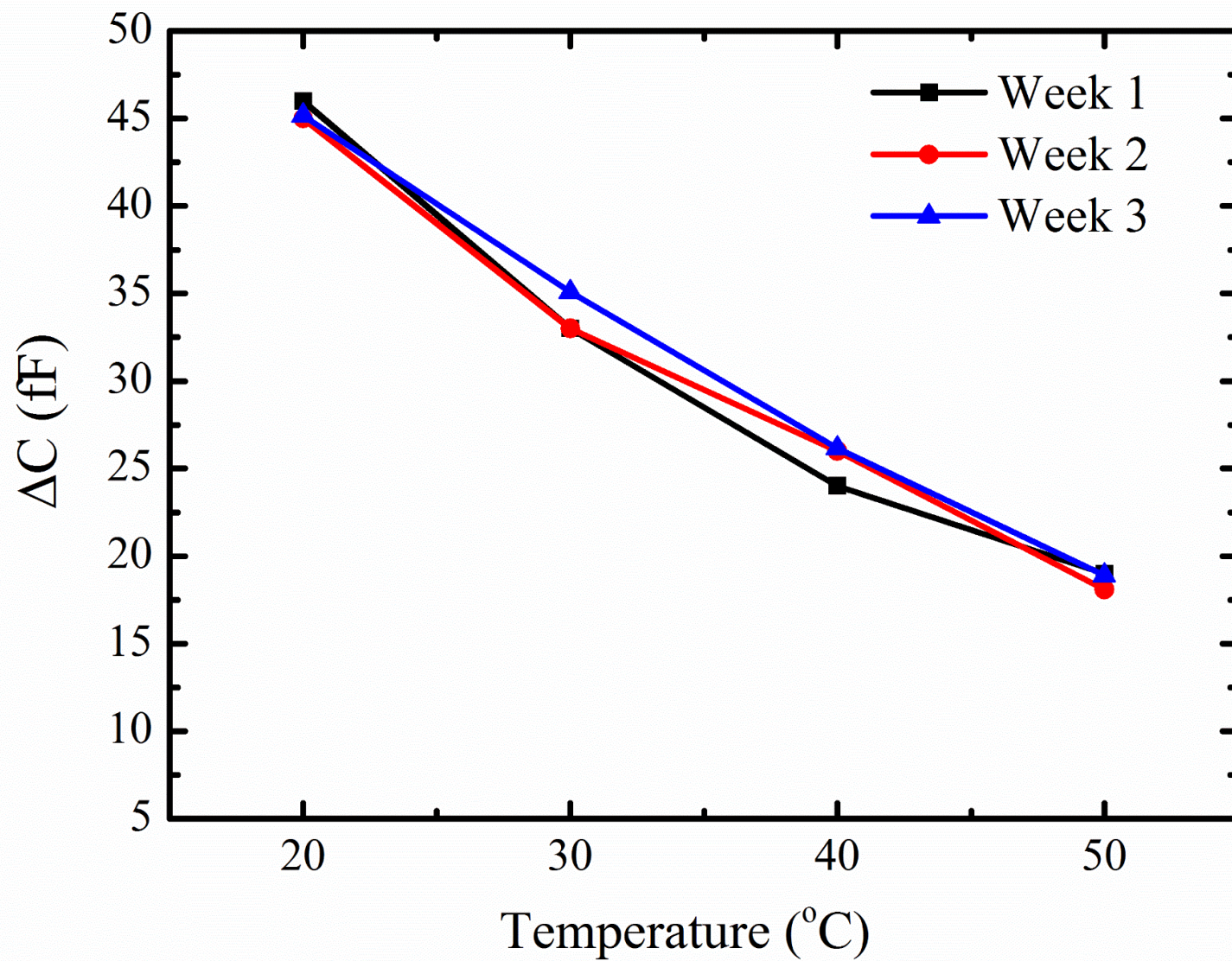

Supplement: Supplementary file 1 [file sensors-19-00888-s001.zip › Images/Fig_S21.pdf]

**(a)**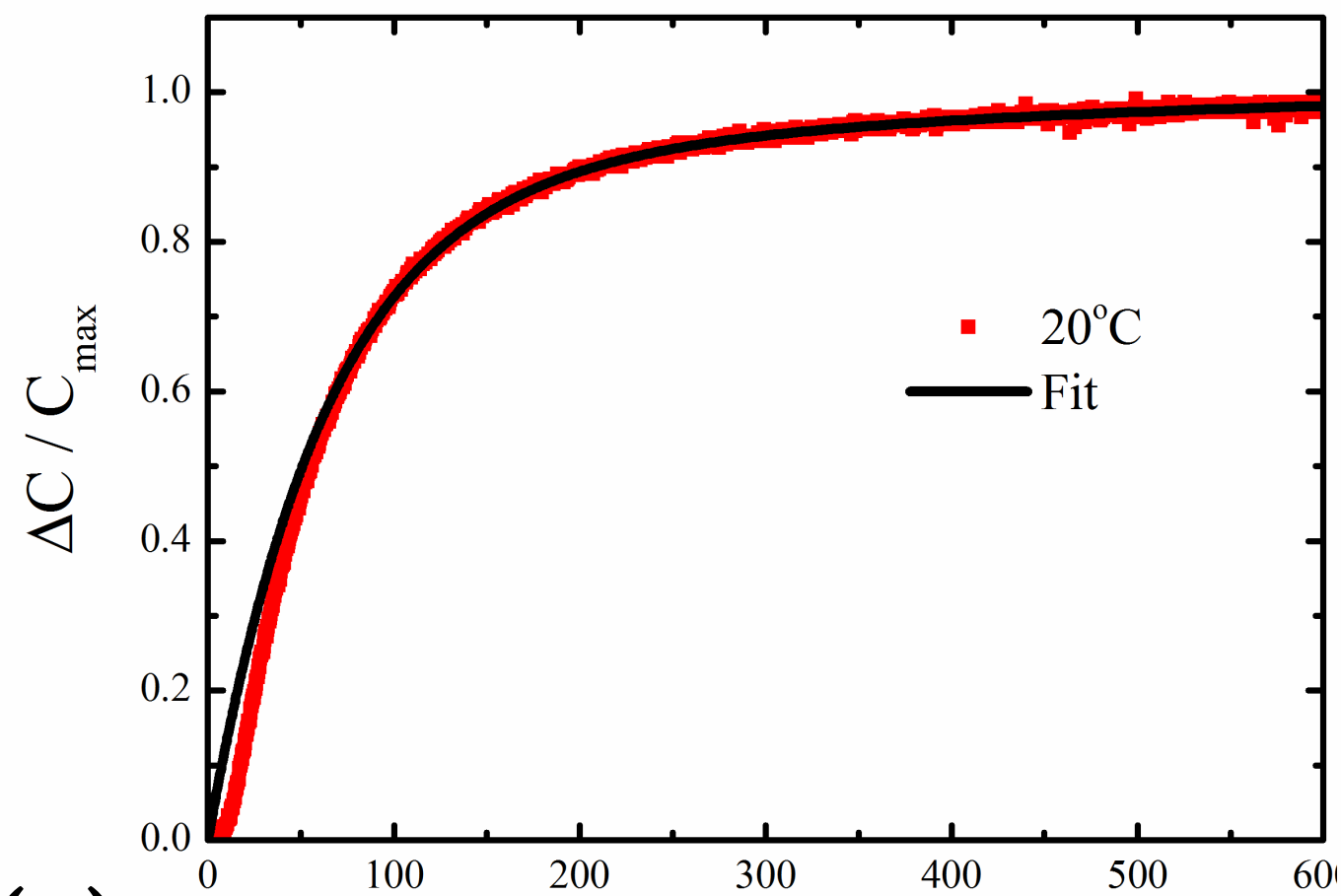**(b)**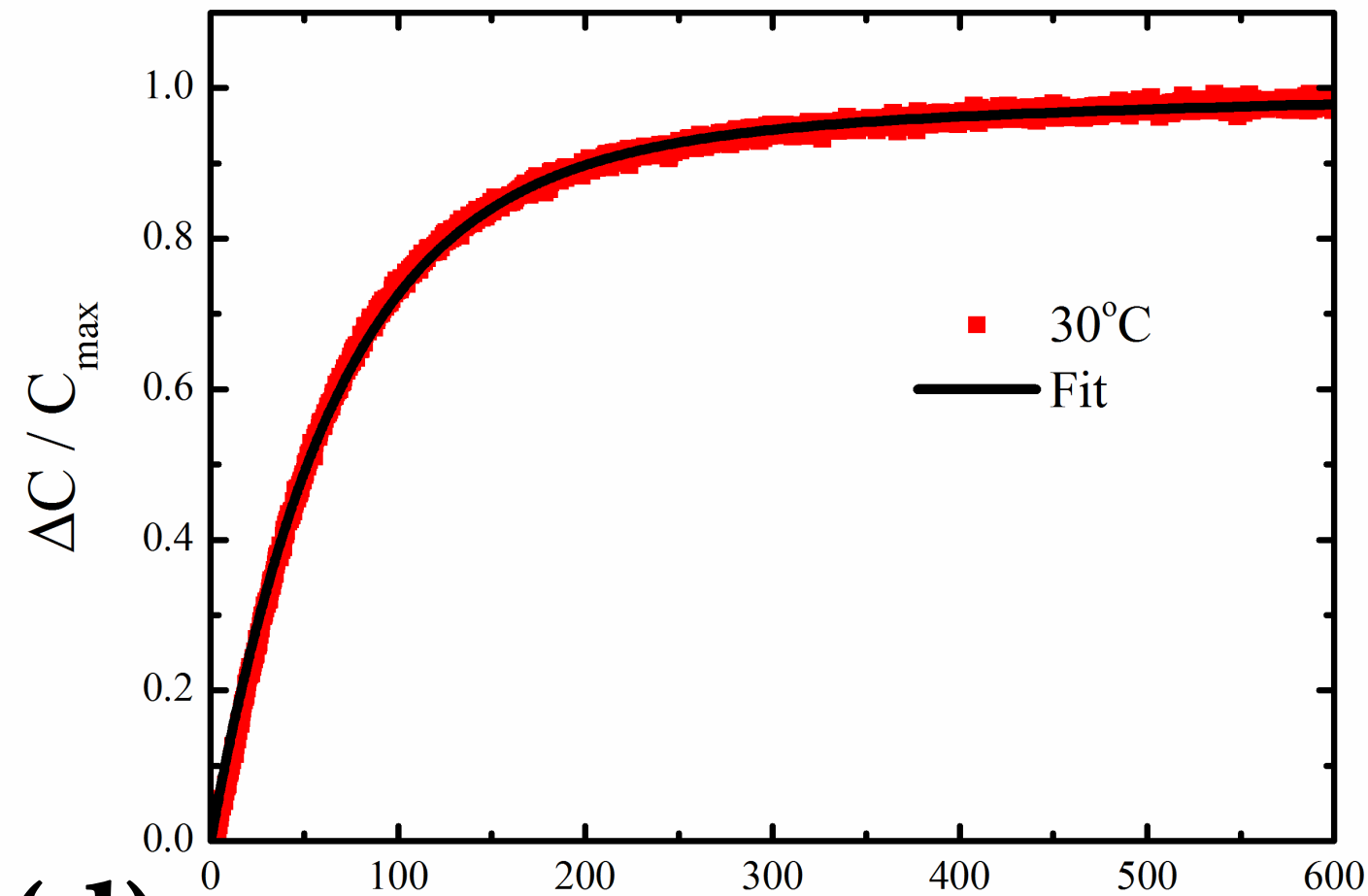**(c)**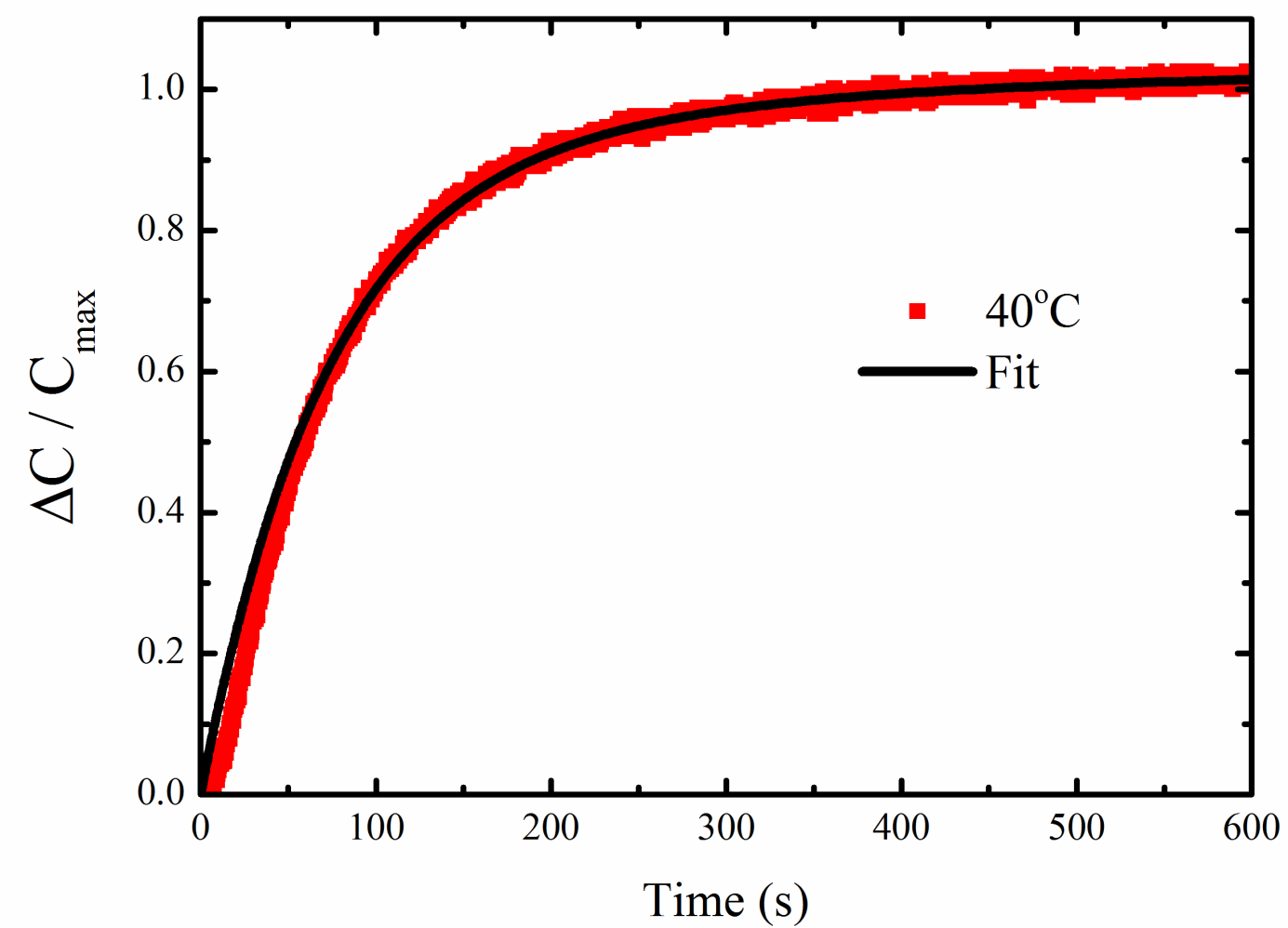**(d)**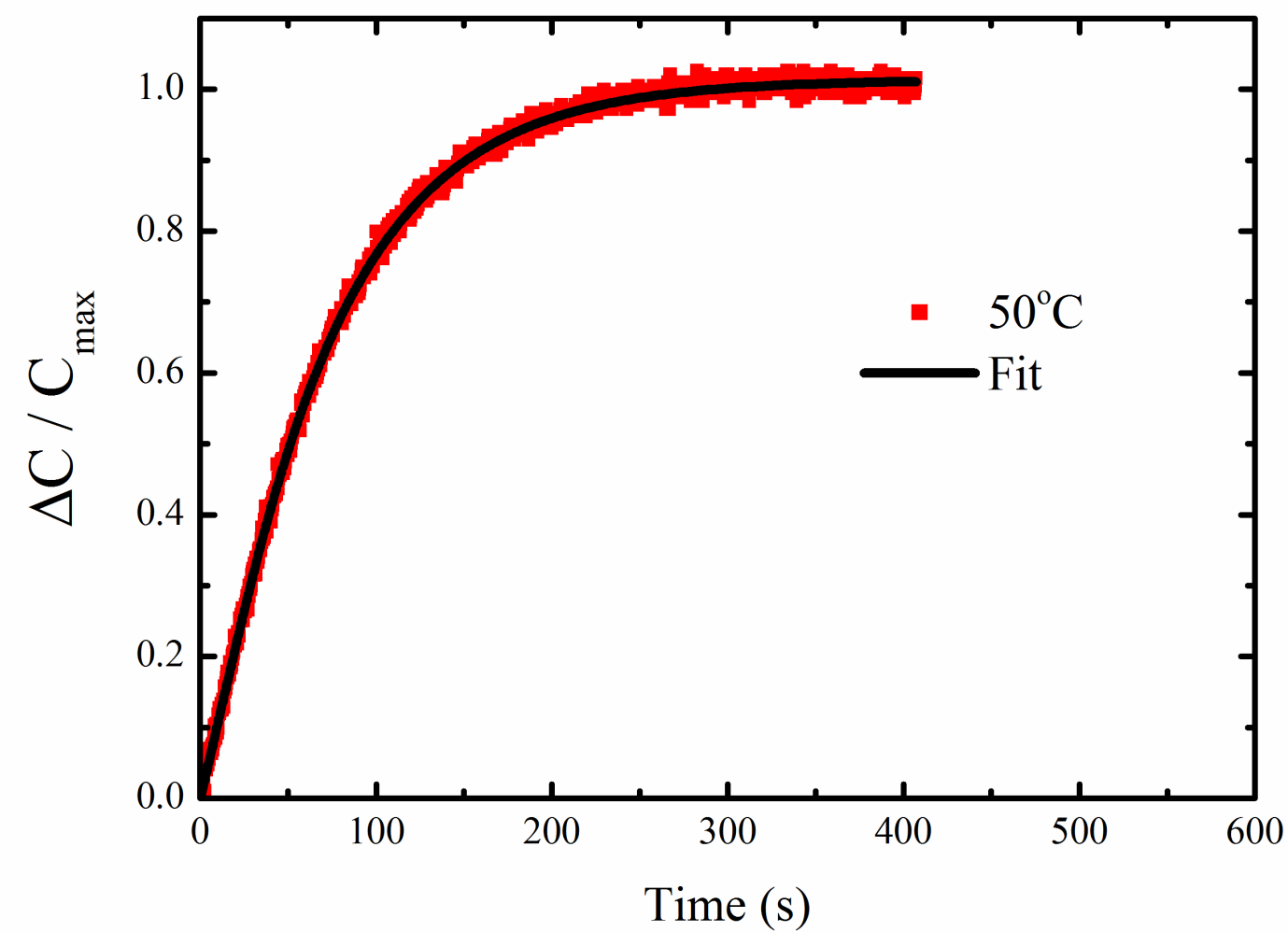

Supplement: Supplementary file 1 [file sensors-19-00888-s001.zip › Images/Fig_S22.pdf]

**(a)**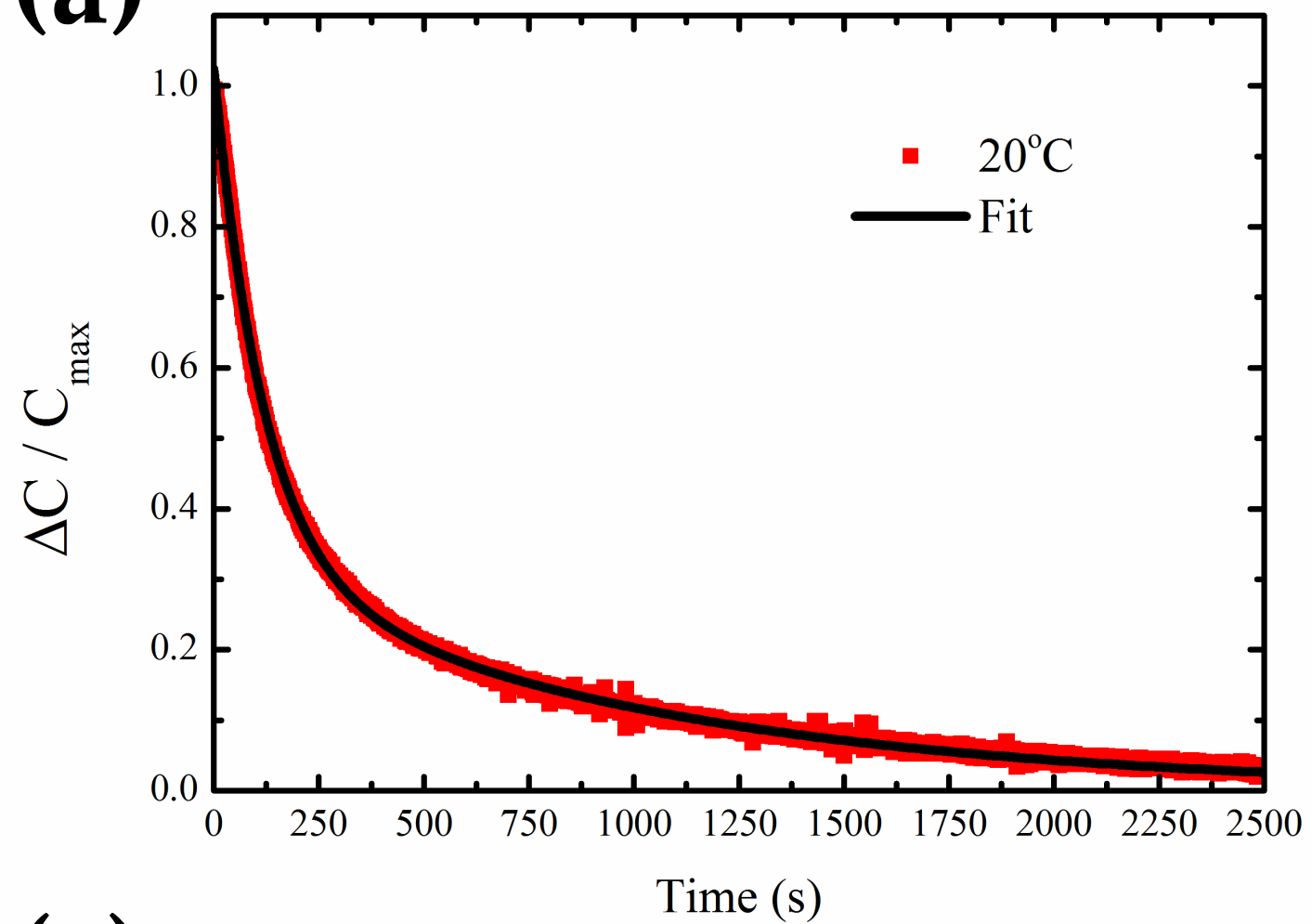**(b)**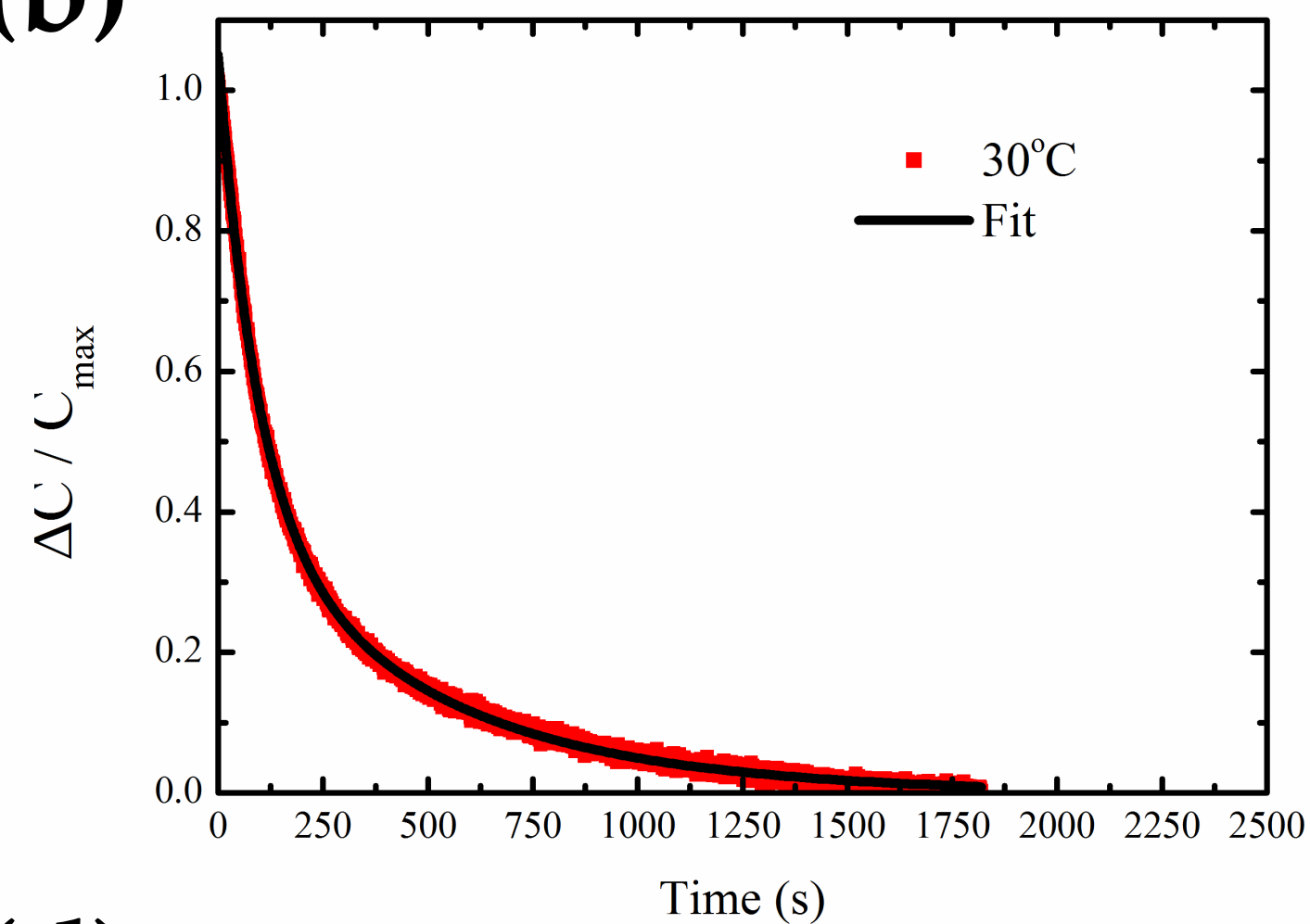**(c)**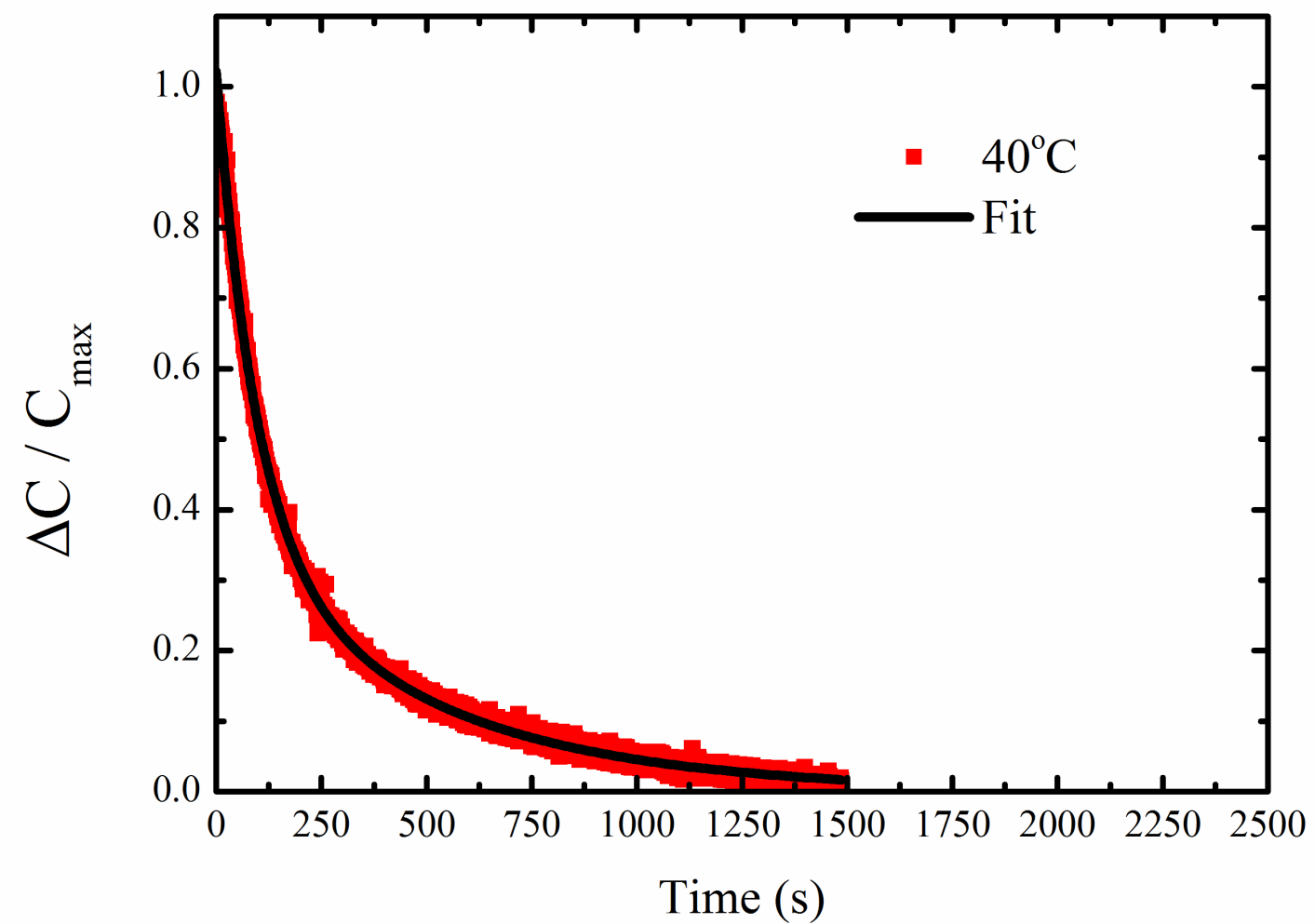**(d)**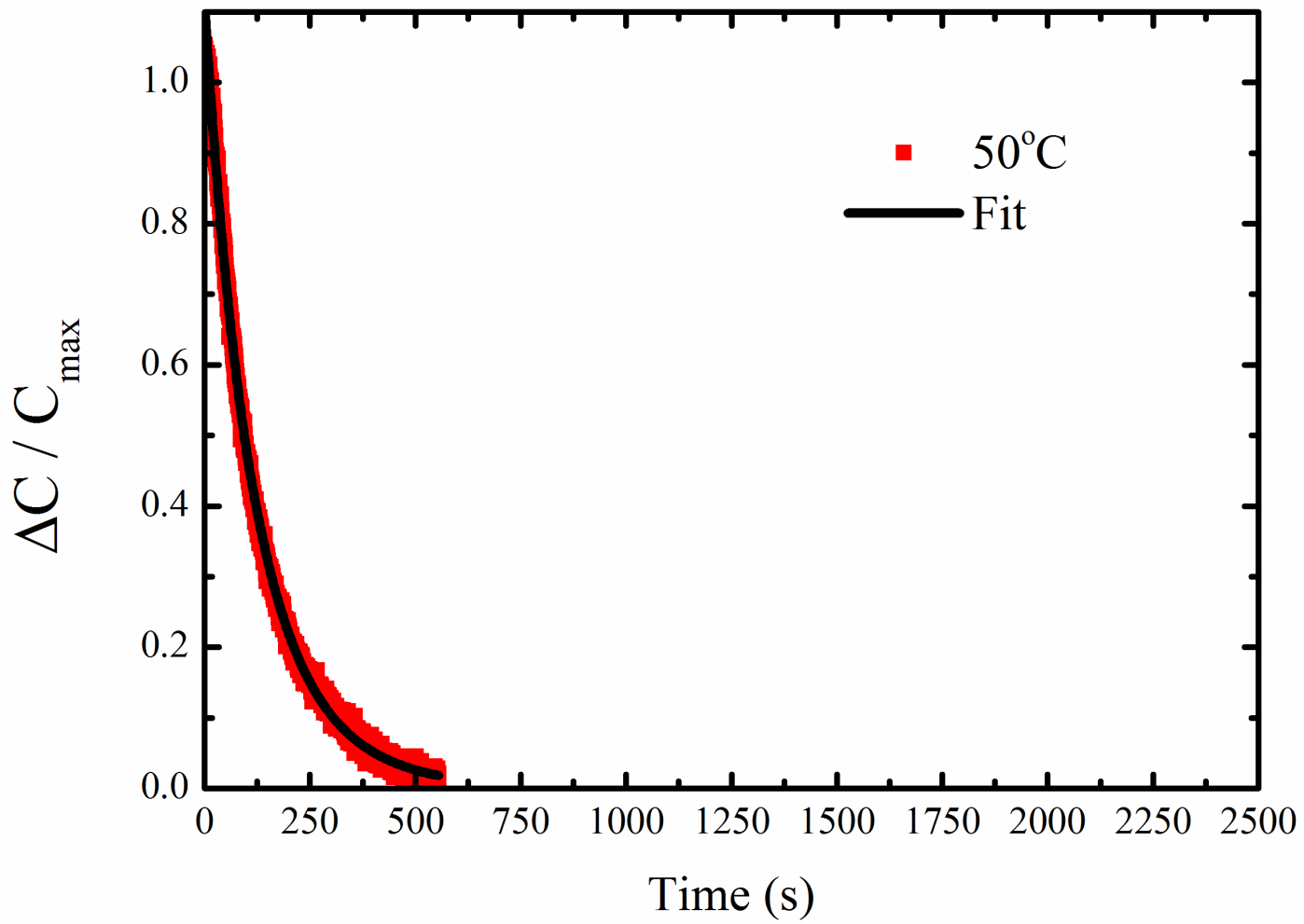

Supplement: Supplementary file 1 [file sensors-19-00888-s001.zip › Images/Fig_S23.pdf]

Power at 200°C

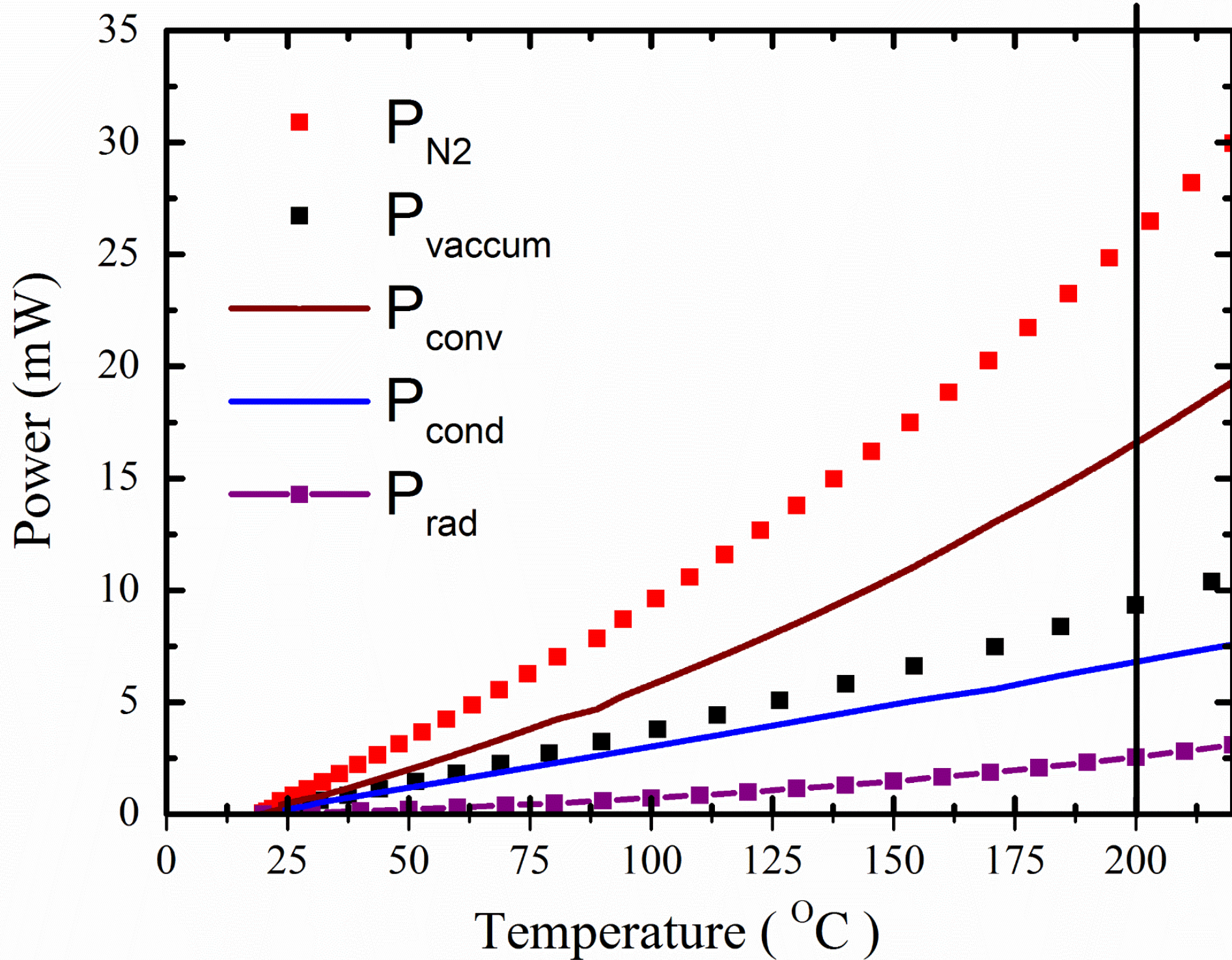

Supplement: Supplementary file 1 [file sensors-19-00888-s001.zip › Images/Fig_S3.pdf]

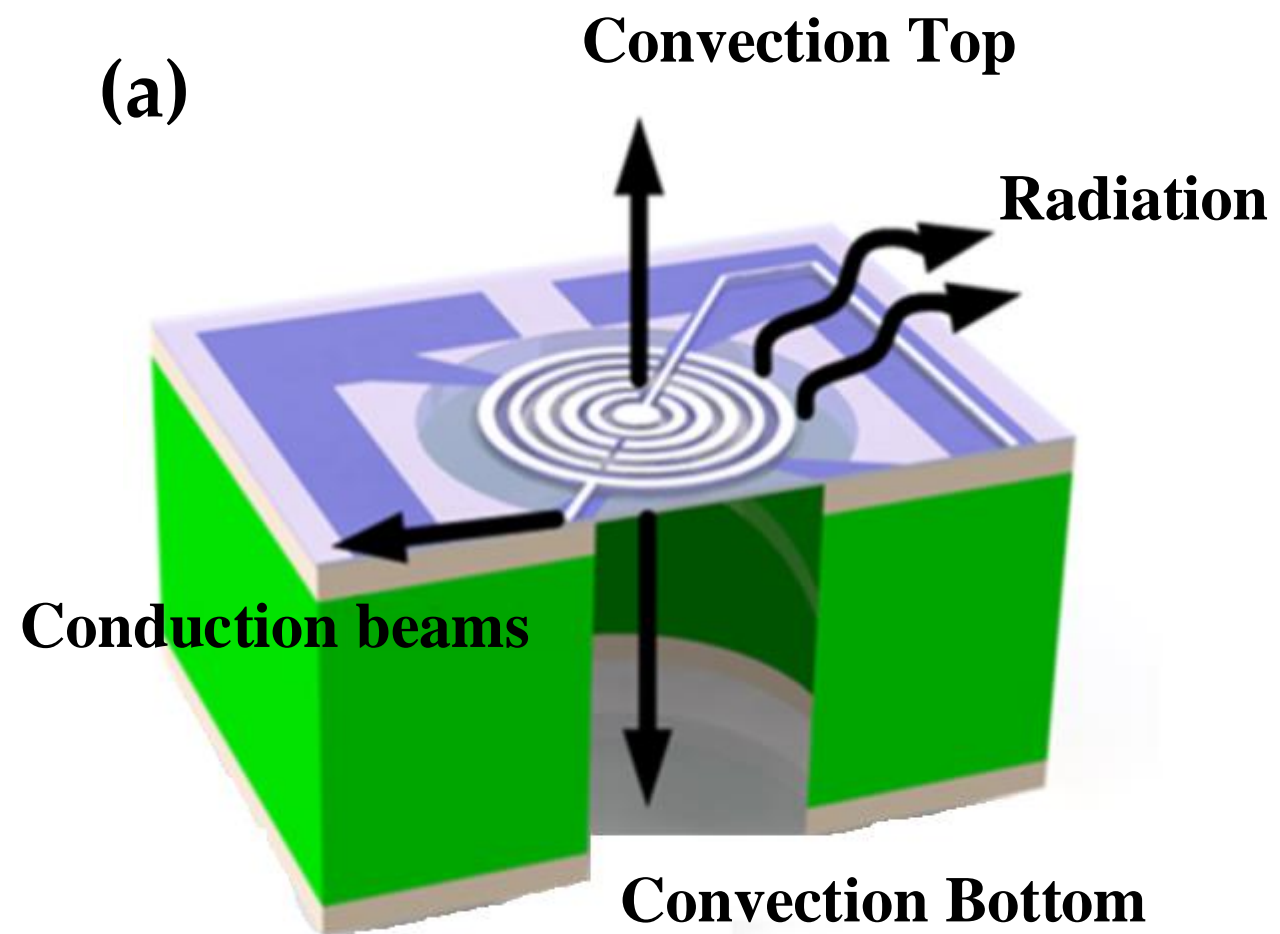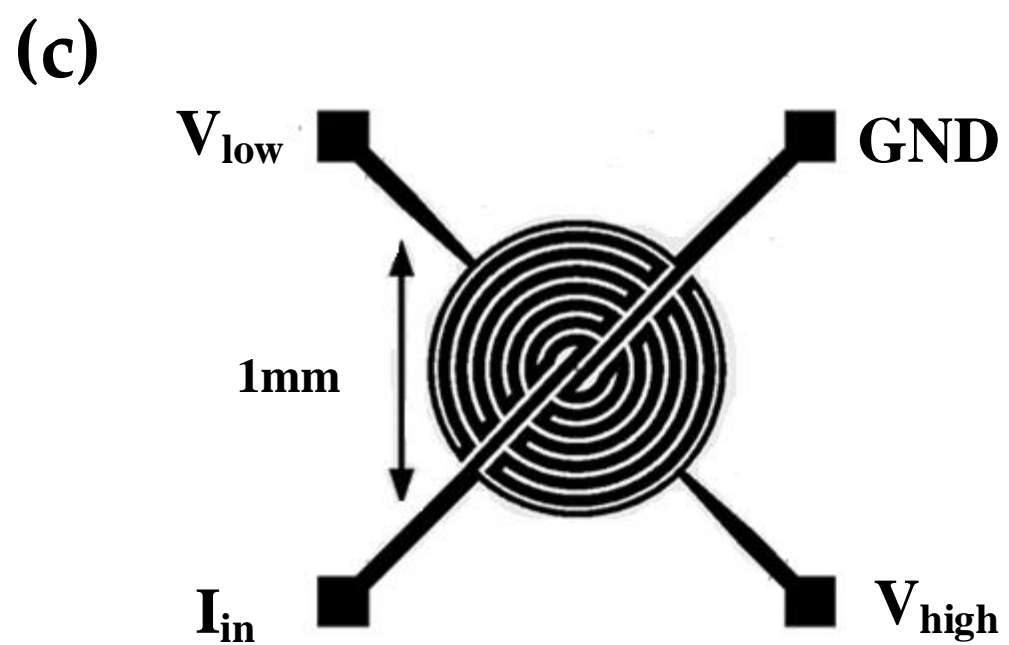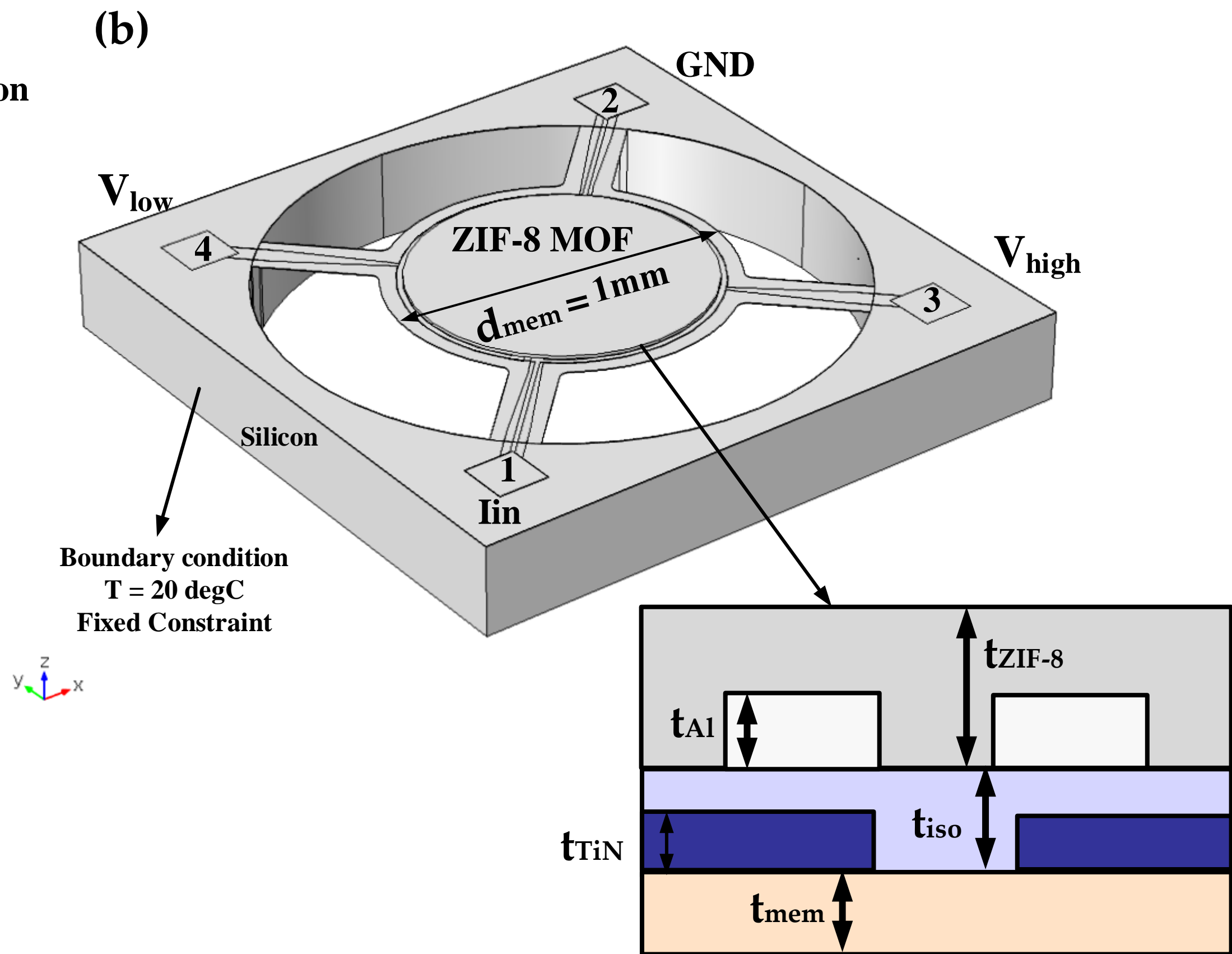

Supplement: Supplementary file 1 [file sensors-19-00888-s001.zip › Images/Fig_S4.pdf]

(a)

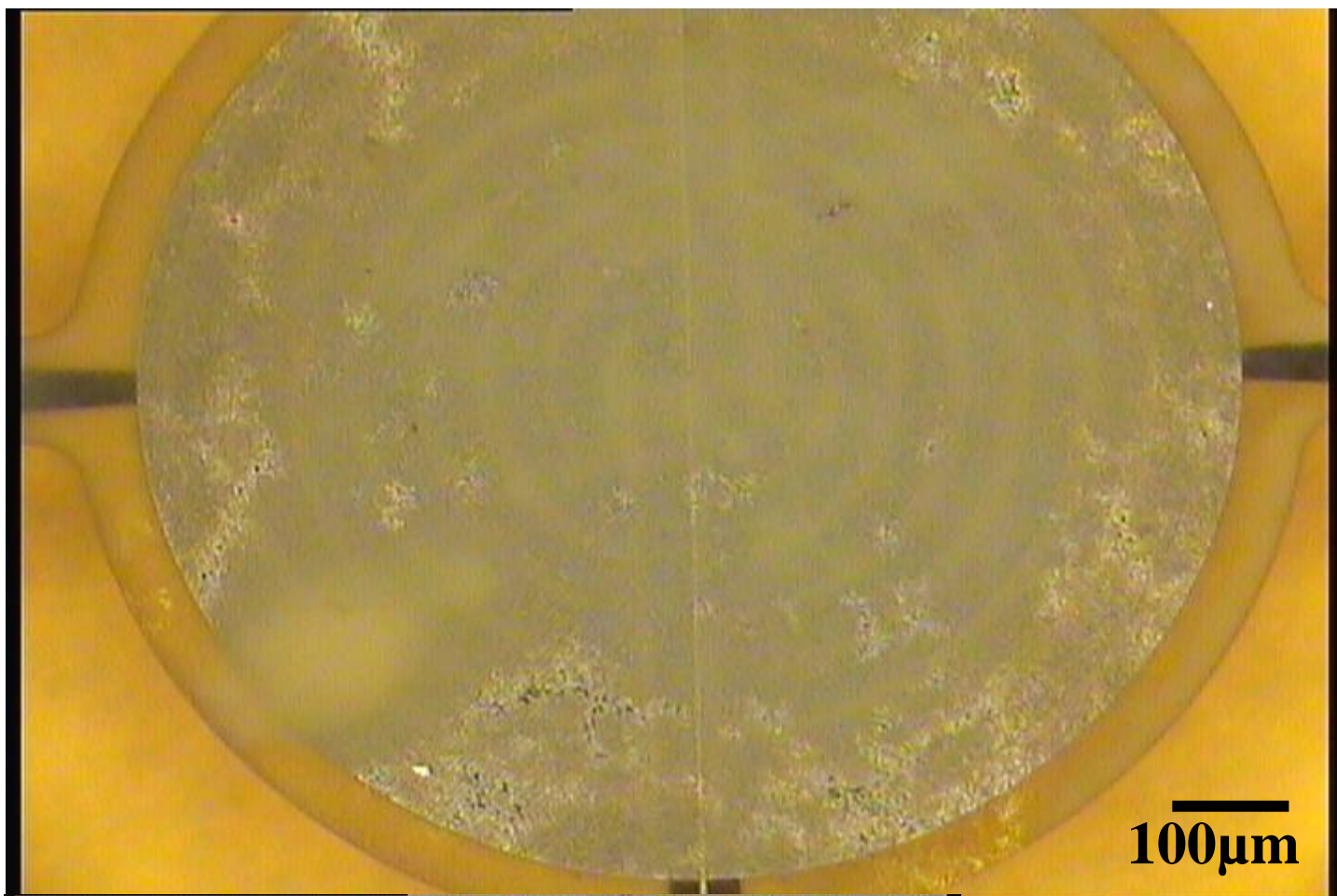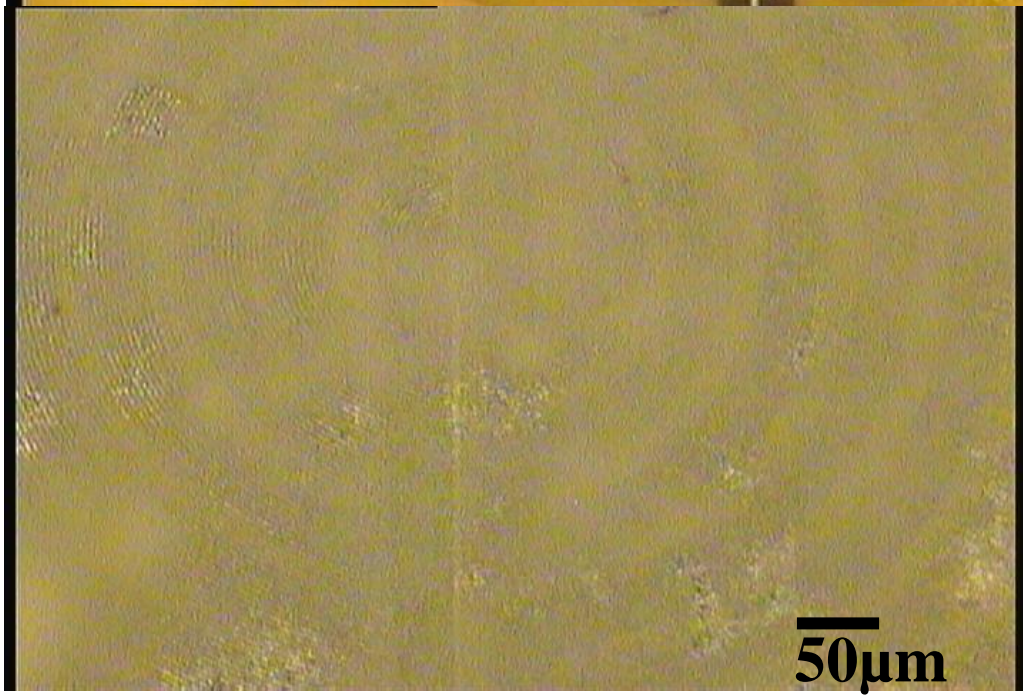

(b)

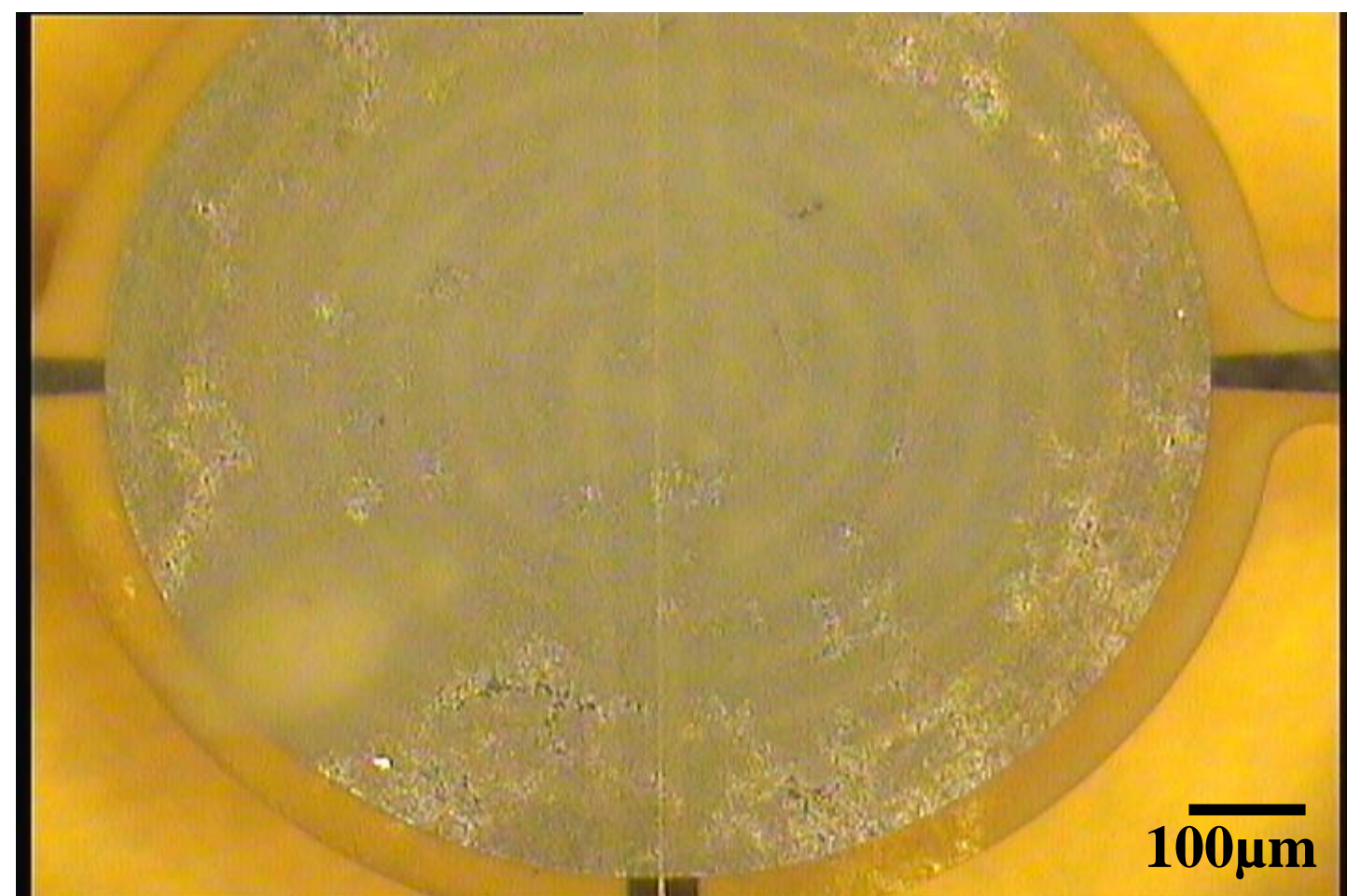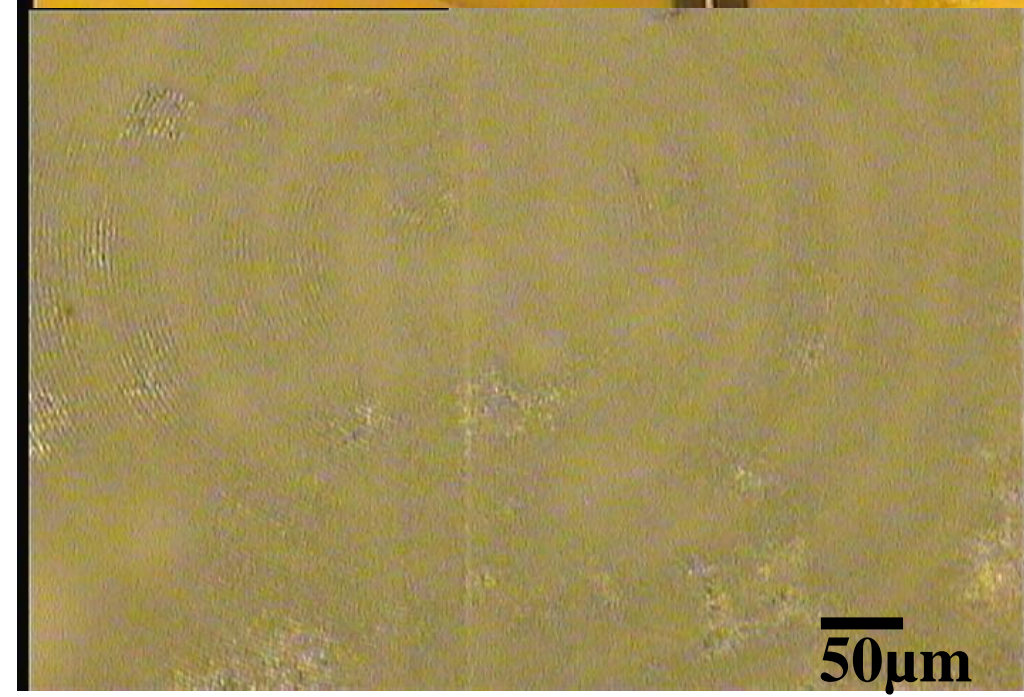

(c)

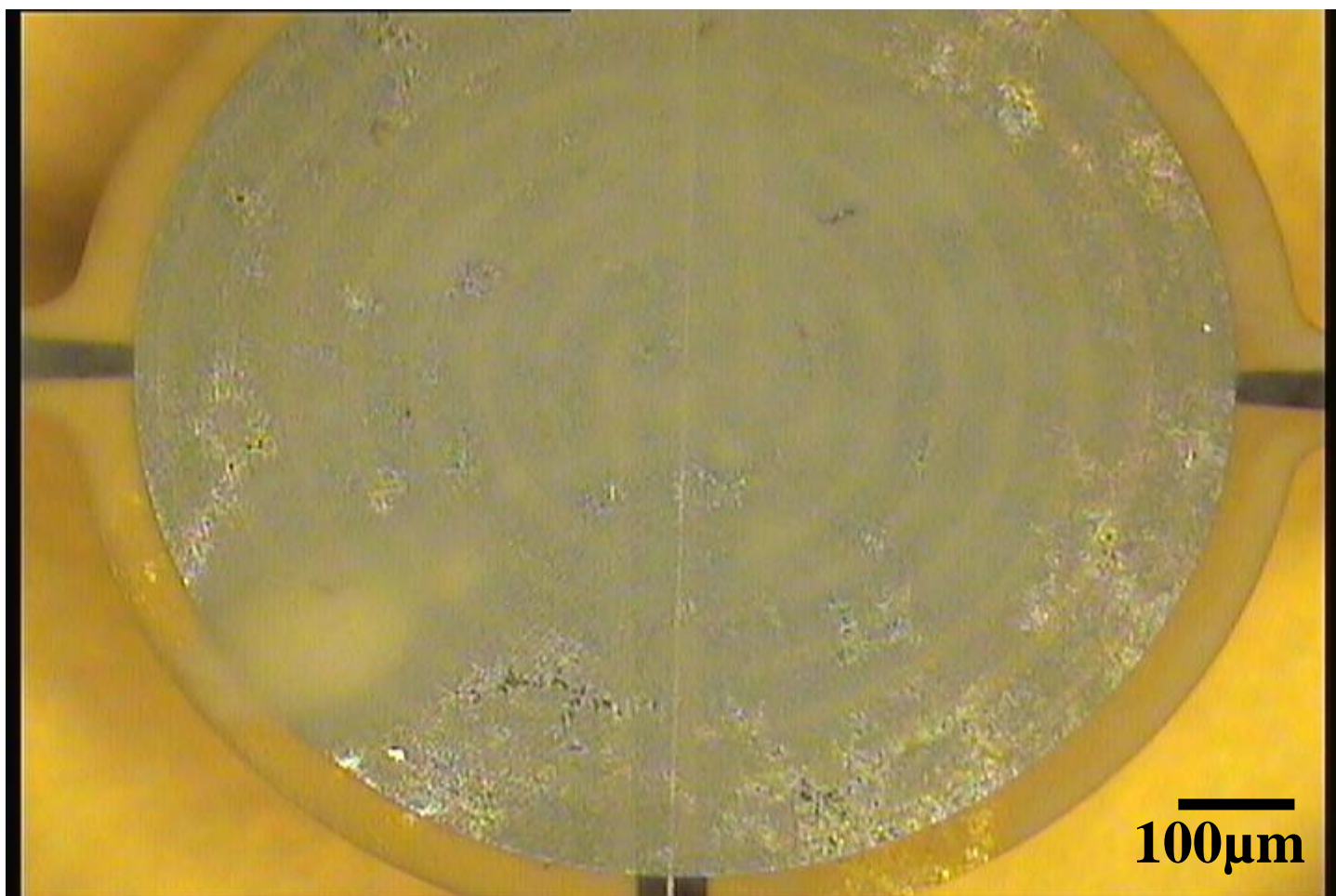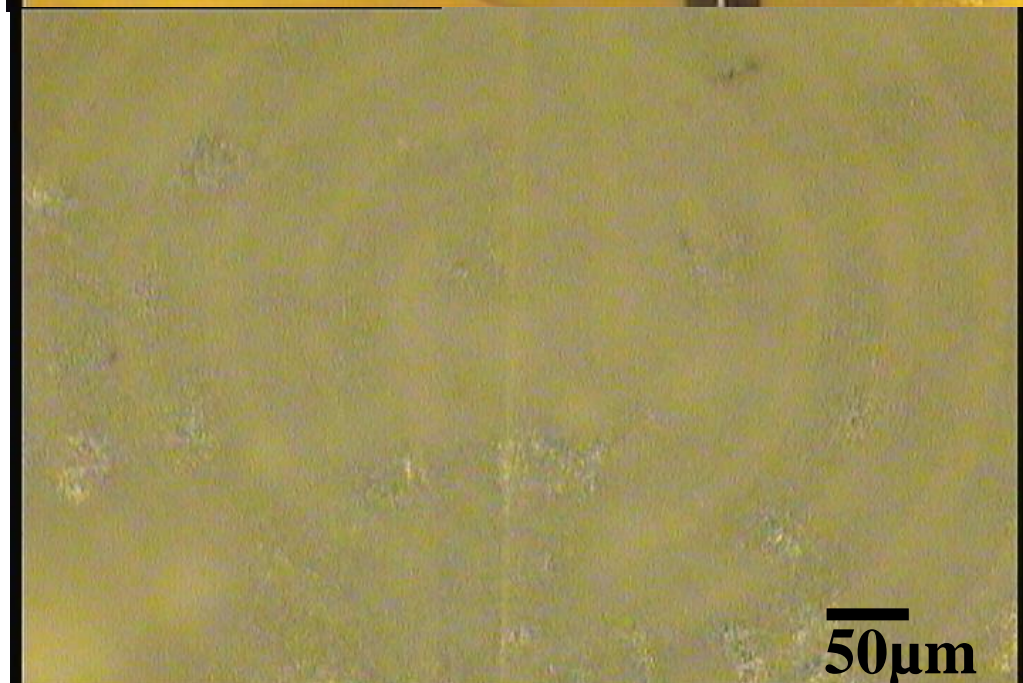

(d)

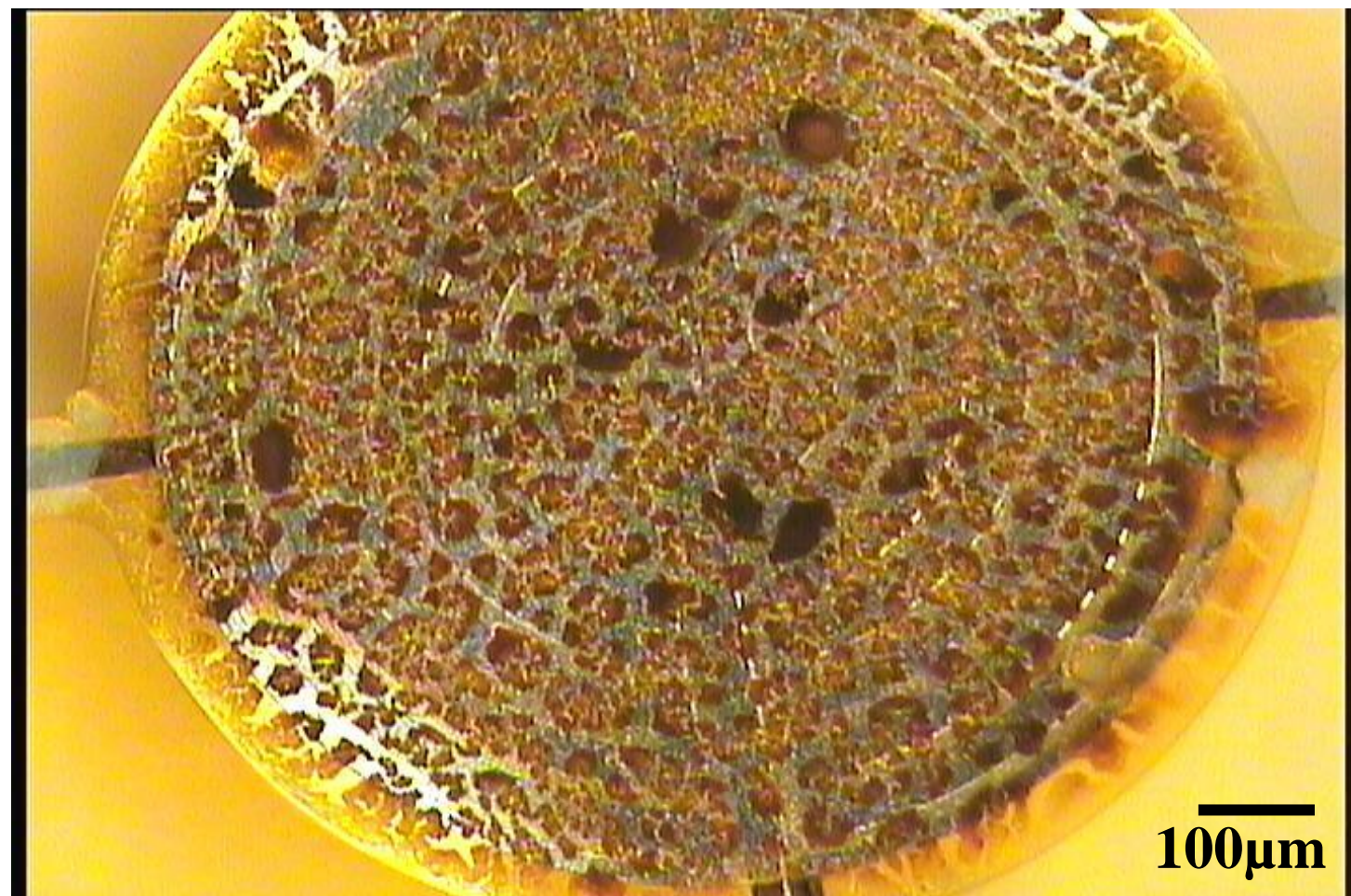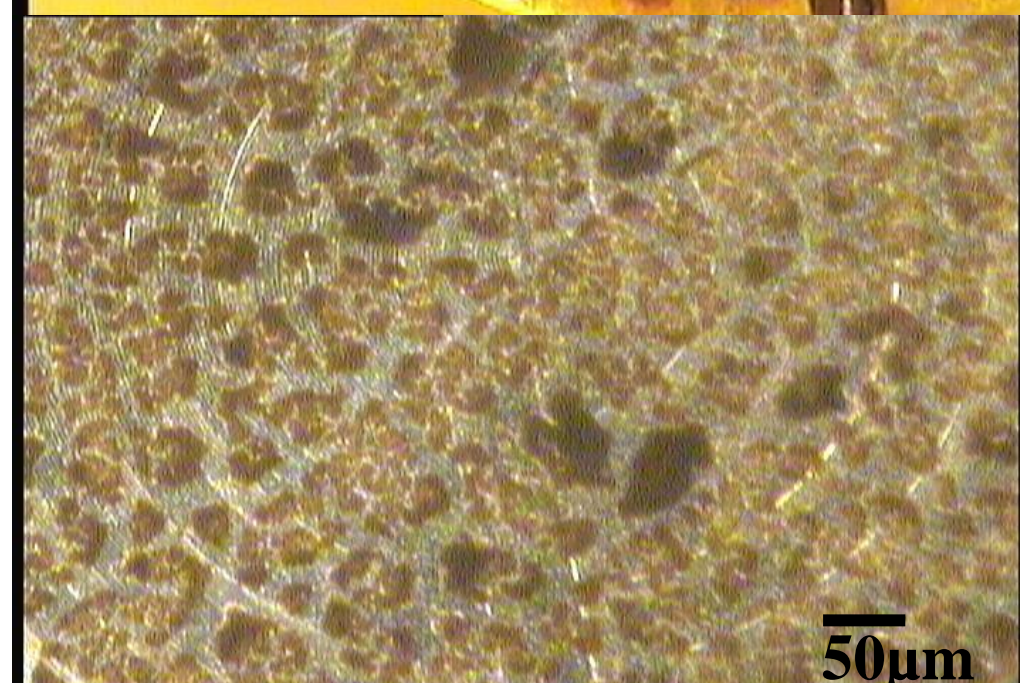

Supplement: Supplementary file 1 [file sensors-19-00888-s001.zip › Images/Fig_S5.pdf]

**(a)**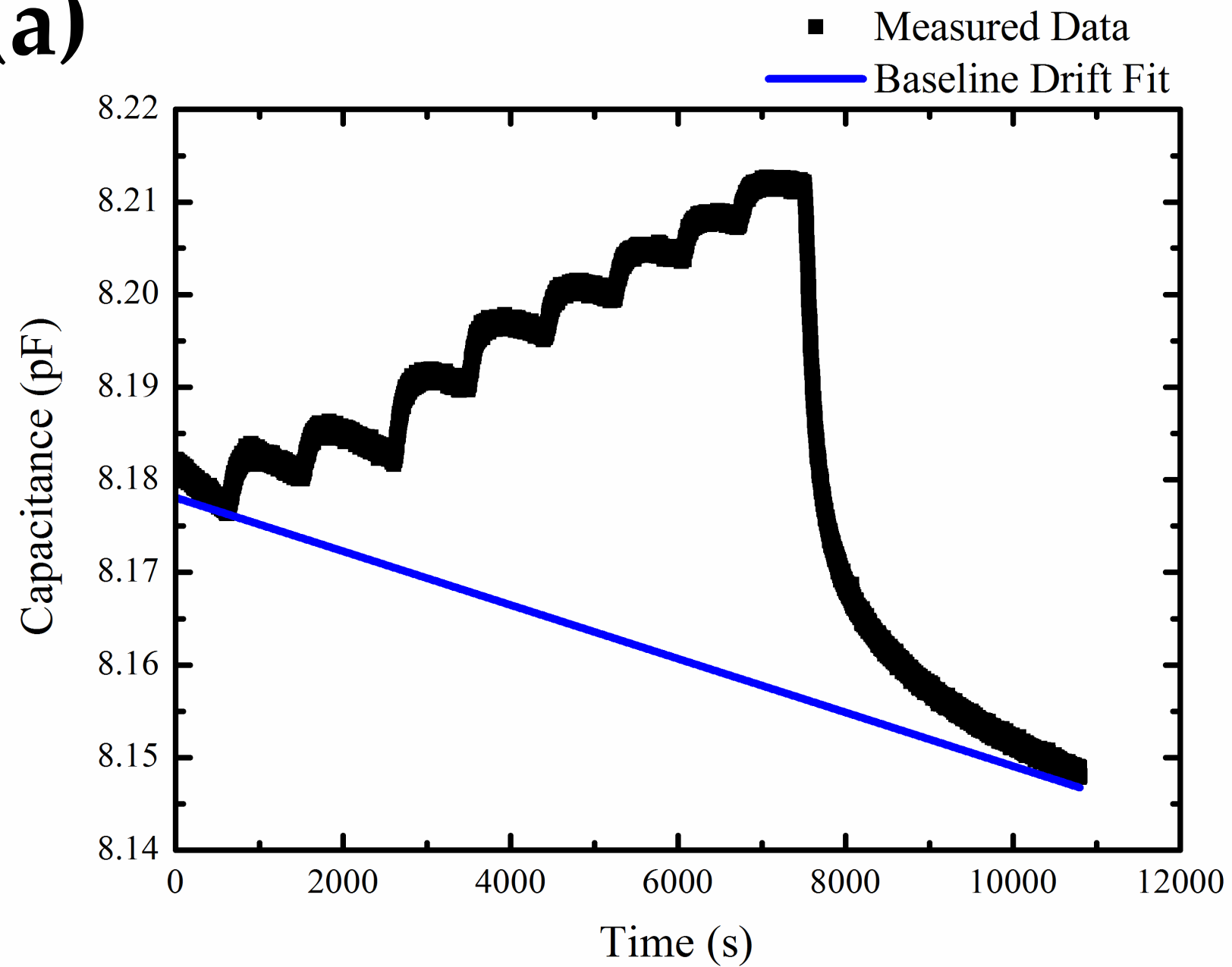**(b)**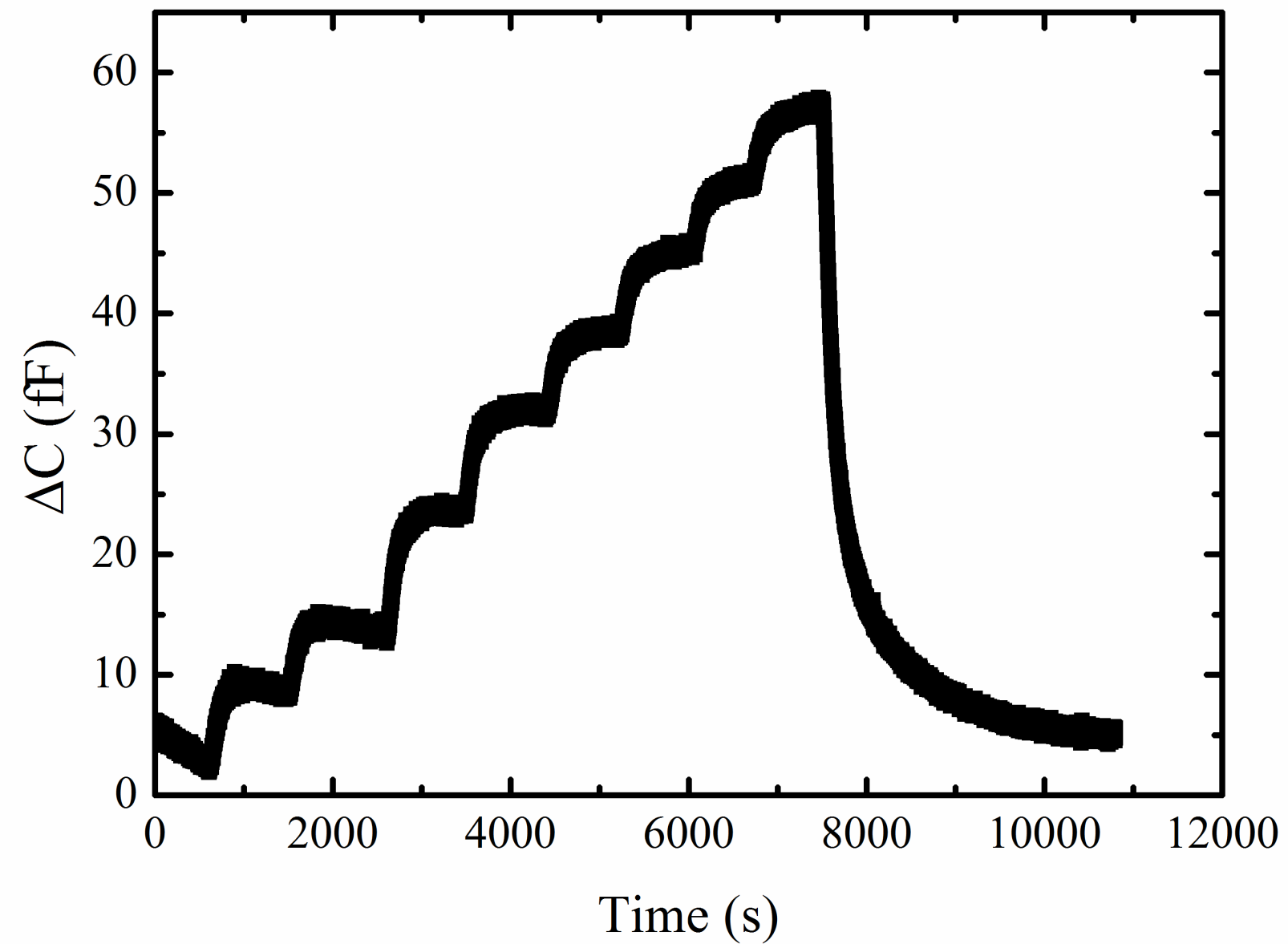

Supplement: Supplementary file 1 [file sensors-19-00888-s001.zip › Images/Fig_S6.pdf]

**(a)**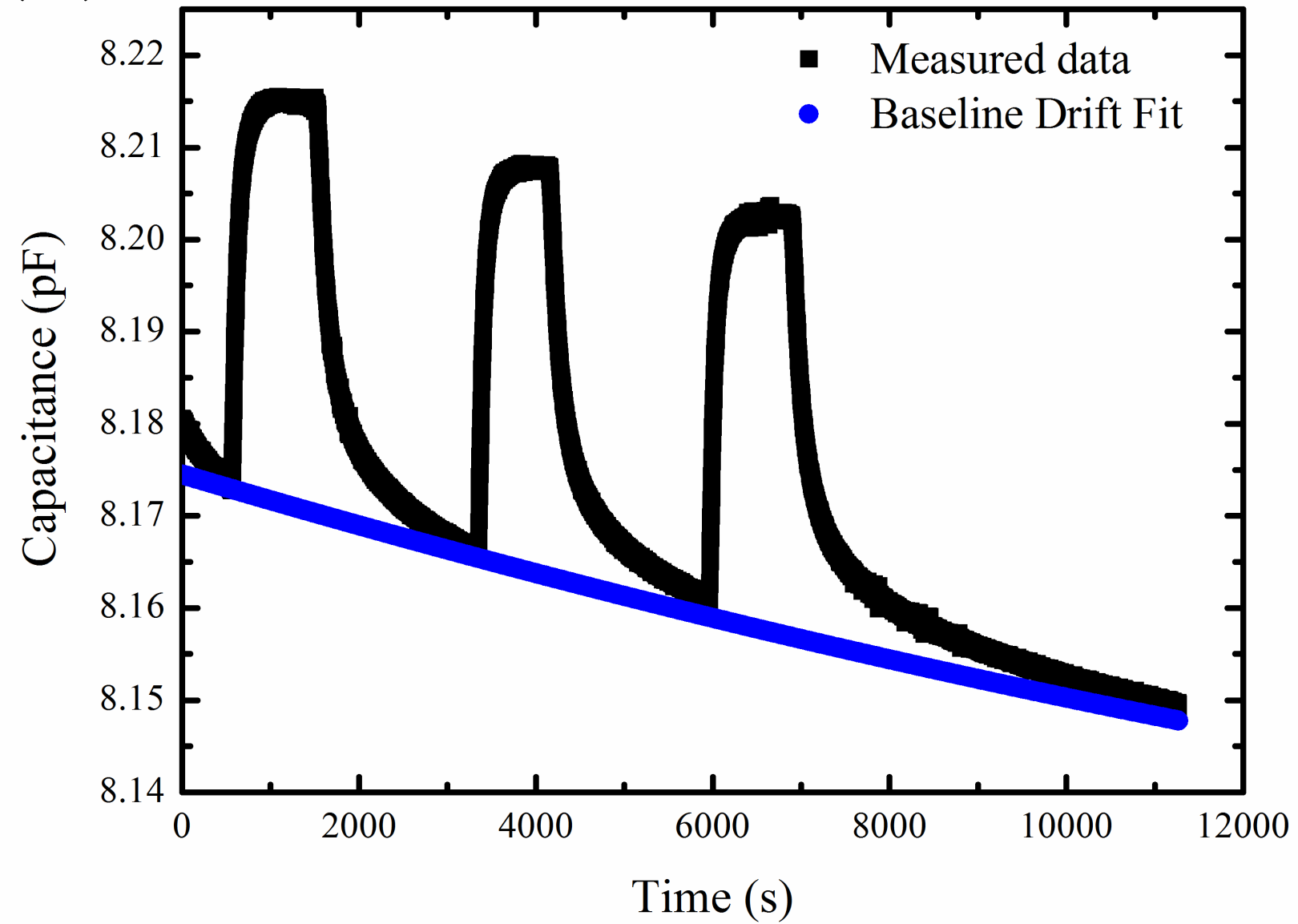**(b)**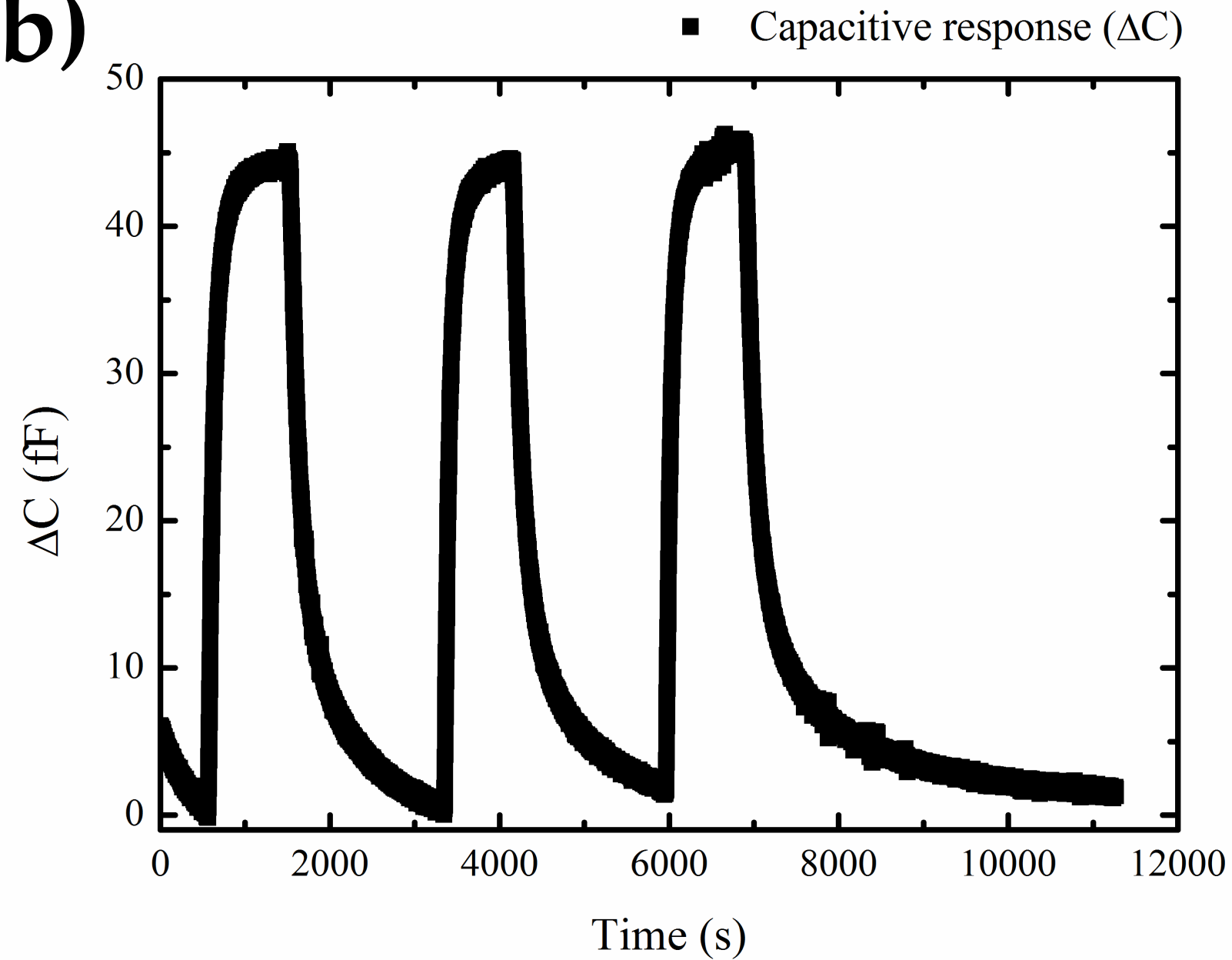

Supplement: Supplementary file 1 [file sensors-19-00888-s001.zip › Images/Fig_S7.pdf]

■ Capacitive response ( $\Delta C$ )

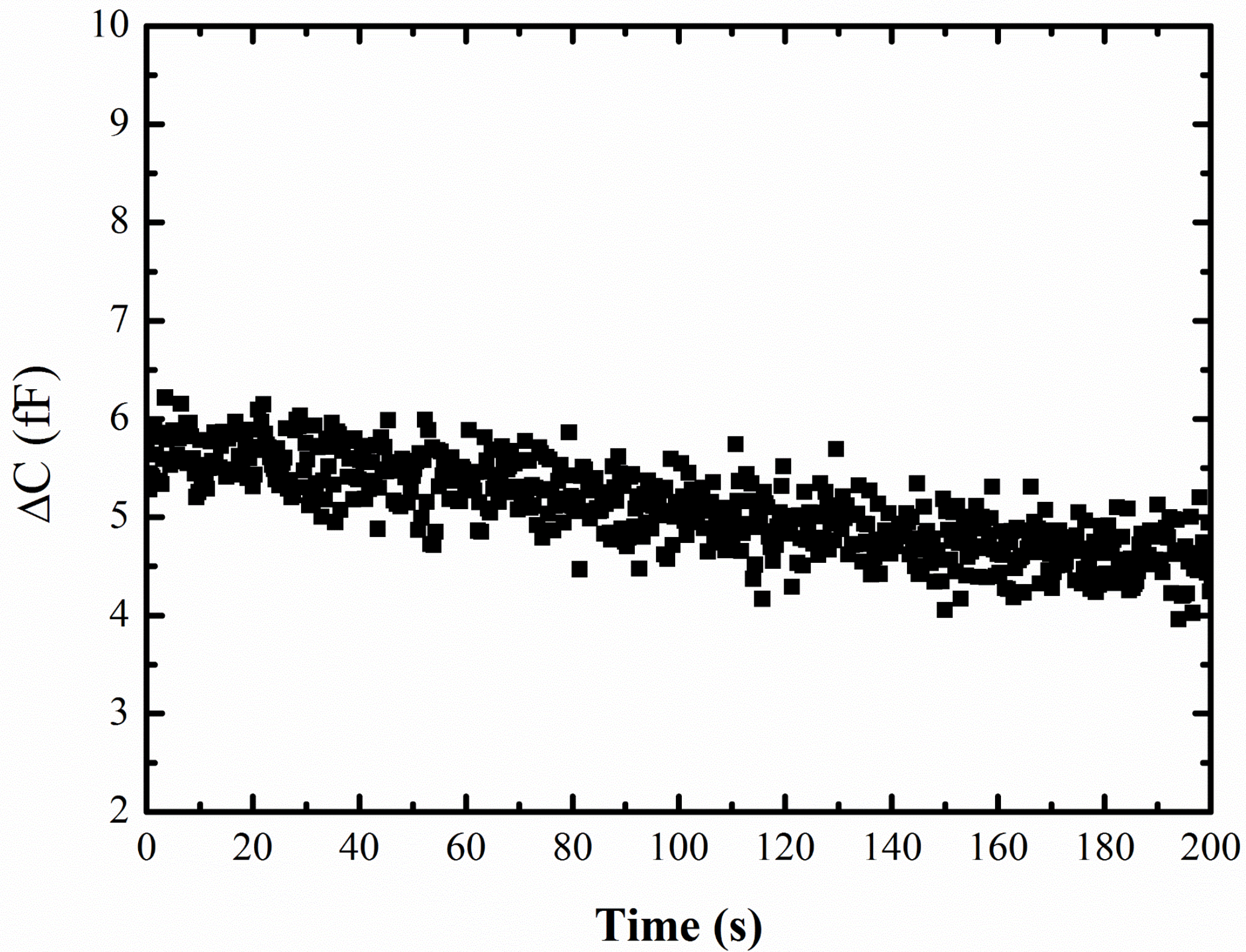

Supplement: Supplementary file 1 [file sensors-19-00888-s001.zip › Images/Fig_S8.pdf]

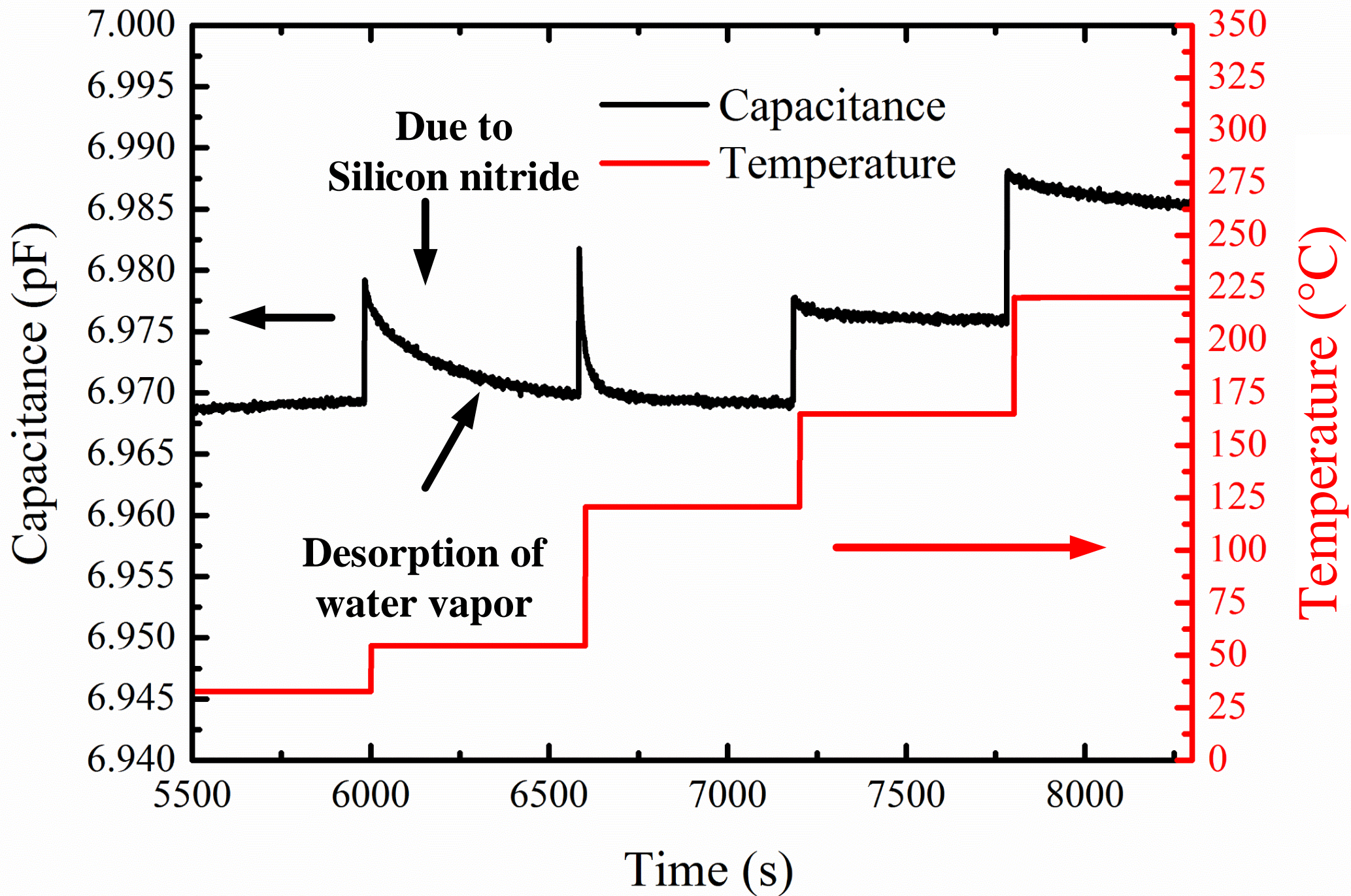

Supplement: Supplementary file 1 [file sensors-19-00888-s001.zip › Images/Fig_S9.pdf]

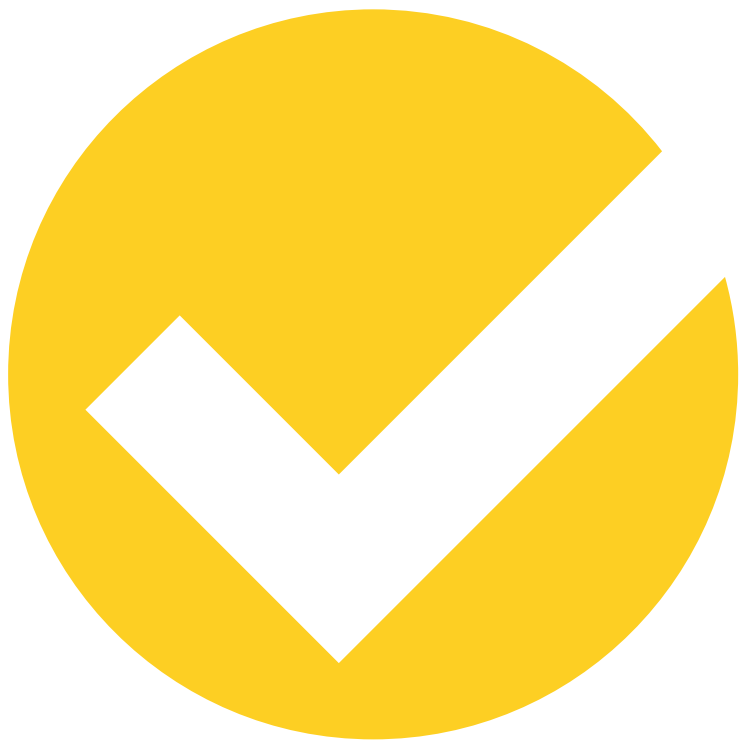

check for  
updates

Supplement: Supplementary file 1 [file sensors-19-00888-s001.zip › logo-updates.pdf]
